# Pharmacokinetics and bioequivalence of two imidocarb formulations in cattle after subcutaneous injection

**DOI:** 10.1371/journal.pone.0270130

**Published:** 2022-06-24

**Authors:** Honglei Wang, Chen Chen, Maolin Liu, Xiaojie Chen, Chunshuang Liu, Yanyan Feng, Xinbo Yan, Yiming Liu, Xiubo Li

**Affiliations:** 1 National Feed Drug Reference Laboratories, Feed Research Institute, Chinese Academy of Agricultural Sciences, Beijing, China; 2 Laboratory of Quality & Safety Risk Assessment for Products on Feed-origin Risk Factor, Ministry of Agriculture and Rural Affairs, Beijing, China; 3 Qilu Animal Health Products Corp. LTD, Shangdong Province, China; 4 College of Veterinary Medicine, China Agricultural University, Beijing, China; Central University of Rajasthan, INDIA

## Abstract

Imidocarb (IMD) is commonly used for treatment of eperythrozoon, babesia, piroplasma and trypanosoma in animals, but there are few studies on its pharmacokinetics in cattle. The purpose of this study was to obtain pharmacokinetic parameters and assess the bioequivalence of subcutaneous injections of two IMD formulations in cattle. Forty-eight healthy cattle, 24 males and 24 females, were randomLy divided into two groups (test group and reference group) with 12 males and 12 females per group. The generic IMD was injected subcutaneously with a single dose of 3.0 mg/kg in the test group. Reference group animals were given one injection of the marketed IMD at the same dosage. The limit of detection (LOD) and limit of quantification (LOQ) for IMD in cattle plasma were 0.05 ng/mL and 0.1 ng/mL, respectively. The recoveries ranged from 88.50% to 92.42%, and the equation of this calibration curve was Y = 13672.1X+187.43. The pharmacokinetics parameters of the test group showed that the maximum concentration of 2257.5±273.62 ng/mL was obtained at 2.14±0.67 h, AUC_0-t_ 14553.95±1946.85 ng·h/mL, AUC_∞_ 15077.88±1952.19 ng·h/mL, T_1/2_ 31.77±25.75 h, CL/F 0.14±0.02 mL/h/g, and V_z_/F 6.53±5.34 mL/g. There was no significant difference in AUC_0-t_, AUC_∞_ and C_max_ between the test group and the reference group (*P*>0.05). The 90% confidence interval of AUC_0-t_, AUC_0-∞_ and C_max_ in the test group was included in 80%–125% AUC_0-t_, AUC_0-∞_ and 70%–143% C_max_ in the reference group, respectively. Based on these results, the two preparations were found to be bioequivalent.

## Introduction

Eperythrozoon is a genus of common bacteria, formerly classified in the Rickettsiaceae that can cause infectious anthropozoonosis [1, 2]. Thousands of cattle are infected with eperythrozoonosis annually and severe infections often result in mortality. Methods of prevention and treatment of eperythrozoonosis involve keeping healthy cattle away from the endemic regions, regular insecticide treatments to prevent insects from biting cattle and the use of effective drugs [3–5]. IMD is a drug used to treat protozoan parasites of animals including diseases such as eperythrocytes, babesia, piroplasma and trypanosoma. Its chemistry belongs to the family of carbanilide derivatives (3,3′-bis (2-imidazolin-2-yl)-carbanilide). It has been used for more than 40 years for treatment and prophylaxis of protozoal diseases including babesiosis and anaplasmosis in farm animals [6–8]. It has been reported to successfully cure cattle with very severe infections of *Babesia bigemina* by intramuscular and intravenous injection at 1 mg/kg·bw. However, an intravenous injection of 3 mg/kg·bw may cause cattle mortality due to its acute toxicity [9]. Although IMD has a long history of animal use, there are few reports on the pharmacokinetics and the bioequivalence with subcutaneous administration in cattle. The objective of this study was to determine the pharmacokinetics and assess the bioequivalence of the two IMD formulation injections in cattle using a single subcutaneous injection of 3.0 mg/kg. With the pharmacokinetic parameters calculated from this studies, different dosage regimens formulated for IMD can be designed and used for clinical treatment. Based on the result of bioequivalence, the generic formulation of IMD could be approved to be marketed in China, and substitute the original product in some respects.

## Materials and methods

### Animals

Twenty-four healthy male cattle and 24 healthy female cattle, aged almost 6 months and weighing 180±15 kg, were selected. The cattle received no treatment for several months prior to the study and were housed in open-air pens. The cattle had free access to water and were fed with a conventional feed without antibiotics during the study. These 48 cattle were divided into 2 groups (test group and reference group) with 24 cattle (12 males and 12 females) in each group. The animals in the test group were treated subcutaneously by a single dose injection with a generic IMD formulation, at the recommended dosage 3.0 mg/kg. Animals in the reference group were injected subcutaneously with a marketed IMD formulation in the same dose as the test group. All cattle experiment procedures were approved and performed in accordance with the Animal Use and Care Committee of Feed Research Institute, Chinese Academy of Agricultural Sciences (number:FRI-CAAS20150811). There was no anesthesia and euthanasia in our study. All cattle were alive and healthy after the whole experiment.

### Drug formulation

The test drug (the generic IMD, 100 mL:85 mg) was manufactured and provided by Qilu Animal Health Products Corp. LTD (Shangdong province, China). The reference drug (the marketed IMD, 100 mL:85 mg) was manufactured and provided by AKZO-NOBEL Corp. (Boxmeer, Netherlands).

### Sample collection

Blood samples (10–15 mL) were collected from the jugular vein at 0 h before administration and 10 min, 30 min, 1, 2, 4, 6, 8, 10, 12, 24, 48, 72 and 96 h after subcutaneous administration, and were drawn in vacutainers containing disodium EDTA as anticoagulant. The samples were immediately centrifuged at 1500 g for 10 min. All the plasma samples in plastic vials were stored at −20°C until they were analyzed.

### Sample preparation

We used a weak cation-exchange solid phase extraction procedure described by Tarbin [10], and made some modifications to determine the amount of IMD in the plasma. Briefly, 1.0 mL plasma samples were added to plastic centrifuge tubes, adding 3 mL methanol/acetonitrile (90/10, v/v), vortexing for 1 min, ultrasonicating for 15 min, and centrifuging for 10 min at 7000 g. To improve IMD recoveries, the residues were extracted twice. The second extracted solution was combined with the first. The combined extracted solution was passed through SPE columns (Waters Oasis WCX, 3cc 60 mg, Waters Company, USA) conditioned with 3 mL methanol and 3 mL water. The loaded cartridge was washed with 3 mL methanol/water (50/50, v/v), and was eluted with 3 mL methanol/formic acid (96/4, v/v). The analyte was evaporated under a nitrogen stream at 40°C, reconstituted in 1 mL methanol/water (15/85, v/v), and filtered through a 0.22 μm nylon syringe filter before analysis by an ultra-performance chromatography-electrospray tandem mass spectrometry (UPLC-MS/MS).

### Analytical assays

Gummow [11] used a high-performance liquid chromatographic (HPLC) method to determine the concentration of diminazene in cattle plasma. We referred to its chromatographic conditions and made modifications to improve the resolution of the IMD peak. The conditions for the UPLC analysis were as follows. Separation was obtained with a C_18_ reverse-phase column (Waters Acquity UPLC^®^ BEH Phenyl 50 mm×2.1 mm, 1.7 μm). The injection volume was 2 μL (partial loop in needle overfill mode). The mobile phases were methanol (phase A) and 0.1% formic acid (phase B). The analyses were conducted at a flow rate of 0.35 mL/min in a linear gradient elution: 0 to 1.5 min 15% phase A; 1.5 to 3 min 90% phase A; 3 to 5 min 15% phase A; 5 to 6 min 15% phase A. The column temperature was controlled at 35±0.5°C. The mass spectrometry (MS) instrumentation is a triple-quadrupole, and its conditions were as follows. The electrospray ionization source was operated in the positive ion mode at a capillary voltage of 2.43 kV. Cone voltage and collision energy for IMD were 30 V and 16 eV. Argon was used as collision gas and nitrogen was used as the nebulizing and desolvation gas. The desolvation temperature was 500°C, and its gas flow was 850 L/h. IMD was determined by multiple reaction monitoring (MRM) using mass-to-charge (m/z) transitions of IMD deprotonated ion ([M+2H]^2+^) of 175→162 and 175→188. 175→162 was used as a quantitative ion. MassLynx Version 4.0 software running under the Microsoft Windows 7 Professional environment was used to operate the instruments and perform data acquisition and data processing for automatic quantification.

### Validation parameters

#### Selectivity and matrix effect

Selectivity was examined by comparing the chromatograms of blank cattle plasma with those of corresponding plasma samples spiked with IMD to exclude the interfering peaks [12]. The matrix effect was evaluated by comparing the area response of extracted blank plasma samples spiked with IMD with the equivalent concentration of IMD standard solution that was dried directly and reconstituted with the same mobile phase [13].

#### LOD and LOQ

The limits of detection (LOD) and the limits of quantitation (LOQ) were determined by drug-free matrix spiked with known concentrations of IMD, whose lowest concentration met the requirement of a signal-to-noise ratio of ≥3 and ≥10, respectively.

#### The calibration curve

A six-point calibration curve was generated using blank plasma spiked with IMD at the following concentrations: 0.1, 1, 5, 10, 20 and 50 ng/mL, which was constructed by plotting the peak area of IMD (y) vs the nominal concentration of IMD (x) in the form of Y = aX+b; the least square method was used for the linear regression analysis. A coefficient of correlation (r) with at least 0.99 was required to meet the criterion. The samples of IMD concentration above the highest concentration level (50 ng/mL) of the analytical curve should dilute to a reasonable multiple.

#### Accuracy and precision

The precision was regarded as the relative standard deviation (RSD) of replicate measurements of spiked sample, and the accuracy was evaluated by the ratio of calculated vs. theoretical concentrations, as previously described [14]. In this study, the accuracy and precision of the method was determined by blank plasma spiked with three concentrations (0.2, 10 and 50 ng/mL). The intra-day accuracy and precision of the UPLC/MS/MS method were determined by analyzing QC concentrations (0.2, 10 and 50 ng/mL) in five replicates per concentration on the same day. Inter-day accuracy and precision were evaluated by analyzing QC concentrations (0.2, 10 and 50 ng/mL) in five measurements of each concentration conducted over five days [15]. We calculated the recoveries and relative standard deviations (RSDs) to evaluate accuracy and precision, respectively. According to the ICH [16], the criterion for precision and accuracy was an RSD≤15% for each concentration.

### Pharmacokinetics

Plasma concentrations versus time for each cow were analyzed using WinNonlin 8.1 (Pharsight Corporation, Mountain View, CA, USA) software that provides noncompartmental analyses of the experimental data. Least-squares nonlinear regression was used to fit the pharmacokinetic parameters to the weighed (y = l/y^2^) experimental data. The pharmacokinetic parameters of IMD for each cow were obtained, and the main pharmacokinetic parameters were compared between the two groups. Also, the three pharmacokinetic parameters of AUC_0-t_, AUC_∞_ and C_max_ were used to analyze the bioequivalence of the two preparations.

### Bioequivalence analysis

The main pharmacokinetic parameters (AUC, C_max_) are statistically analyzed and the bioequivalence are evaluated. It should be within the standard bioequivalence acceptance ranges according to Guidance for Industry BIOEQUIVALENCE GUIDANCE of the FDA guidelines [17]. The specific requirements are as follows: the AUC and C_max_ should be transformed in log form and then analysis of variance and two one-sided references should be performed. If the 90% confidence interval of AUC of the test preparation falls within 80%–125% AUC of the reference preparation and the 90% confidence interval of C_max_ falls within 70%–143% C_max_ of the reference preparation, the test preparation would be considered bioequivalent with the reference preparation.

## Results

### Validation parameters

#### Selectivity and matrix effect

**Fig 1:**
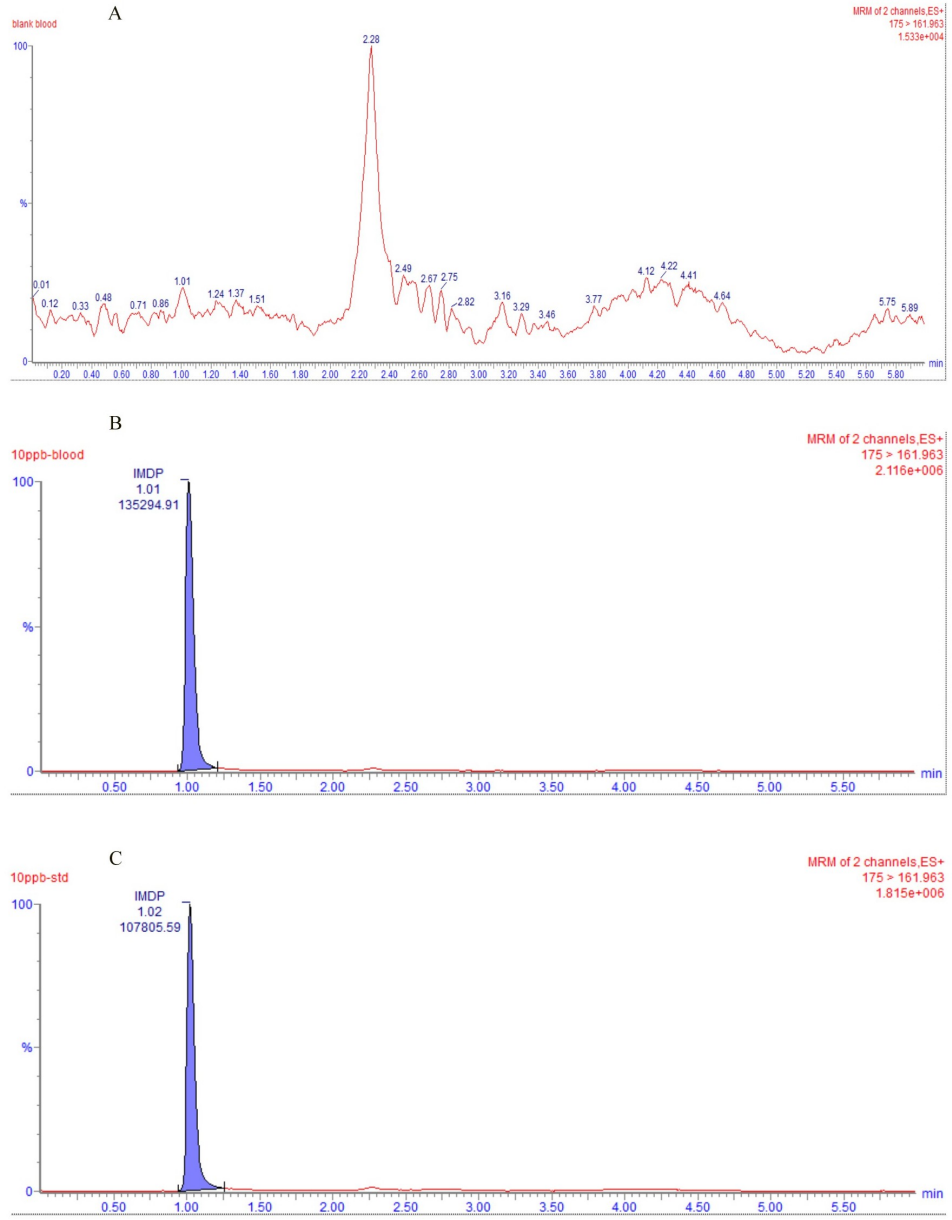
UPLC-MS/MS chromatograms. (A) blank plasma sample, (B) blank plasma sample spiked with IMD (10 ng/mL), (C) 10 ng/mL standard IMD solution.

The specificity of the method was assessed by analyzing blank cattle plasma sample using the experimental conditions described above, and the chromatogram of blank cattle plasma was shown in Fig 1A. The blank plasma sample didn’t exhibit endogenous substancemediated interference in the retention time of IMD. In an evaluation of the effect of the plasma matrix on IMD, the chromatograms of 10 ng/mL standard IMD solution and blank sample spiked with 10 ng/mL standard IMD solution were compared, and their chromatograms were shown in Fig 1B and 1C. It concludes that the cattle plasma matrix could enhance peak area of IMD. Therefore, we should prepare the calibration standards in the same biological matrix as the samples in the intended study.

#### LOQ and linearity

**Fig 2:**
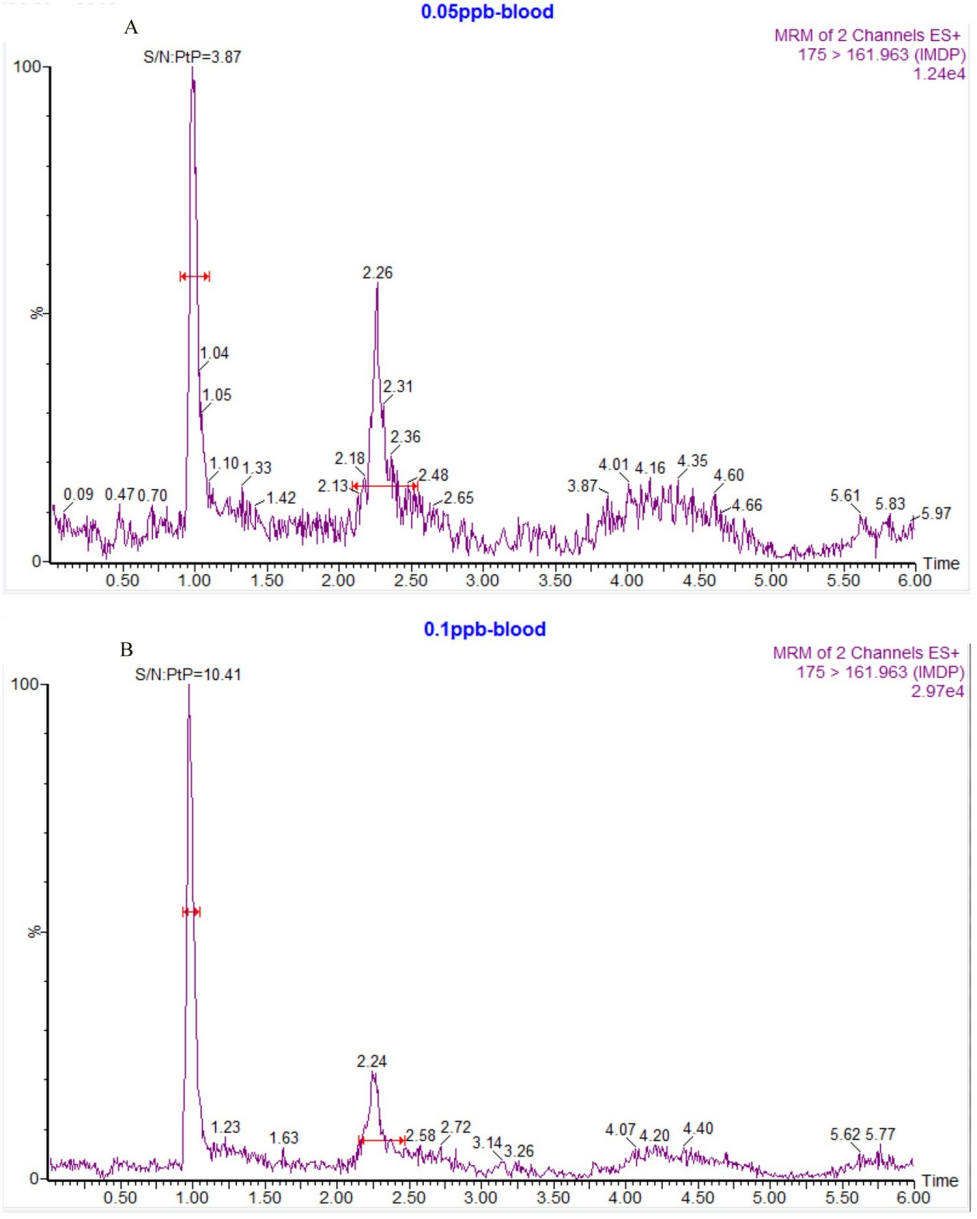
UPLC-MS/MS chromatograms. (A) LOD, (B) LOQ.

**Fig 3:**
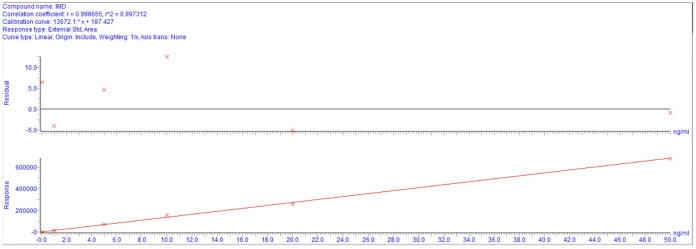
The calibration curves for IMD.

The LOD and LOQ for IMD in cattle plasma were 0.05 ng/mL and 0.1 ng/mL, and their chromatograms were shown in Fig 2A and 2B, respectively. The calibration curve for IMD was linear over the concentration range of 0.1–50 ng/mL according to the results of a weighted (1/x^2^) least-square linear regression analysis. The calibration curves are shown in Fig 3. The extrapolated equation of the calibration curve for IMD was Y = 13672.1X+187.43 (r = 0.9986) for concentrations ranging from 0.1–50 ng/mL, where Y is the peak area and X is the concentration of IMD in ng/mL.

#### Accuracy and precision

The results of the analyses of the recovery, the coefficients of variation (CV) for inter-day and intra-day values of the QC samples (0.2, 10 and 50 ng/mL) were shown in Table 1. The recoveries of the three concentrations ranged from 88.50% to 92.42%. The coefficients of variation (CV) for inter-day and intra-day values were 3.66%–5.60% and 2.15%–7.39%.

**Table 1:** The average recovery, intra RSD and inter RSD of IMD in cattle plasma at 3 spiked concentrations.

Spiked Concentration (ng/mL)	Average Recovery (%)	SD (%)	Intra RSD (%)					Inter RSD (%)
			1 d	2 d	3 d	4 d	5 d	
0.2	88.50	4.23	5.12	4.18	3.84	5.38	5.41	4.62
10	92.42	5.46	2.15	7.39	4.27	5.07	5.32	5.60
50	92.19	3.86	3.58	2.77	2.63	2.69	2.73	3.66

Note: SD, standard deviation; RSD, relative standard deviation.

### Plasma pharmacokinetics

**Fig 4:**
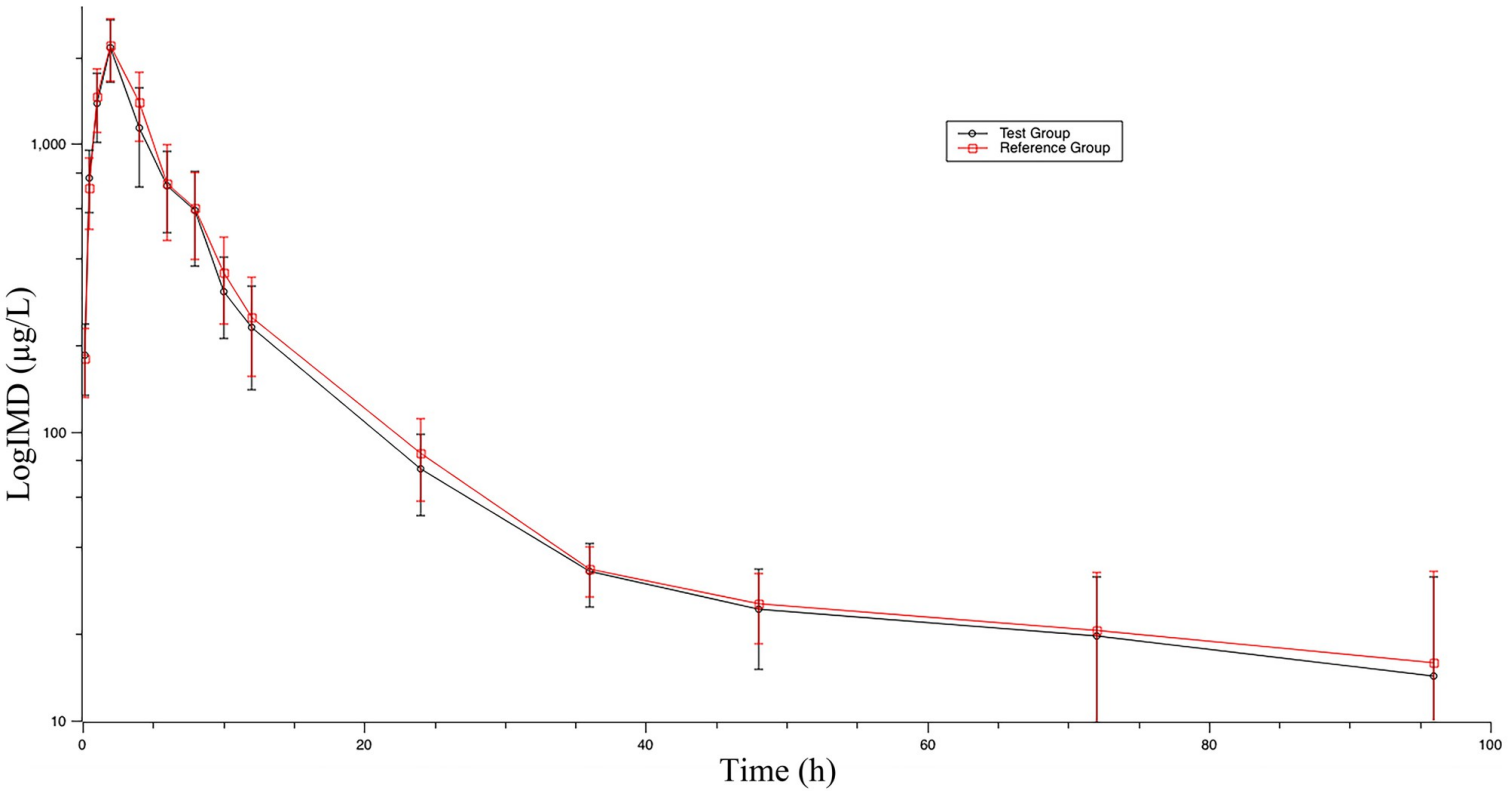
Plasma concentrations (mean+SD) of IMD in cattle following a single subcutaneous injection of 3.0 mg/kg. Error bars represent standard deviations.

The average concentrations of IMD in cattle plasma at different sampling time points are shown in Table 2. The mean plasma IMD concentration-time profile following a single subcutaneous injection of 3.0 mg/kg is presented in Fig 4, and the mean values of pharmacokinetic parameters for the drug are shown in Table 3. IMD was quickly absorbed into the plasma. For the test group, the maximum concentration of 2257.5±273.62 ng/mL was obtained at 2.14±0.67 h, AUC_0-t_ was 14553.95±1946.85 ng·h/mL, AUC_∞_ was 15077.88±1952.19 ng·h/mL, and apparent distribution volume was 6.53±5.34 mL/g. For the reference group, the maximum concentration of 2288.33±277.88 ng/mL was obtained at 1.96±0.20 h, AUC_0-t_ was 15631.69±1698.03 ng·h/mL, AUC_0-∞_ was 16323.61±1781.23 ng·h/mL, and apparent distribution volume was 7.35±2.99 mL/g. No adverse events were found or reported in test and reference groups throughout the whole study.

**Table 2:** Average concentration of IMD in cattle plasma at different sampling time points in a test group and a reference group (ng/mL)n = 24.

Time (h)	0.167	0.5	1	2	4	6	8	10	12	24	36	48	72	96
Test Group	193.1	795.6	1442.5	2257.5	1189.2	747.5	613.2	320.2	239.9	77.2	32.8	23.3	17.4	10.9
Reference Group	188.4	729.5	1517.5	2282.1	1455.6	760.3	621.8	370.8	260.5	87.1	33.3	24.5	18.5	12.6

**Table 3:** The main pharmacokin etic parameters of IMD in the test and reference groups.

Parameters	Units	Test group	Reference group
C_max_	ng/mL	2257.5±273.62	2288.33±277.88
T_max_	h	2.14±0.67	1.96±0.20
AUC_0-t_	μg·h/L	14553.95±1946.85	15631.69±1698.03
AUC_∞_	μg·h/L	15077.88±1952.19	16323.61±1781.23
MRT	h	11.11±1.04	11.18±0.86
T_1/2_	h	31.77±25.75	39.15±15.17
CL/F	mL/h/g	0.14±0.02	0.13±0.02
V_z/_F	mL/g	6.53±5.34	7.35±2.99

Note: C_max_, peak drug concentration; T_max_, time to reach C_max_ from time zero; AUC_0–t_, the area under the concentration-time curve from zero to defining time; AUC_∞_, the total area under the concentration-time curve from zero to infinity; MRT, mean residence time; T_1/2_, the half-life; CL/F, system clearance; V_z/_F, the apparent volume of distribution.

### Bioequivalence

One way ANOVA results showed no significant difference in AUC_0-t_ (*P* = 0.28>0.05), AUC_∞_ (*P* = 0.17>0.05) and C_max_ (*P* = 0.66>0.05) between the test group and the reference group. The result of bilateral unilateral T-test results for AUC_0-t_ were t_1_ = 2.69 and t_2_ = 2.01. The 90% confidence interval of AUC_0-t_ was [4.13, 4.20] in the test group. Bilateral unilateral T-test was also used for AUC_0-∞_, which was used to verify the conclusion of bioequivalence. T-test results for AUC_0-∞_ were t_1_ = 2.69 and t_2_ = 2.02. The 90% confidence interval of AUC_0-∞_ was [4.15, 4.19] in the test group. The results of bilateral unilateral T-test for C_max_ were t_1_ = 2.69 and t_2_ = 2.01. The 90% confidence interval of C_max_ was [3.33, 3.37] in the test group. For the reference group, the 80%–125% of the AUC_0-t_ and AUC_0-∞_ was [3.35, 5.24] and [3.37, 5.26], respectively. The 70%–143% of C_max_ was [2.35, 4.80]. The 90% confidence interval of AUC_0-t_, AUC_0-∞_ and C_max_ of the test preparation falls within 80%–125% AUC_0-t_, AUC_0-∞_ of the reference preparation and 70%–143% C_max_ of the reference preparation respectively. The results show that the two preparations are bioequivalent.

## Discussion

Many ruminants are infected with tick-borne hemiparasite diseases, including mycoplasma bovis, babesiosis, ehrlichiosis and anaplasmosis. Sick animals often have appetite loss, emaciation, swelling of superficial lymph nodes, and have sub-acute inflammation of various organs including the udder, joints, middle ear, as well as the respiratory and genital tract. They may also have high fever and suffer mortality from the disease. These diseases can cause large economic losses. IMD is an effective drug that can cross cellular membranes, such as the blood-brain barrier and the blood-milk barrier [18–23]. The intracellular pH is lower than the extracellular pH, which causes protonation in the intracellular space for lipid soluble, basic drugs. Ionic trapping in rumen fluid (pH 5.5 to 6.5) can also provide a good environment for drug movement into intracellular areas [18]. IMD can resist biotrasformative processes and also bind to the nuclear components of cells. This causes large drug deposits and prolongs its persistence time in the animal body, especially in the liver and kidney [23–25]. There are few reports on the plasma concentrations of IMD that are effective for treatment and prevention of babesiosis and none are available for the Babesia spp. of small ruminants. Kuttler reported that “small” babesia organisms are more difficult to treat compared with “large” ones, and found that there are differences in sensitivity among various species of small babesia [6, 26].

The dosage of IMD used for the treatment and prevention of babesiosis in cattle ranges from 1.0 to 3.0 mg/kg when it is administered by intramuscular or subcutaneous injection [27]. In this study, a 3.0 mg/kg dose was chosen to study the pharmacokinetics and bioequivalence of IMD in cattle. None of the cattle showed adverse effects after IM administration in this study, but it has been reported that cattle have died from bronchoconstriction secondary to cholinesterase inhibition after intravenous injection of IMD [28].

According to the abundance of [M+H]^+^ of the drug molecules in ESI^+^ ionization mode, the ion (m/z = 349) was regarded as the parent ion. After optimizing the mass spectrum parameters of parent ion (m/z = 349), the secondary scanning mode was used to find the daughter Ion. The daughter ions (m/z = 188) and (m/z = 162) were chosen as the quantitative ion and the qualitative ion, respectively. But, in the actual sample determination process, it is found that when the ion (m/z = 349) was used as the parent ion, the intensity of matrix effect was enhanced, which seriously interfered with the actual detection of the sample. IMD is easy to ionize in aqueous solution, which readily produces both a monoprotonated m/z 349 [M+H]^+^ ion and a diprotonated m/z 175 [M+2H]^2+^ ion. In practical operation, but m/z 175 is more plentiful and stable for greater sensitivity. Therefore, the diprotonated m/z 175 [M+2H]^2+^ was finally set to the parent ion in this study, which is consistent with Lehner ’s study results [29].

In the validation study, the selectivity, linearity, LOD, LOQ, accuracy and precision were assessed according to guidelines established by the US Food and Drug Administration for bioanalytical method validation [30]. No endogenous substance-mediated interference was observed for the retention time of IMD. The LOD and LOQ for IMD in cattle plasma were 0.05 ng/mL and 0.1 ng/mL, respectively, which were lower than those reported by Belloli [22, 31]. A good linear relationship between the IMD concentration and quantitative ion peak area was established. The coefficients of variation (CV) for inter-day and intra-day values were 3.66%–5.60% and 2.15%–7.39%, which were below 10%. Based on the experimental parameters, we conclude that the method for quantifying IMD in cattle plasma was stable and precise.

In the test group, IMD was quickly absorbed and detected in all treated animals in 10 min after administration. A plateau of the concentration was 2257.5±273.62 ng/mL in 2.14±0.67 h. It was eliminated very slowly with T_1/2_ 31.77±25.75 h, which is much longer than elimination in swine (T_1/2_ 13.91±2.73 h) [1], dogs (T_1/2_ 3.45±0.75 h) and goats (T_1/2_ 4.18±1.57 h, 7.73±1.73 h) [32], horses (5.14±2.84 h) [31] and white-tailed deer (7.73±1.73 h) [7]. This may be due to the differences in animal species and administration routes. The apparent volume of distribution (V_d_) of IMD in cattle was 6.53±5.34 mL/g, which is similar to results with sheep (4.18±0.44 mL/g) and goats (7.68±0.57 mL/g) [22]. Compared to previous studies, this suggests that the distribution volume of IMD is very large. IMD is a lipid-soluble organic base, which can bind to protons within cells (including erythrocytes). The intracellular pH is lower than the pH of plasma, which may contribute to the large volume of distribution of IMD. The apparent clearance rate in cattle was low (0.18±0.02 mL/h), but it was comparable to that found in sheep and goats [22]. The low apparent clearance rate, coupled with the large volume of distribution, would help explain the relatively slow depletion of IMD from the plasma.

Establishing a rational dosage regimen for cattle requires knowledge of the plasma concentrations of IMD that may be effective for treatment and prophylaxis of babesiosis. *Babesia odocoilei* is morphologically similar to the “large” Babesia spp such as *B*. *bigemina*, *B*. *caballi* and *B*. *divergens* [33]. The minimum inhibitory concentration of IMD for *B*. *divergens* is 27.0–34.0 ng/mL [34]. In this study, IMD was detectable in all cattle, at a mean plasma concentration of 75.02±23.54 ng/mL at 24 h after subcutaneous administration. Therefore, subcutaneous injection at 3.0 mg/kg for cattle may be efficacious for the treatment of *B*. *odocoilei*, and the efficacy will last at least 24 h due to the rapid distribution and slow elimination of IMD in cattle. Clinical trials of cattle infected with *B*. *odocoilei* are needed to evaluate the efficacy of the 3.0 mg/kg dose of IMD for the treatment of cervid babesiosis. Because IMD has a long withdrawal time in edible tissues, the residues in muscle, liver, kidney and fat were also detected (unpublished data) and will be published elsewhere.

After log conversion of the AUC_0-t_, AUC_0-∞_ and C_max_ data, it was analyzed with one-way ANOVA for AUC_0-t_, AUC_0-∞_ and C_max_ between the test group and reference group. The results were as follows. There was no significance difference between the test group and reference group for AUC_0-t_ (*P* = 0.277>0.05), AUC_0-∞_(*P* = 0.170>0.05) and C_max_ (*P* = 0.664>0.05). The 90% confidence interval of AUC_0-t_, AUC_0-∞_ and C_max_ in the reference group was included in 80%–125% AUC_0-t_, AUC_0-∞_ and 70%–143% C_max_ in the reference group, respectively. Based on comparing the three pharmacokinetic parameters, we concluded that the two preparations are bioequivalent.

## Conclusion

A processing method and UPLC-MS/MS parameters were established for the detection of IMD in cattle plasma. IMD was absorbed quickly into cattle blood and reached a plateau in about 2 h when subcutaneously administered at 3.0 mg/kg. It eliminated very slowly from the blood with a large T_1/2_, and the apparent volume of distribution was also large in cattle. These results provide guidance for the design of dosage regimens that may be useful to treat cattle infected with *B*. *odocoilei* parasitemia. Comparing the pharmacokinetic parameters AUC_0-t_, AUC_0-∞_ and C_max_ in the test group and reference group, it concludes that the test formulation of generic IMD is bioequivalent to the reference formulation. Also, the generic IMD could be used to offer an alternative to the marketed IMD formulation.

## Supporting information

S1 Data (XLSX)

S1 File (DOC)

S2 File (DOC)
