# Supplementary material for: Pharmacokinetics and bioequivalence of two imidocarb formulations in cattle after subcutaneous injection
Source: PLoS One. 2022 Jun 24;17(6):e0270130. doi: 10.1371/journal.pone.0270130 (PMC9231748; doi:10.1371/journal.pone.0270130)
Supplement: S1 File — (DOC) [file pone.0270130.s002.doc]

| **Animal** | **Rsq** | **Rsq_adjusted** | **Corr_XY** | **No_points_lambda_z** | **Lambda_z (1/hr)** | **Lambda_z_lower (hr)** | **Lambda_z_upper (hr)** | **HL_Lambda_z (hr)** | **Tlag (hr)** | **Tmax (hr)** | **Cmax (ug/L)** | **Cmax_D (kg*ug/L/mg)** | **Tlast (hr)** | **Clast (ug/L)** | **AUClast (hr*ug/L)** | **AUCall (hr*ug/L)** | **AUCINF_obs (hr*ug/L)** | **AUCINF_D_obs (hr*kg*ug/L/mg)** | **AUC_%Extrap_obs (%)** | **Vz_F_obs (L/kg)** | **Cl_F_obs (L/hr/kg)** | **AUCINF_pred (hr*ug/L)** | **AUCINF_D_pred (hr*kg*ug/L/mg)** | **AUC_%Extrap_pred (%)** | **Vz_F_pred (L/kg)** | **Cl_F_pred (L/hr/kg)** | **AUMClast (hr*hr*ug/L)** | **AUMCINF_obs (hr*hr*ug/L)** | **AUMC_%Extrap_obs (%)** | **AUMCINF_pred (hr*hr*ug/L)** | **AUMC_%Extrap_pred (%)** | **MRTlast (hr)** | **MRTINF_obs (hr)** | **MRTINF_pred (hr)** |
| --- | --- | --- | --- | --- | --- | --- | --- | --- | --- | --- | --- | --- | --- | --- | --- | --- | --- | --- | --- | --- | --- | --- | --- | --- | --- | --- | --- | --- | --- | --- | --- | --- | --- | --- |
| 1 | 1.0000 | 1.0000 | -1.0000 | 3.0000 | 0.0185 | 48.0000 | 96.0000 | 37.3771 | 0.0000 | 2.0000 | 2550.0000 | 1200.0000 | 96.0000 | 12.4000 | 17015.2375 | 17015.2375 | 17683.8922 | 8321.8316 | 3.7812 | 6.4798 | 0.1202 | 17684.4505 | 8322.0944 | 3.7842 | 6.4796 | 0.1202 | 191774.4534 | 292021.6980 | 34.3287 | 292105.4015 | 34.3475 | 11.2707 | 16.5134 | 16.5176 |
| 2 | 0.8253 | 0.7380 | -0.9085 | 4.0000 | 0.0115 | 36.0000 | 96.0000 | 60.0778 | 0.0000 | 2.0000 | 2530.0000 | 1190.5882 | 96.0000 | 15.4000 | 15019.6385 | 15019.6385 | 16354.4180 | 7696.1967 | 8.1616 | 11.2619 | 0.1299 | 16214.6498 | 7630.4234 | 7.3699 | 11.3590 | 0.1311 | 161891.7210 | 405721.2350 | 60.0978 | 380189.2131 | 57.4181 | 10.7787 | 24.8081 | 23.4473 |
| 3 | 0.9374 | 0.9165 | -0.9682 | 5.0000 | 0.0294 | 24.0000 | 96.0000 | 23.6058 | 0.0000 | 2.0000 | 2190.0000 | 1030.5882 | 96.0000 | 5.8000 | 14351.5386 | 14351.5386 | 14549.0629 | 6846.6178 | 1.3576 | 4.9741 | 0.1461 | 14576.4375 | 6859.5000 | 1.5429 | 4.9648 | 0.1458 | 153443.6207 | 179132.8233 | 14.3409 | 182693.0571 | 16.0102 | 10.6918 | 12.3123 | 12.5335 |
| 4 | 0.9787 | 0.9681 | -0.9893 | 4.0000 | 0.0203 | 36.0000 | 96.0000 | 34.1076 | 0.0000 | 2.0000 | 2210.0000 | 1040.0000 | 96.0000 | 11.2000 | 15352.5356 | 15352.5356 | 15903.6524 | 7484.0717 | 3.4653 | 6.5749 | 0.1336 | 15922.6960 | 7493.0334 | 3.5808 | 6.5670 | 0.1335 | 186896.7695 | 266922.7234 | 29.9809 | 269687.9838 | 30.6989 | 12.1737 | 16.7837 | 16.9373 |
| 5 | 0.9044 | 0.8805 | -0.9510 | 6.0000 | 0.0350 | 12.0000 | 96.0000 | 19.8031 | 0.0000 | 2.0000 | 2100.0000 | 988.2353 | 96.0000 | 9.6000 | 16378.2872 | 16378.2872 | 16652.5571 | 7836.4975 | 1.6470 | 3.6457 | 0.1276 | 16624.2506 | 7823.1768 | 1.4795 | 3.6519 | 0.1278 | 204607.6292 | 238773.3841 | 14.3089 | 235247.2443 | 13.0244 | 12.4926 | 14.3385 | 14.1508 |
| 6 | 0.9968 | 0.9935 | -0.9984 | 3.0000 | 0.0331 | 48.0000 | 96.0000 | 20.9225 | 0.0000 | 2.0000 | 2180.0000 | 1025.8824 | 96.0000 | 8.4000 | 16362.0844 | 16362.0844 | 16615.6364 | 7819.1230 | 1.5260 | 3.8604 | 0.1279 | 16609.0939 | 7816.0442 | 1.4872 | 3.8619 | 0.1279 | 186290.9771 | 218285.3708 | 14.6571 | 217459.8154 | 14.3331 | 11.3855 | 13.1373 | 13.0928 |
| 7 | 0.8190 | 0.7932 | -0.9050 | 9.0000 | 0.0426 | 6.0000 | 96.0000 | 16.2601 | 0.0000 | 2.0000 | 2300.0000 | 1082.3529 | 96.0000 | 15.4000 | 13645.3390 | 13645.3390 | 14006.5986 | 6591.3405 | 2.5792 | 3.5590 | 0.1517 | 13800.4881 | 6494.3473 | 1.1242 | 3.6121 | 0.1540 | 162157.5046 | 205313.0028 | 21.0194 | 180691.3680 | 10.2572 | 11.8837 | 14.6583 | 13.0931 |
| 8 | 0.9867 | 0.9800 | -0.9933 | 4.0000 | 0.0197 | 36.0000 | 96.0000 | 35.1911 | 0.0000 | 2.0000 | 2010.0000 | 945.8824 | 96.0000 | 14.3000 | 13876.5373 | 13876.5373 | 14602.5482 | 6871.7874 | 4.9718 | 7.3882 | 0.1455 | 14566.2128 | 6854.6884 | 4.7348 | 7.4066 | 0.1459 | 191623.8870 | 298180.4995 | 35.7356 | 292847.5565 | 34.5653 | 13.8092 | 20.4198 | 20.1046 |
| 9 | 0.7919 | 0.7659 | -0.8899 | 10.0000 | 0.0496 | 4.0000 | 96.0000 | 13.9879 | 0.0000 | 2.0000 | 2190.0000 | 1030.5882 | 96.0000 | 13.2000 | 12790.0395 | 12790.0395 | 13056.4195 | 6144.1974 | 2.0402 | 3.2844 | 0.1628 | 12873.8883 | 6058.3004 | 0.6513 | 3.3310 | 0.1651 | 137623.0046 | 168571.1215 | 18.3591 | 147364.5854 | 6.6105 | 10.7602 | 12.9110 | 11.4468 |
| 10 | 0.9968 | 0.9937 | -0.9984 | 3.0000 | 0.0206 | 48.0000 | 96.0000 | 33.6961 | 0.0000 | 2.0000 | 2440.0000 | 1148.2353 | 96.0000 | 11.4000 | 14512.0359 | 14512.0359 | 15066.2261 | 7089.9887 | 3.6784 | 6.8566 | 0.1410 | 15075.2063 | 7094.2147 | 3.7357 | 6.8525 | 0.1410 | 175525.0031 | 255668.2063 | 31.3466 | 256966.8613 | 31.6935 | 12.0951 | 16.9696 | 17.0457 |
| 11 | 0.9969 | 0.9954 | -0.9985 | 4.0000 | 0.0227 | 36.0000 | 96.0000 | 30.5131 | 0.0000 | 2.0000 | 1950.0000 | 917.6471 | 96.0000 | 9.3000 | 11114.2843 | 11114.2843 | 11523.6808 | 5422.9086 | 3.5527 | 8.1176 | 0.1844 | 11519.6292 | 5421.0020 | 3.5187 | 8.1205 | 0.1845 | 136696.1942 | 194020.3471 | 29.5454 | 193453.0376 | 29.3388 | 12.2991 | 16.8367 | 16.7933 |
| 12 | 0.9900 | 0.9849 | -0.9950 | 4.0000 | 0.0225 | 36.0000 | 96.0000 | 30.7397 | 0.0000 | 2.0000 | 2110.0000 | 992.9412 | 96.0000 | 6.3000 | 11254.7865 | 11254.7865 | 11534.1786 | 5427.8488 | 2.4223 | 8.1704 | 0.1842 | 11547.8337 | 5434.2747 | 2.5377 | 8.1608 | 0.1840 | 114182.9947 | 153395.1198 | 25.5628 | 155311.5786 | 26.4813 | 10.1453 | 13.2992 | 13.4494 |
| 13 | 0.8650 | 0.8481 | -0.9300 | 10.0000 | 0.0559 | 4.0000 | 96.0000 | 12.4094 | 0.0000 | 2.0000 | 2070.0000 | 974.1176 | 96.0000 | 6.0000 | 12577.6837 | 12577.6837 | 12685.1011 | 5969.4594 | 0.8468 | 2.9991 | 0.1675 | 12620.2479 | 5938.9402 | 0.3373 | 3.0145 | 0.1684 | 126817.5595 | 139052.7242 | 8.7989 | 131665.7534 | 3.6822 | 10.0827 | 10.9619 | 10.4329 |
| 14 | 0.9995 | 0.9991 | -0.9998 | 3.0000 | 0.0143 | 48.0000 | 96.0000 | 48.5494 | 0.0000 | 2.0000 | 1930.0000 | 908.2353 | 96.0000 | 12.8000 | 14776.4848 | 14776.4848 | 15673.0212 | 7375.5394 | 5.7203 | 9.4965 | 0.1356 | 15669.1748 | 7373.7293 | 5.6971 | 9.4988 | 0.1356 | 170447.2776 | 319309.8960 | 46.6201 | 318671.2245 | 46.5131 | 11.5350 | 20.3732 | 20.3375 |
| 15 | 0.8295 | 0.8052 | -0.9108 | 9.0000 | 0.0497 | 6.0000 | 96.0000 | 13.9414 | 0.0000 | 2.0000 | 1930.0000 | 908.2353 | 96.0000 | 6.6000 | 12314.6365 | 12314.6365 | 12447.3837 | 5857.5923 | 1.0665 | 3.4337 | 0.1707 | 12376.5504 | 5824.2590 | 0.5003 | 3.4534 | 0.1717 | 119458.2367 | 134871.9461 | 11.4284 | 126647.2666 | 5.6764 | 9.7005 | 10.8354 | 10.2328 |
| 16 | 0.9092 | 0.8185 | -0.9535 | 3.0000 | 0.0122 | 48.0000 | 96.0000 | 56.9463 | 0.0000 | 2.0000 | 2470.0000 | 1162.3529 | 96.0000 | 12.6000 | 12879.1899 | 12879.1899 | 13914.3577 | 6547.9330 | 7.4396 | 12.5469 | 0.1527 | 13971.0140 | 6574.5948 | 7.8149 | 12.4960 | 0.1521 | 140709.6964 | 325131.2370 | 56.7222 | 335224.8968 | 58.0253 | 10.9254 | 23.3666 | 23.9943 |
| 17 | 0.7907 | 0.7608 | -0.8892 | 9.0000 | 0.0433 | 6.0000 | 96.0000 | 16.0095 | 0.0000 | 2.0000 | 2870.0000 | 1350.5882 | 96.0000 | 12.0000 | 14395.5868 | 14395.5868 | 14672.7494 | 6904.8233 | 1.8890 | 3.3450 | 0.1448 | 14524.1590 | 6834.8984 | 0.8852 | 3.3793 | 0.1463 | 151531.5783 | 184540.7906 | 17.8872 | 166844.1341 | 9.1778 | 10.5263 | 12.5771 | 11.4874 |
| 18 | 0.8410 | 0.8183 | -0.9170 | 9.0000 | 0.0515 | 6.0000 | 96.0000 | 13.4497 | 0.0000 | 2.0000 | 2680.0000 | 1261.1765 | 96.0000 | 8.3000 | 17630.4873 | 17630.4873 | 17791.5395 | 8372.4892 | 0.9052 | 2.3176 | 0.1194 | 17700.5201 | 8329.6565 | 0.3957 | 2.3295 | 0.1201 | 163110.1788 | 181696.2276 | 10.2292 | 171192.2300 | 4.7210 | 9.2516 | 10.2125 | 9.6716 |
| 19 | 0.9044 | 0.8566 | -0.9510 | 4.0000 | 0.0152 | 36.0000 | 96.0000 | 45.5009 | 0.0000 | 2.0000 | 2870.0000 | 1350.5882 | 96.0000 | 14.8000 | 16770.7357 | 16770.7357 | 17742.2648 | 8349.3011 | 5.4758 | 7.8622 | 0.1198 | 17734.5740 | 8345.6819 | 5.4348 | 7.8656 | 0.1198 | 193875.5022 | 350917.2260 | 44.7518 | 349674.0626 | 44.5554 | 11.5603 | 19.7786 | 19.7171 |
| 20 | 0.7820 | 0.7578 | -0.8843 | 11.0000 | 0.0528 | 2.0000 | 96.0000 | 13.1169 | 0.0000 | 2.0000 | 2210.0000 | 1040.0000 | 96.0000 | 13.8000 | 18034.9870 | 18034.9870 | 18296.1348 | 8609.9458 | 1.4273 | 2.1979 | 0.1161 | 18137.3470 | 8535.2221 | 0.5644 | 2.2171 | 0.1172 | 212750.5453 | 242762.6309 | 12.3627 | 224514.1415 | 5.2396 | 11.7965 | 13.2685 | 12.3786 |
| 21 | 0.7630 | 0.7366 | -0.8735 | 11.0000 | 0.0562 | 2.0000 | 96.0000 | 12.3382 | 0.0000 | 2.0000 | 2070.0000 | 974.1176 | 96.0000 | 13.4000 | 16727.9864 | 16727.9864 | 16966.5102 | 7984.2401 | 1.4059 | 2.2294 | 0.1252 | 16792.7263 | 7902.4594 | 0.3855 | 2.2525 | 0.1265 | 168620.0612 | 195764.1420 | 13.8657 | 175987.4815 | 4.1863 | 10.0801 | 11.5383 | 10.4800 |
| 22 | 0.9881 | 0.9762 | -0.9940 | 3.0000 | 0.0052 | 48.0000 | 96.0000 | 132.3882 | 0.0000 | 1.0000 | 1930.0000 | 908.2353 | 96.0000 | 11.2000 | 12461.0380 | 12461.0380 | 14600.1909 | 6870.6781 | 14.6515 | 27.7987 | 0.1455 | 14583.2347 | 6862.6987 | 14.5523 | 27.8310 | 0.1457 | 120022.2705 | 733950.1796 | 83.6471 | 729083.8243 | 83.5379 | 9.6318 | 50.2699 | 49.9947 |
| 23 | 0.8018 | 0.7735 | -0.8955 | 9.0000 | 0.0431 | 6.0000 | 96.0000 | 16.0987 | 0.0000 | 2.0000 | 2140.0000 | 1007.0588 | 96.0000 | 9.6000 | 13224.8850 | 13224.8850 | 13447.8495 | 6328.3998 | 1.6580 | 3.6700 | 0.1580 | 13353.8208 | 6284.1510 | 0.9655 | 3.6959 | 0.1591 | 152043.0110 | 178626.0648 | 14.8820 | 167415.4417 | 9.1822 | 11.4967 | 13.2829 | 12.5369 |
| 24 | 0.9978 | 0.9967 | -0.9989 | 4.0000 | 0.0271 | 36.0000 | 96.0000 | 25.5468 | 0.0000 | 2.0000 | 2250.0000 | 1058.8235 | 96.0000 | 6.9000 | 15753.9869 | 15753.9869 | 16008.2949 | 7533.3153 | 1.5886 | 4.8924 | 0.1327 | 16015.4904 | 7536.7013 | 1.6328 | 4.8902 | 0.1327 | 165265.0116 | 199051.4226 | 16.9737 | 200007.3768 | 17.3705 | 10.4904 | 12.4343 | 12.4884 |

Worksheet: Final Parameters

| **Animal** | **Parameter** | **Units** | **Estimate** |
| --- | --- | --- | --- |
| 1 | Rsq |  | 1.0000 |
| 1 | Rsq_adjusted |  | 1.0000 |
| 1 | Corr_XY |  | -1.0000 |
| 1 | No_points_lambda_z |  | 3.0000 |
| 1 | Lambda_z | 1/hr | 0.0185 |
| 1 | Lambda_z_lower | hr | 48.0000 |
| 1 | Lambda_z_upper | hr | 96.0000 |
| 1 | HL_Lambda_z | hr | 37.3771 |
| 1 | Tlag | hr | 0.0000 |
| 1 | Tmax | hr | 2.0000 |
| 1 | Cmax | ug/L | 2550.0000 |
| 1 | Cmax_D | kg*ug/L/mg | 1200.0000 |
| 1 | Tlast | hr | 96.0000 |
| 1 | Clast | ug/L | 12.4000 |
| 1 | AUClast | hr*ug/L | 17015.2375 |
| 1 | AUCall | hr*ug/L | 17015.2375 |
| 1 | AUCINF_obs | hr*ug/L | 17683.8922 |
| 1 | AUCINF_D_obs | hr*kg*ug/L/mg | 8321.8316 |
| 1 | AUC_%Extrap_obs | % | 3.7812 |
| 1 | Vz_F_obs | L/kg | 6.4798 |
| 1 | Cl_F_obs | L/hr/kg | 0.1202 |
| 1 | AUCINF_pred | hr*ug/L | 17684.4505 |
| 1 | AUCINF_D_pred | hr*kg*ug/L/mg | 8322.0944 |
| 1 | AUC_%Extrap_pred | % | 3.7842 |
| 1 | Vz_F_pred | L/kg | 6.4796 |
| 1 | Cl_F_pred | L/hr/kg | 0.1202 |
| 1 | AUMClast | hr*hr*ug/L | 191774.4534 |
| 1 | AUMCINF_obs | hr*hr*ug/L | 292021.6980 |
| 1 | AUMC_%Extrap_obs | % | 34.3287 |
| 1 | AUMCINF_pred | hr*hr*ug/L | 292105.4015 |
| 1 | AUMC_%Extrap_pred | % | 34.3475 |
| 1 | MRTlast | hr | 11.2707 |
| 1 | MRTINF_obs | hr | 16.5134 |
| 1 | MRTINF_pred | hr | 16.5176 |
| 2 | Rsq |  | 0.8253 |
| 2 | Rsq_adjusted |  | 0.7380 |
| 2 | Corr_XY |  | -0.9085 |
| 2 | No_points_lambda_z |  | 4.0000 |
| 2 | Lambda_z | 1/hr | 0.0115 |
| 2 | Lambda_z_lower | hr | 36.0000 |
| 2 | Lambda_z_upper | hr | 96.0000 |
| 2 | HL_Lambda_z | hr | 60.0778 |
| 2 | Tlag | hr | 0.0000 |
| 2 | Tmax | hr | 2.0000 |
| 2 | Cmax | ug/L | 2530.0000 |
| 2 | Cmax_D | kg*ug/L/mg | 1190.5882 |
| 2 | Tlast | hr | 96.0000 |
| 2 | Clast | ug/L | 15.4000 |
| 2 | AUClast | hr*ug/L | 15019.6385 |
| 2 | AUCall | hr*ug/L | 15019.6385 |
| 2 | AUCINF_obs | hr*ug/L | 16354.4180 |
| 2 | AUCINF_D_obs | hr*kg*ug/L/mg | 7696.1967 |
| 2 | AUC_%Extrap_obs | % | 8.1616 |
| 2 | Vz_F_obs | L/kg | 11.2619 |
| 2 | Cl_F_obs | L/hr/kg | 0.1299 |
| 2 | AUCINF_pred | hr*ug/L | 16214.6498 |
| 2 | AUCINF_D_pred | hr*kg*ug/L/mg | 7630.4234 |
| 2 | AUC_%Extrap_pred | % | 7.3699 |
| 2 | Vz_F_pred | L/kg | 11.3590 |
| 2 | Cl_F_pred | L/hr/kg | 0.1311 |
| 2 | AUMClast | hr*hr*ug/L | 161891.7210 |
| 2 | AUMCINF_obs | hr*hr*ug/L | 405721.2350 |
| 2 | AUMC_%Extrap_obs | % | 60.0978 |
| 2 | AUMCINF_pred | hr*hr*ug/L | 380189.2131 |
| 2 | AUMC_%Extrap_pred | % | 57.4181 |
| 2 | MRTlast | hr | 10.7787 |
| 2 | MRTINF_obs | hr | 24.8081 |
| 2 | MRTINF_pred | hr | 23.4473 |
| 3 | Rsq |  | 0.9374 |
| 3 | Rsq_adjusted |  | 0.9165 |
| 3 | Corr_XY |  | -0.9682 |
| 3 | No_points_lambda_z |  | 5.0000 |
| 3 | Lambda_z | 1/hr | 0.0294 |
| 3 | Lambda_z_lower | hr | 24.0000 |
| 3 | Lambda_z_upper | hr | 96.0000 |
| 3 | HL_Lambda_z | hr | 23.6058 |
| 3 | Tlag | hr | 0.0000 |
| 3 | Tmax | hr | 2.0000 |
| 3 | Cmax | ug/L | 2190.0000 |
| 3 | Cmax_D | kg*ug/L/mg | 1030.5882 |
| 3 | Tlast | hr | 96.0000 |
| 3 | Clast | ug/L | 5.8000 |
| 3 | AUClast | hr*ug/L | 14351.5386 |
| 3 | AUCall | hr*ug/L | 14351.5386 |
| 3 | AUCINF_obs | hr*ug/L | 14549.0629 |
| 3 | AUCINF_D_obs | hr*kg*ug/L/mg | 6846.6178 |
| 3 | AUC_%Extrap_obs | % | 1.3576 |
| 3 | Vz_F_obs | L/kg | 4.9741 |
| 3 | Cl_F_obs | L/hr/kg | 0.1461 |
| 3 | AUCINF_pred | hr*ug/L | 14576.4375 |
| 3 | AUCINF_D_pred | hr*kg*ug/L/mg | 6859.5000 |
| 3 | AUC_%Extrap_pred | % | 1.5429 |
| 3 | Vz_F_pred | L/kg | 4.9648 |
| 3 | Cl_F_pred | L/hr/kg | 0.1458 |
| 3 | AUMClast | hr*hr*ug/L | 153443.6207 |
| 3 | AUMCINF_obs | hr*hr*ug/L | 179132.8233 |
| 3 | AUMC_%Extrap_obs | % | 14.3409 |
| 3 | AUMCINF_pred | hr*hr*ug/L | 182693.0571 |
| 3 | AUMC_%Extrap_pred | % | 16.0102 |
| 3 | MRTlast | hr | 10.6918 |
| 3 | MRTINF_obs | hr | 12.3123 |
| 3 | MRTINF_pred | hr | 12.5335 |
| 4 | Rsq |  | 0.9787 |
| 4 | Rsq_adjusted |  | 0.9681 |
| 4 | Corr_XY |  | -0.9893 |
| 4 | No_points_lambda_z |  | 4.0000 |
| 4 | Lambda_z | 1/hr | 0.0203 |
| 4 | Lambda_z_lower | hr | 36.0000 |
| 4 | Lambda_z_upper | hr | 96.0000 |
| 4 | HL_Lambda_z | hr | 34.1076 |
| 4 | Tlag | hr | 0.0000 |
| 4 | Tmax | hr | 2.0000 |
| 4 | Cmax | ug/L | 2210.0000 |
| 4 | Cmax_D | kg*ug/L/mg | 1040.0000 |
| 4 | Tlast | hr | 96.0000 |
| 4 | Clast | ug/L | 11.2000 |
| 4 | AUClast | hr*ug/L | 15352.5356 |
| 4 | AUCall | hr*ug/L | 15352.5356 |
| 4 | AUCINF_obs | hr*ug/L | 15903.6524 |
| 4 | AUCINF_D_obs | hr*kg*ug/L/mg | 7484.0717 |
| 4 | AUC_%Extrap_obs | % | 3.4653 |
| 4 | Vz_F_obs | L/kg | 6.5749 |
| 4 | Cl_F_obs | L/hr/kg | 0.1336 |
| 4 | AUCINF_pred | hr*ug/L | 15922.6960 |
| 4 | AUCINF_D_pred | hr*kg*ug/L/mg | 7493.0334 |
| 4 | AUC_%Extrap_pred | % | 3.5808 |
| 4 | Vz_F_pred | L/kg | 6.5670 |
| 4 | Cl_F_pred | L/hr/kg | 0.1335 |
| 4 | AUMClast | hr*hr*ug/L | 186896.7695 |
| 4 | AUMCINF_obs | hr*hr*ug/L | 266922.7234 |
| 4 | AUMC_%Extrap_obs | % | 29.9809 |
| 4 | AUMCINF_pred | hr*hr*ug/L | 269687.9838 |
| 4 | AUMC_%Extrap_pred | % | 30.6989 |
| 4 | MRTlast | hr | 12.1737 |
| 4 | MRTINF_obs | hr | 16.7837 |
| 4 | MRTINF_pred | hr | 16.9373 |
| 5 | Rsq |  | 0.9044 |
| 5 | Rsq_adjusted |  | 0.8805 |
| 5 | Corr_XY |  | -0.9510 |
| 5 | No_points_lambda_z |  | 6.0000 |
| 5 | Lambda_z | 1/hr | 0.0350 |
| 5 | Lambda_z_lower | hr | 12.0000 |
| 5 | Lambda_z_upper | hr | 96.0000 |
| 5 | HL_Lambda_z | hr | 19.8031 |
| 5 | Tlag | hr | 0.0000 |
| 5 | Tmax | hr | 2.0000 |
| 5 | Cmax | ug/L | 2100.0000 |
| 5 | Cmax_D | kg*ug/L/mg | 988.2353 |
| 5 | Tlast | hr | 96.0000 |
| 5 | Clast | ug/L | 9.6000 |
| 5 | AUClast | hr*ug/L | 16378.2872 |
| 5 | AUCall | hr*ug/L | 16378.2872 |
| 5 | AUCINF_obs | hr*ug/L | 16652.5571 |
| 5 | AUCINF_D_obs | hr*kg*ug/L/mg | 7836.4975 |
| 5 | AUC_%Extrap_obs | % | 1.6470 |
| 5 | Vz_F_obs | L/kg | 3.6457 |
| 5 | Cl_F_obs | L/hr/kg | 0.1276 |
| 5 | AUCINF_pred | hr*ug/L | 16624.2506 |
| 5 | AUCINF_D_pred | hr*kg*ug/L/mg | 7823.1768 |
| 5 | AUC_%Extrap_pred | % | 1.4795 |
| 5 | Vz_F_pred | L/kg | 3.6519 |
| 5 | Cl_F_pred | L/hr/kg | 0.1278 |
| 5 | AUMClast | hr*hr*ug/L | 204607.6292 |
| 5 | AUMCINF_obs | hr*hr*ug/L | 238773.3841 |
| 5 | AUMC_%Extrap_obs | % | 14.3089 |
| 5 | AUMCINF_pred | hr*hr*ug/L | 235247.2443 |
| 5 | AUMC_%Extrap_pred | % | 13.0244 |
| 5 | MRTlast | hr | 12.4926 |
| 5 | MRTINF_obs | hr | 14.3385 |
| 5 | MRTINF_pred | hr | 14.1508 |
| 6 | Rsq |  | 0.9968 |
| 6 | Rsq_adjusted |  | 0.9935 |
| 6 | Corr_XY |  | -0.9984 |
| 6 | No_points_lambda_z |  | 3.0000 |
| 6 | Lambda_z | 1/hr | 0.0331 |
| 6 | Lambda_z_lower | hr | 48.0000 |
| 6 | Lambda_z_upper | hr | 96.0000 |
| 6 | HL_Lambda_z | hr | 20.9225 |
| 6 | Tlag | hr | 0.0000 |
| 6 | Tmax | hr | 2.0000 |
| 6 | Cmax | ug/L | 2180.0000 |
| 6 | Cmax_D | kg*ug/L/mg | 1025.8824 |
| 6 | Tlast | hr | 96.0000 |
| 6 | Clast | ug/L | 8.4000 |
| 6 | AUClast | hr*ug/L | 16362.0844 |
| 6 | AUCall | hr*ug/L | 16362.0844 |
| 6 | AUCINF_obs | hr*ug/L | 16615.6364 |
| 6 | AUCINF_D_obs | hr*kg*ug/L/mg | 7819.1230 |
| 6 | AUC_%Extrap_obs | % | 1.5260 |
| 6 | Vz_F_obs | L/kg | 3.8604 |
| 6 | Cl_F_obs | L/hr/kg | 0.1279 |
| 6 | AUCINF_pred | hr*ug/L | 16609.0939 |
| 6 | AUCINF_D_pred | hr*kg*ug/L/mg | 7816.0442 |
| 6 | AUC_%Extrap_pred | % | 1.4872 |
| 6 | Vz_F_pred | L/kg | 3.8619 |
| 6 | Cl_F_pred | L/hr/kg | 0.1279 |
| 6 | AUMClast | hr*hr*ug/L | 186290.9771 |
| 6 | AUMCINF_obs | hr*hr*ug/L | 218285.3708 |
| 6 | AUMC_%Extrap_obs | % | 14.6571 |
| 6 | AUMCINF_pred | hr*hr*ug/L | 217459.8154 |
| 6 | AUMC_%Extrap_pred | % | 14.3331 |
| 6 | MRTlast | hr | 11.3855 |
| 6 | MRTINF_obs | hr | 13.1373 |
| 6 | MRTINF_pred | hr | 13.0928 |
| 7 | Rsq |  | 0.8190 |
| 7 | Rsq_adjusted |  | 0.7932 |
| 7 | Corr_XY |  | -0.9050 |
| 7 | No_points_lambda_z |  | 9.0000 |
| 7 | Lambda_z | 1/hr | 0.0426 |
| 7 | Lambda_z_lower | hr | 6.0000 |
| 7 | Lambda_z_upper | hr | 96.0000 |
| 7 | HL_Lambda_z | hr | 16.2601 |
| 7 | Tlag | hr | 0.0000 |
| 7 | Tmax | hr | 2.0000 |
| 7 | Cmax | ug/L | 2300.0000 |
| 7 | Cmax_D | kg*ug/L/mg | 1082.3529 |
| 7 | Tlast | hr | 96.0000 |
| 7 | Clast | ug/L | 15.4000 |
| 7 | AUClast | hr*ug/L | 13645.3390 |
| 7 | AUCall | hr*ug/L | 13645.3390 |
| 7 | AUCINF_obs | hr*ug/L | 14006.5986 |
| 7 | AUCINF_D_obs | hr*kg*ug/L/mg | 6591.3405 |
| 7 | AUC_%Extrap_obs | % | 2.5792 |
| 7 | Vz_F_obs | L/kg | 3.5590 |
| 7 | Cl_F_obs | L/hr/kg | 0.1517 |
| 7 | AUCINF_pred | hr*ug/L | 13800.4881 |
| 7 | AUCINF_D_pred | hr*kg*ug/L/mg | 6494.3473 |
| 7 | AUC_%Extrap_pred | % | 1.1242 |
| 7 | Vz_F_pred | L/kg | 3.6121 |
| 7 | Cl_F_pred | L/hr/kg | 0.1540 |
| 7 | AUMClast | hr*hr*ug/L | 162157.5046 |
| 7 | AUMCINF_obs | hr*hr*ug/L | 205313.0028 |
| 7 | AUMC_%Extrap_obs | % | 21.0194 |
| 7 | AUMCINF_pred | hr*hr*ug/L | 180691.3680 |
| 7 | AUMC_%Extrap_pred | % | 10.2572 |
| 7 | MRTlast | hr | 11.8837 |
| 7 | MRTINF_obs | hr | 14.6583 |
| 7 | MRTINF_pred | hr | 13.0931 |
| 8 | Rsq |  | 0.9867 |
| 8 | Rsq_adjusted |  | 0.9800 |
| 8 | Corr_XY |  | -0.9933 |
| 8 | No_points_lambda_z |  | 4.0000 |
| 8 | Lambda_z | 1/hr | 0.0197 |
| 8 | Lambda_z_lower | hr | 36.0000 |
| 8 | Lambda_z_upper | hr | 96.0000 |
| 8 | HL_Lambda_z | hr | 35.1911 |
| 8 | Tlag | hr | 0.0000 |
| 8 | Tmax | hr | 2.0000 |
| 8 | Cmax | ug/L | 2010.0000 |
| 8 | Cmax_D | kg*ug/L/mg | 945.8824 |
| 8 | Tlast | hr | 96.0000 |
| 8 | Clast | ug/L | 14.3000 |
| 8 | AUClast | hr*ug/L | 13876.5373 |
| 8 | AUCall | hr*ug/L | 13876.5373 |
| 8 | AUCINF_obs | hr*ug/L | 14602.5482 |
| 8 | AUCINF_D_obs | hr*kg*ug/L/mg | 6871.7874 |
| 8 | AUC_%Extrap_obs | % | 4.9718 |
| 8 | Vz_F_obs | L/kg | 7.3882 |
| 8 | Cl_F_obs | L/hr/kg | 0.1455 |
| 8 | AUCINF_pred | hr*ug/L | 14566.2128 |
| 8 | AUCINF_D_pred | hr*kg*ug/L/mg | 6854.6884 |
| 8 | AUC_%Extrap_pred | % | 4.7348 |
| 8 | Vz_F_pred | L/kg | 7.4066 |
| 8 | Cl_F_pred | L/hr/kg | 0.1459 |
| 8 | AUMClast | hr*hr*ug/L | 191623.8870 |
| 8 | AUMCINF_obs | hr*hr*ug/L | 298180.4995 |
| 8 | AUMC_%Extrap_obs | % | 35.7356 |
| 8 | AUMCINF_pred | hr*hr*ug/L | 292847.5565 |
| 8 | AUMC_%Extrap_pred | % | 34.5653 |
| 8 | MRTlast | hr | 13.8092 |
| 8 | MRTINF_obs | hr | 20.4198 |
| 8 | MRTINF_pred | hr | 20.1046 |
| 9 | Rsq |  | 0.7919 |
| 9 | Rsq_adjusted |  | 0.7659 |
| 9 | Corr_XY |  | -0.8899 |
| 9 | No_points_lambda_z |  | 10.0000 |
| 9 | Lambda_z | 1/hr | 0.0496 |
| 9 | Lambda_z_lower | hr | 4.0000 |
| 9 | Lambda_z_upper | hr | 96.0000 |
| 9 | HL_Lambda_z | hr | 13.9879 |
| 9 | Tlag | hr | 0.0000 |
| 9 | Tmax | hr | 2.0000 |
| 9 | Cmax | ug/L | 2190.0000 |
| 9 | Cmax_D | kg*ug/L/mg | 1030.5882 |
| 9 | Tlast | hr | 96.0000 |
| 9 | Clast | ug/L | 13.2000 |
| 9 | AUClast | hr*ug/L | 12790.0395 |
| 9 | AUCall | hr*ug/L | 12790.0395 |
| 9 | AUCINF_obs | hr*ug/L | 13056.4195 |
| 9 | AUCINF_D_obs | hr*kg*ug/L/mg | 6144.1974 |
| 9 | AUC_%Extrap_obs | % | 2.0402 |
| 9 | Vz_F_obs | L/kg | 3.2844 |
| 9 | Cl_F_obs | L/hr/kg | 0.1628 |
| 9 | AUCINF_pred | hr*ug/L | 12873.8883 |
| 9 | AUCINF_D_pred | hr*kg*ug/L/mg | 6058.3004 |
| 9 | AUC_%Extrap_pred | % | 0.6513 |
| 9 | Vz_F_pred | L/kg | 3.3310 |
| 9 | Cl_F_pred | L/hr/kg | 0.1651 |
| 9 | AUMClast | hr*hr*ug/L | 137623.0046 |
| 9 | AUMCINF_obs | hr*hr*ug/L | 168571.1215 |
| 9 | AUMC_%Extrap_obs | % | 18.3591 |
| 9 | AUMCINF_pred | hr*hr*ug/L | 147364.5854 |
| 9 | AUMC_%Extrap_pred | % | 6.6105 |
| 9 | MRTlast | hr | 10.7602 |
| 9 | MRTINF_obs | hr | 12.9110 |
| 9 | MRTINF_pred | hr | 11.4468 |
| 10 | Rsq |  | 0.9968 |
| 10 | Rsq_adjusted |  | 0.9937 |
| 10 | Corr_XY |  | -0.9984 |
| 10 | No_points_lambda_z |  | 3.0000 |
| 10 | Lambda_z | 1/hr | 0.0206 |
| 10 | Lambda_z_lower | hr | 48.0000 |
| 10 | Lambda_z_upper | hr | 96.0000 |
| 10 | HL_Lambda_z | hr | 33.6961 |
| 10 | Tlag | hr | 0.0000 |
| 10 | Tmax | hr | 2.0000 |
| 10 | Cmax | ug/L | 2440.0000 |
| 10 | Cmax_D | kg*ug/L/mg | 1148.2353 |
| 10 | Tlast | hr | 96.0000 |
| 10 | Clast | ug/L | 11.4000 |
| 10 | AUClast | hr*ug/L | 14512.0359 |
| 10 | AUCall | hr*ug/L | 14512.0359 |
| 10 | AUCINF_obs | hr*ug/L | 15066.2261 |
| 10 | AUCINF_D_obs | hr*kg*ug/L/mg | 7089.9887 |
| 10 | AUC_%Extrap_obs | % | 3.6784 |
| 10 | Vz_F_obs | L/kg | 6.8566 |
| 10 | Cl_F_obs | L/hr/kg | 0.1410 |
| 10 | AUCINF_pred | hr*ug/L | 15075.2063 |
| 10 | AUCINF_D_pred | hr*kg*ug/L/mg | 7094.2147 |
| 10 | AUC_%Extrap_pred | % | 3.7357 |
| 10 | Vz_F_pred | L/kg | 6.8525 |
| 10 | Cl_F_pred | L/hr/kg | 0.1410 |
| 10 | AUMClast | hr*hr*ug/L | 175525.0031 |
| 10 | AUMCINF_obs | hr*hr*ug/L | 255668.2063 |
| 10 | AUMC_%Extrap_obs | % | 31.3466 |
| 10 | AUMCINF_pred | hr*hr*ug/L | 256966.8613 |
| 10 | AUMC_%Extrap_pred | % | 31.6935 |
| 10 | MRTlast | hr | 12.0951 |
| 10 | MRTINF_obs | hr | 16.9696 |
| 10 | MRTINF_pred | hr | 17.0457 |
| 11 | Rsq |  | 0.9969 |
| 11 | Rsq_adjusted |  | 0.9954 |
| 11 | Corr_XY |  | -0.9985 |
| 11 | No_points_lambda_z |  | 4.0000 |
| 11 | Lambda_z | 1/hr | 0.0227 |
| 11 | Lambda_z_lower | hr | 36.0000 |
| 11 | Lambda_z_upper | hr | 96.0000 |
| 11 | HL_Lambda_z | hr | 30.5131 |
| 11 | Tlag | hr | 0.0000 |
| 11 | Tmax | hr | 2.0000 |
| 11 | Cmax | ug/L | 1950.0000 |
| 11 | Cmax_D | kg*ug/L/mg | 917.6471 |
| 11 | Tlast | hr | 96.0000 |
| 11 | Clast | ug/L | 9.3000 |
| 11 | AUClast | hr*ug/L | 11114.2843 |
| 11 | AUCall | hr*ug/L | 11114.2843 |
| 11 | AUCINF_obs | hr*ug/L | 11523.6808 |
| 11 | AUCINF_D_obs | hr*kg*ug/L/mg | 5422.9086 |
| 11 | AUC_%Extrap_obs | % | 3.5527 |
| 11 | Vz_F_obs | L/kg | 8.1176 |
| 11 | Cl_F_obs | L/hr/kg | 0.1844 |
| 11 | AUCINF_pred | hr*ug/L | 11519.6292 |
| 11 | AUCINF_D_pred | hr*kg*ug/L/mg | 5421.0020 |
| 11 | AUC_%Extrap_pred | % | 3.5187 |
| 11 | Vz_F_pred | L/kg | 8.1205 |
| 11 | Cl_F_pred | L/hr/kg | 0.1845 |
| 11 | AUMClast | hr*hr*ug/L | 136696.1942 |
| 11 | AUMCINF_obs | hr*hr*ug/L | 194020.3471 |
| 11 | AUMC_%Extrap_obs | % | 29.5454 |
| 11 | AUMCINF_pred | hr*hr*ug/L | 193453.0376 |
| 11 | AUMC_%Extrap_pred | % | 29.3388 |
| 11 | MRTlast | hr | 12.2991 |
| 11 | MRTINF_obs | hr | 16.8367 |
| 11 | MRTINF_pred | hr | 16.7933 |
| 12 | Rsq |  | 0.9900 |
| 12 | Rsq_adjusted |  | 0.9849 |
| 12 | Corr_XY |  | -0.9950 |
| 12 | No_points_lambda_z |  | 4.0000 |
| 12 | Lambda_z | 1/hr | 0.0225 |
| 12 | Lambda_z_lower | hr | 36.0000 |
| 12 | Lambda_z_upper | hr | 96.0000 |
| 12 | HL_Lambda_z | hr | 30.7397 |
| 12 | Tlag | hr | 0.0000 |
| 12 | Tmax | hr | 2.0000 |
| 12 | Cmax | ug/L | 2110.0000 |
| 12 | Cmax_D | kg*ug/L/mg | 992.9412 |
| 12 | Tlast | hr | 96.0000 |
| 12 | Clast | ug/L | 6.3000 |
| 12 | AUClast | hr*ug/L | 11254.7865 |
| 12 | AUCall | hr*ug/L | 11254.7865 |
| 12 | AUCINF_obs | hr*ug/L | 11534.1786 |
| 12 | AUCINF_D_obs | hr*kg*ug/L/mg | 5427.8488 |
| 12 | AUC_%Extrap_obs | % | 2.4223 |
| 12 | Vz_F_obs | L/kg | 8.1704 |
| 12 | Cl_F_obs | L/hr/kg | 0.1842 |
| 12 | AUCINF_pred | hr*ug/L | 11547.8337 |
| 12 | AUCINF_D_pred | hr*kg*ug/L/mg | 5434.2747 |
| 12 | AUC_%Extrap_pred | % | 2.5377 |
| 12 | Vz_F_pred | L/kg | 8.1608 |
| 12 | Cl_F_pred | L/hr/kg | 0.1840 |
| 12 | AUMClast | hr*hr*ug/L | 114182.9947 |
| 12 | AUMCINF_obs | hr*hr*ug/L | 153395.1198 |
| 12 | AUMC_%Extrap_obs | % | 25.5628 |
| 12 | AUMCINF_pred | hr*hr*ug/L | 155311.5786 |
| 12 | AUMC_%Extrap_pred | % | 26.4813 |
| 12 | MRTlast | hr | 10.1453 |
| 12 | MRTINF_obs | hr | 13.2992 |
| 12 | MRTINF_pred | hr | 13.4494 |
| 13 | Rsq |  | 0.8650 |
| 13 | Rsq_adjusted |  | 0.8481 |
| 13 | Corr_XY |  | -0.9300 |
| 13 | No_points_lambda_z |  | 10.0000 |
| 13 | Lambda_z | 1/hr | 0.0559 |
| 13 | Lambda_z_lower | hr | 4.0000 |
| 13 | Lambda_z_upper | hr | 96.0000 |
| 13 | HL_Lambda_z | hr | 12.4094 |
| 13 | Tlag | hr | 0.0000 |
| 13 | Tmax | hr | 2.0000 |
| 13 | Cmax | ug/L | 2070.0000 |
| 13 | Cmax_D | kg*ug/L/mg | 974.1176 |
| 13 | Tlast | hr | 96.0000 |
| 13 | Clast | ug/L | 6.0000 |
| 13 | AUClast | hr*ug/L | 12577.6837 |
| 13 | AUCall | hr*ug/L | 12577.6837 |
| 13 | AUCINF_obs | hr*ug/L | 12685.1011 |
| 13 | AUCINF_D_obs | hr*kg*ug/L/mg | 5969.4594 |
| 13 | AUC_%Extrap_obs | % | 0.8468 |
| 13 | Vz_F_obs | L/kg | 2.9991 |
| 13 | Cl_F_obs | L/hr/kg | 0.1675 |
| 13 | AUCINF_pred | hr*ug/L | 12620.2479 |
| 13 | AUCINF_D_pred | hr*kg*ug/L/mg | 5938.9402 |
| 13 | AUC_%Extrap_pred | % | 0.3373 |
| 13 | Vz_F_pred | L/kg | 3.0145 |
| 13 | Cl_F_pred | L/hr/kg | 0.1684 |
| 13 | AUMClast | hr*hr*ug/L | 126817.5595 |
| 13 | AUMCINF_obs | hr*hr*ug/L | 139052.7242 |
| 13 | AUMC_%Extrap_obs | % | 8.7989 |
| 13 | AUMCINF_pred | hr*hr*ug/L | 131665.7534 |
| 13 | AUMC_%Extrap_pred | % | 3.6822 |
| 13 | MRTlast | hr | 10.0827 |
| 13 | MRTINF_obs | hr | 10.9619 |
| 13 | MRTINF_pred | hr | 10.4329 |
| 14 | Rsq |  | 0.9995 |
| 14 | Rsq_adjusted |  | 0.9991 |
| 14 | Corr_XY |  | -0.9998 |
| 14 | No_points_lambda_z |  | 3.0000 |
| 14 | Lambda_z | 1/hr | 0.0143 |
| 14 | Lambda_z_lower | hr | 48.0000 |
| 14 | Lambda_z_upper | hr | 96.0000 |
| 14 | HL_Lambda_z | hr | 48.5494 |
| 14 | Tlag | hr | 0.0000 |
| 14 | Tmax | hr | 2.0000 |
| 14 | Cmax | ug/L | 1930.0000 |
| 14 | Cmax_D | kg*ug/L/mg | 908.2353 |
| 14 | Tlast | hr | 96.0000 |
| 14 | Clast | ug/L | 12.8000 |
| 14 | AUClast | hr*ug/L | 14776.4848 |
| 14 | AUCall | hr*ug/L | 14776.4848 |
| 14 | AUCINF_obs | hr*ug/L | 15673.0212 |
| 14 | AUCINF_D_obs | hr*kg*ug/L/mg | 7375.5394 |
| 14 | AUC_%Extrap_obs | % | 5.7203 |
| 14 | Vz_F_obs | L/kg | 9.4965 |
| 14 | Cl_F_obs | L/hr/kg | 0.1356 |
| 14 | AUCINF_pred | hr*ug/L | 15669.1748 |
| 14 | AUCINF_D_pred | hr*kg*ug/L/mg | 7373.7293 |
| 14 | AUC_%Extrap_pred | % | 5.6971 |
| 14 | Vz_F_pred | L/kg | 9.4988 |
| 14 | Cl_F_pred | L/hr/kg | 0.1356 |
| 14 | AUMClast | hr*hr*ug/L | 170447.2776 |
| 14 | AUMCINF_obs | hr*hr*ug/L | 319309.8960 |
| 14 | AUMC_%Extrap_obs | % | 46.6201 |
| 14 | AUMCINF_pred | hr*hr*ug/L | 318671.2245 |
| 14 | AUMC_%Extrap_pred | % | 46.5131 |
| 14 | MRTlast | hr | 11.5350 |
| 14 | MRTINF_obs | hr | 20.3732 |
| 14 | MRTINF_pred | hr | 20.3375 |
| 15 | Rsq |  | 0.8295 |
| 15 | Rsq_adjusted |  | 0.8052 |
| 15 | Corr_XY |  | -0.9108 |
| 15 | No_points_lambda_z |  | 9.0000 |
| 15 | Lambda_z | 1/hr | 0.0497 |
| 15 | Lambda_z_lower | hr | 6.0000 |
| 15 | Lambda_z_upper | hr | 96.0000 |
| 15 | HL_Lambda_z | hr | 13.9414 |
| 15 | Tlag | hr | 0.0000 |
| 15 | Tmax | hr | 2.0000 |
| 15 | Cmax | ug/L | 1930.0000 |
| 15 | Cmax_D | kg*ug/L/mg | 908.2353 |
| 15 | Tlast | hr | 96.0000 |
| 15 | Clast | ug/L | 6.6000 |
| 15 | AUClast | hr*ug/L | 12314.6365 |
| 15 | AUCall | hr*ug/L | 12314.6365 |
| 15 | AUCINF_obs | hr*ug/L | 12447.3837 |
| 15 | AUCINF_D_obs | hr*kg*ug/L/mg | 5857.5923 |
| 15 | AUC_%Extrap_obs | % | 1.0665 |
| 15 | Vz_F_obs | L/kg | 3.4337 |
| 15 | Cl_F_obs | L/hr/kg | 0.1707 |
| 15 | AUCINF_pred | hr*ug/L | 12376.5504 |
| 15 | AUCINF_D_pred | hr*kg*ug/L/mg | 5824.2590 |
| 15 | AUC_%Extrap_pred | % | 0.5003 |
| 15 | Vz_F_pred | L/kg | 3.4534 |
| 15 | Cl_F_pred | L/hr/kg | 0.1717 |
| 15 | AUMClast | hr*hr*ug/L | 119458.2367 |
| 15 | AUMCINF_obs | hr*hr*ug/L | 134871.9461 |
| 15 | AUMC_%Extrap_obs | % | 11.4284 |
| 15 | AUMCINF_pred | hr*hr*ug/L | 126647.2666 |
| 15 | AUMC_%Extrap_pred | % | 5.6764 |
| 15 | MRTlast | hr | 9.7005 |
| 15 | MRTINF_obs | hr | 10.8354 |
| 15 | MRTINF_pred | hr | 10.2328 |
| 16 | Rsq |  | 0.9092 |
| 16 | Rsq_adjusted |  | 0.8185 |
| 16 | Corr_XY |  | -0.9535 |
| 16 | No_points_lambda_z |  | 3.0000 |
| 16 | Lambda_z | 1/hr | 0.0122 |
| 16 | Lambda_z_lower | hr | 48.0000 |
| 16 | Lambda_z_upper | hr | 96.0000 |
| 16 | HL_Lambda_z | hr | 56.9463 |
| 16 | Tlag | hr | 0.0000 |
| 16 | Tmax | hr | 2.0000 |
| 16 | Cmax | ug/L | 2470.0000 |
| 16 | Cmax_D | kg*ug/L/mg | 1162.3529 |
| 16 | Tlast | hr | 96.0000 |
| 16 | Clast | ug/L | 12.6000 |
| 16 | AUClast | hr*ug/L | 12879.1899 |
| 16 | AUCall | hr*ug/L | 12879.1899 |
| 16 | AUCINF_obs | hr*ug/L | 13914.3577 |
| 16 | AUCINF_D_obs | hr*kg*ug/L/mg | 6547.9330 |
| 16 | AUC_%Extrap_obs | % | 7.4396 |
| 16 | Vz_F_obs | L/kg | 12.5469 |
| 16 | Cl_F_obs | L/hr/kg | 0.1527 |
| 16 | AUCINF_pred | hr*ug/L | 13971.0140 |
| 16 | AUCINF_D_pred | hr*kg*ug/L/mg | 6574.5948 |
| 16 | AUC_%Extrap_pred | % | 7.8149 |
| 16 | Vz_F_pred | L/kg | 12.4960 |
| 16 | Cl_F_pred | L/hr/kg | 0.1521 |
| 16 | AUMClast | hr*hr*ug/L | 140709.6964 |
| 16 | AUMCINF_obs | hr*hr*ug/L | 325131.2370 |
| 16 | AUMC_%Extrap_obs | % | 56.7222 |
| 16 | AUMCINF_pred | hr*hr*ug/L | 335224.8968 |
| 16 | AUMC_%Extrap_pred | % | 58.0253 |
| 16 | MRTlast | hr | 10.9254 |
| 16 | MRTINF_obs | hr | 23.3666 |
| 16 | MRTINF_pred | hr | 23.9943 |
| 17 | Rsq |  | 0.7907 |
| 17 | Rsq_adjusted |  | 0.7608 |
| 17 | Corr_XY |  | -0.8892 |
| 17 | No_points_lambda_z |  | 9.0000 |
| 17 | Lambda_z | 1/hr | 0.0433 |
| 17 | Lambda_z_lower | hr | 6.0000 |
| 17 | Lambda_z_upper | hr | 96.0000 |
| 17 | HL_Lambda_z | hr | 16.0095 |
| 17 | Tlag | hr | 0.0000 |
| 17 | Tmax | hr | 2.0000 |
| 17 | Cmax | ug/L | 2870.0000 |
| 17 | Cmax_D | kg*ug/L/mg | 1350.5882 |
| 17 | Tlast | hr | 96.0000 |
| 17 | Clast | ug/L | 12.0000 |
| 17 | AUClast | hr*ug/L | 14395.5868 |
| 17 | AUCall | hr*ug/L | 14395.5868 |
| 17 | AUCINF_obs | hr*ug/L | 14672.7494 |
| 17 | AUCINF_D_obs | hr*kg*ug/L/mg | 6904.8233 |
| 17 | AUC_%Extrap_obs | % | 1.8890 |
| 17 | Vz_F_obs | L/kg | 3.3450 |
| 17 | Cl_F_obs | L/hr/kg | 0.1448 |
| 17 | AUCINF_pred | hr*ug/L | 14524.1590 |
| 17 | AUCINF_D_pred | hr*kg*ug/L/mg | 6834.8984 |
| 17 | AUC_%Extrap_pred | % | 0.8852 |
| 17 | Vz_F_pred | L/kg | 3.3793 |
| 17 | Cl_F_pred | L/hr/kg | 0.1463 |
| 17 | AUMClast | hr*hr*ug/L | 151531.5783 |
| 17 | AUMCINF_obs | hr*hr*ug/L | 184540.7906 |
| 17 | AUMC_%Extrap_obs | % | 17.8872 |
| 17 | AUMCINF_pred | hr*hr*ug/L | 166844.1341 |
| 17 | AUMC_%Extrap_pred | % | 9.1778 |
| 17 | MRTlast | hr | 10.5263 |
| 17 | MRTINF_obs | hr | 12.5771 |
| 17 | MRTINF_pred | hr | 11.4874 |
| 18 | Rsq |  | 0.8410 |
| 18 | Rsq_adjusted |  | 0.8183 |
| 18 | Corr_XY |  | -0.9170 |
| 18 | No_points_lambda_z |  | 9.0000 |
| 18 | Lambda_z | 1/hr | 0.0515 |
| 18 | Lambda_z_lower | hr | 6.0000 |
| 18 | Lambda_z_upper | hr | 96.0000 |
| 18 | HL_Lambda_z | hr | 13.4497 |
| 18 | Tlag | hr | 0.0000 |
| 18 | Tmax | hr | 2.0000 |
| 18 | Cmax | ug/L | 2680.0000 |
| 18 | Cmax_D | kg*ug/L/mg | 1261.1765 |
| 18 | Tlast | hr | 96.0000 |
| 18 | Clast | ug/L | 8.3000 |
| 18 | AUClast | hr*ug/L | 17630.4873 |
| 18 | AUCall | hr*ug/L | 17630.4873 |
| 18 | AUCINF_obs | hr*ug/L | 17791.5395 |
| 18 | AUCINF_D_obs | hr*kg*ug/L/mg | 8372.4892 |
| 18 | AUC_%Extrap_obs | % | 0.9052 |
| 18 | Vz_F_obs | L/kg | 2.3176 |
| 18 | Cl_F_obs | L/hr/kg | 0.1194 |
| 18 | AUCINF_pred | hr*ug/L | 17700.5201 |
| 18 | AUCINF_D_pred | hr*kg*ug/L/mg | 8329.6565 |
| 18 | AUC_%Extrap_pred | % | 0.3957 |
| 18 | Vz_F_pred | L/kg | 2.3295 |
| 18 | Cl_F_pred | L/hr/kg | 0.1201 |
| 18 | AUMClast | hr*hr*ug/L | 163110.1788 |
| 18 | AUMCINF_obs | hr*hr*ug/L | 181696.2276 |
| 18 | AUMC_%Extrap_obs | % | 10.2292 |
| 18 | AUMCINF_pred | hr*hr*ug/L | 171192.2300 |
| 18 | AUMC_%Extrap_pred | % | 4.7210 |
| 18 | MRTlast | hr | 9.2516 |
| 18 | MRTINF_obs | hr | 10.2125 |
| 18 | MRTINF_pred | hr | 9.6716 |
| 19 | Rsq |  | 0.9044 |
| 19 | Rsq_adjusted |  | 0.8566 |
| 19 | Corr_XY |  | -0.9510 |
| 19 | No_points_lambda_z |  | 4.0000 |
| 19 | Lambda_z | 1/hr | 0.0152 |
| 19 | Lambda_z_lower | hr | 36.0000 |
| 19 | Lambda_z_upper | hr | 96.0000 |
| 19 | HL_Lambda_z | hr | 45.5009 |
| 19 | Tlag | hr | 0.0000 |
| 19 | Tmax | hr | 2.0000 |
| 19 | Cmax | ug/L | 2870.0000 |
| 19 | Cmax_D | kg*ug/L/mg | 1350.5882 |
| 19 | Tlast | hr | 96.0000 |
| 19 | Clast | ug/L | 14.8000 |
| 19 | AUClast | hr*ug/L | 16770.7357 |
| 19 | AUCall | hr*ug/L | 16770.7357 |
| 19 | AUCINF_obs | hr*ug/L | 17742.2648 |
| 19 | AUCINF_D_obs | hr*kg*ug/L/mg | 8349.3011 |
| 19 | AUC_%Extrap_obs | % | 5.4758 |
| 19 | Vz_F_obs | L/kg | 7.8622 |
| 19 | Cl_F_obs | L/hr/kg | 0.1198 |
| 19 | AUCINF_pred | hr*ug/L | 17734.5740 |
| 19 | AUCINF_D_pred | hr*kg*ug/L/mg | 8345.6819 |
| 19 | AUC_%Extrap_pred | % | 5.4348 |
| 19 | Vz_F_pred | L/kg | 7.8656 |
| 19 | Cl_F_pred | L/hr/kg | 0.1198 |
| 19 | AUMClast | hr*hr*ug/L | 193875.5022 |
| 19 | AUMCINF_obs | hr*hr*ug/L | 350917.2260 |
| 19 | AUMC_%Extrap_obs | % | 44.7518 |
| 19 | AUMCINF_pred | hr*hr*ug/L | 349674.0626 |
| 19 | AUMC_%Extrap_pred | % | 44.5554 |
| 19 | MRTlast | hr | 11.5603 |
| 19 | MRTINF_obs | hr | 19.7786 |
| 19 | MRTINF_pred | hr | 19.7171 |
| 20 | Rsq |  | 0.7820 |
| 20 | Rsq_adjusted |  | 0.7578 |
| 20 | Corr_XY |  | -0.8843 |
| 20 | No_points_lambda_z |  | 11.0000 |
| 20 | Lambda_z | 1/hr | 0.0528 |
| 20 | Lambda_z_lower | hr | 2.0000 |
| 20 | Lambda_z_upper | hr | 96.0000 |
| 20 | HL_Lambda_z | hr | 13.1169 |
| 20 | Tlag | hr | 0.0000 |
| 20 | Tmax | hr | 2.0000 |
| 20 | Cmax | ug/L | 2210.0000 |
| 20 | Cmax_D | kg*ug/L/mg | 1040.0000 |
| 20 | Tlast | hr | 96.0000 |
| 20 | Clast | ug/L | 13.8000 |
| 20 | AUClast | hr*ug/L | 18034.9870 |
| 20 | AUCall | hr*ug/L | 18034.9870 |
| 20 | AUCINF_obs | hr*ug/L | 18296.1348 |
| 20 | AUCINF_D_obs | hr*kg*ug/L/mg | 8609.9458 |
| 20 | AUC_%Extrap_obs | % | 1.4273 |
| 20 | Vz_F_obs | L/kg | 2.1979 |
| 20 | Cl_F_obs | L/hr/kg | 0.1161 |
| 20 | AUCINF_pred | hr*ug/L | 18137.3470 |
| 20 | AUCINF_D_pred | hr*kg*ug/L/mg | 8535.2221 |
| 20 | AUC_%Extrap_pred | % | 0.5644 |
| 20 | Vz_F_pred | L/kg | 2.2171 |
| 20 | Cl_F_pred | L/hr/kg | 0.1172 |
| 20 | AUMClast | hr*hr*ug/L | 212750.5453 |
| 20 | AUMCINF_obs | hr*hr*ug/L | 242762.6309 |
| 20 | AUMC_%Extrap_obs | % | 12.3627 |
| 20 | AUMCINF_pred | hr*hr*ug/L | 224514.1415 |
| 20 | AUMC_%Extrap_pred | % | 5.2396 |
| 20 | MRTlast | hr | 11.7965 |
| 20 | MRTINF_obs | hr | 13.2685 |
| 20 | MRTINF_pred | hr | 12.3786 |
| 21 | Rsq |  | 0.7630 |
| 21 | Rsq_adjusted |  | 0.7366 |
| 21 | Corr_XY |  | -0.8735 |
| 21 | No_points_lambda_z |  | 11.0000 |
| 21 | Lambda_z | 1/hr | 0.0562 |
| 21 | Lambda_z_lower | hr | 2.0000 |
| 21 | Lambda_z_upper | hr | 96.0000 |
| 21 | HL_Lambda_z | hr | 12.3382 |
| 21 | Tlag | hr | 0.0000 |
| 21 | Tmax | hr | 2.0000 |
| 21 | Cmax | ug/L | 2070.0000 |
| 21 | Cmax_D | kg*ug/L/mg | 974.1176 |
| 21 | Tlast | hr | 96.0000 |
| 21 | Clast | ug/L | 13.4000 |
| 21 | AUClast | hr*ug/L | 16727.9864 |
| 21 | AUCall | hr*ug/L | 16727.9864 |
| 21 | AUCINF_obs | hr*ug/L | 16966.5102 |
| 21 | AUCINF_D_obs | hr*kg*ug/L/mg | 7984.2401 |
| 21 | AUC_%Extrap_obs | % | 1.4059 |
| 21 | Vz_F_obs | L/kg | 2.2294 |
| 21 | Cl_F_obs | L/hr/kg | 0.1252 |
| 21 | AUCINF_pred | hr*ug/L | 16792.7263 |
| 21 | AUCINF_D_pred | hr*kg*ug/L/mg | 7902.4594 |
| 21 | AUC_%Extrap_pred | % | 0.3855 |
| 21 | Vz_F_pred | L/kg | 2.2525 |
| 21 | Cl_F_pred | L/hr/kg | 0.1265 |
| 21 | AUMClast | hr*hr*ug/L | 168620.0612 |
| 21 | AUMCINF_obs | hr*hr*ug/L | 195764.1420 |
| 21 | AUMC_%Extrap_obs | % | 13.8657 |
| 21 | AUMCINF_pred | hr*hr*ug/L | 175987.4815 |
| 21 | AUMC_%Extrap_pred | % | 4.1863 |
| 21 | MRTlast | hr | 10.0801 |
| 21 | MRTINF_obs | hr | 11.5383 |
| 21 | MRTINF_pred | hr | 10.4800 |
| 22 | Rsq |  | 0.9881 |
| 22 | Rsq_adjusted |  | 0.9762 |
| 22 | Corr_XY |  | -0.9940 |
| 22 | No_points_lambda_z |  | 3.0000 |
| 22 | Lambda_z | 1/hr | 0.0052 |
| 22 | Lambda_z_lower | hr | 48.0000 |
| 22 | Lambda_z_upper | hr | 96.0000 |
| 22 | HL_Lambda_z | hr | 132.3882 |
| 22 | Tlag | hr | 0.0000 |
| 22 | Tmax | hr | 1.0000 |
| 22 | Cmax | ug/L | 1930.0000 |
| 22 | Cmax_D | kg*ug/L/mg | 908.2353 |
| 22 | Tlast | hr | 96.0000 |
| 22 | Clast | ug/L | 11.2000 |
| 22 | AUClast | hr*ug/L | 12461.0380 |
| 22 | AUCall | hr*ug/L | 12461.0380 |
| 22 | AUCINF_obs | hr*ug/L | 14600.1909 |
| 22 | AUCINF_D_obs | hr*kg*ug/L/mg | 6870.6781 |
| 22 | AUC_%Extrap_obs | % | 14.6515 |
| 22 | Vz_F_obs | L/kg | 27.7987 |
| 22 | Cl_F_obs | L/hr/kg | 0.1455 |
| 22 | AUCINF_pred | hr*ug/L | 14583.2347 |
| 22 | AUCINF_D_pred | hr*kg*ug/L/mg | 6862.6987 |
| 22 | AUC_%Extrap_pred | % | 14.5523 |
| 22 | Vz_F_pred | L/kg | 27.8310 |
| 22 | Cl_F_pred | L/hr/kg | 0.1457 |
| 22 | AUMClast | hr*hr*ug/L | 120022.2705 |
| 22 | AUMCINF_obs | hr*hr*ug/L | 733950.1796 |
| 22 | AUMC_%Extrap_obs | % | 83.6471 |
| 22 | AUMCINF_pred | hr*hr*ug/L | 729083.8243 |
| 22 | AUMC_%Extrap_pred | % | 83.5379 |
| 22 | MRTlast | hr | 9.6318 |
| 22 | MRTINF_obs | hr | 50.2699 |
| 22 | MRTINF_pred | hr | 49.9947 |
| 23 | Rsq |  | 0.8018 |
| 23 | Rsq_adjusted |  | 0.7735 |
| 23 | Corr_XY |  | -0.8955 |
| 23 | No_points_lambda_z |  | 9.0000 |
| 23 | Lambda_z | 1/hr | 0.0431 |
| 23 | Lambda_z_lower | hr | 6.0000 |
| 23 | Lambda_z_upper | hr | 96.0000 |
| 23 | HL_Lambda_z | hr | 16.0987 |
| 23 | Tlag | hr | 0.0000 |
| 23 | Tmax | hr | 2.0000 |
| 23 | Cmax | ug/L | 2140.0000 |
| 23 | Cmax_D | kg*ug/L/mg | 1007.0588 |
| 23 | Tlast | hr | 96.0000 |
| 23 | Clast | ug/L | 9.6000 |
| 23 | AUClast | hr*ug/L | 13224.8850 |
| 23 | AUCall | hr*ug/L | 13224.8850 |
| 23 | AUCINF_obs | hr*ug/L | 13447.8495 |
| 23 | AUCINF_D_obs | hr*kg*ug/L/mg | 6328.3998 |
| 23 | AUC_%Extrap_obs | % | 1.6580 |
| 23 | Vz_F_obs | L/kg | 3.6700 |
| 23 | Cl_F_obs | L/hr/kg | 0.1580 |
| 23 | AUCINF_pred | hr*ug/L | 13353.8208 |
| 23 | AUCINF_D_pred | hr*kg*ug/L/mg | 6284.1510 |
| 23 | AUC_%Extrap_pred | % | 0.9655 |
| 23 | Vz_F_pred | L/kg | 3.6959 |
| 23 | Cl_F_pred | L/hr/kg | 0.1591 |
| 23 | AUMClast | hr*hr*ug/L | 152043.0110 |
| 23 | AUMCINF_obs | hr*hr*ug/L | 178626.0648 |
| 23 | AUMC_%Extrap_obs | % | 14.8820 |
| 23 | AUMCINF_pred | hr*hr*ug/L | 167415.4417 |
| 23 | AUMC_%Extrap_pred | % | 9.1822 |
| 23 | MRTlast | hr | 11.4967 |
| 23 | MRTINF_obs | hr | 13.2829 |
| 23 | MRTINF_pred | hr | 12.5369 |
| 24 | Rsq |  | 0.9978 |
| 24 | Rsq_adjusted |  | 0.9967 |
| 24 | Corr_XY |  | -0.9989 |
| 24 | No_points_lambda_z |  | 4.0000 |
| 24 | Lambda_z | 1/hr | 0.0271 |
| 24 | Lambda_z_lower | hr | 36.0000 |
| 24 | Lambda_z_upper | hr | 96.0000 |
| 24 | HL_Lambda_z | hr | 25.5468 |
| 24 | Tlag | hr | 0.0000 |
| 24 | Tmax | hr | 2.0000 |
| 24 | Cmax | ug/L | 2250.0000 |
| 24 | Cmax_D | kg*ug/L/mg | 1058.8235 |
| 24 | Tlast | hr | 96.0000 |
| 24 | Clast | ug/L | 6.9000 |
| 24 | AUClast | hr*ug/L | 15753.9869 |
| 24 | AUCall | hr*ug/L | 15753.9869 |
| 24 | AUCINF_obs | hr*ug/L | 16008.2949 |
| 24 | AUCINF_D_obs | hr*kg*ug/L/mg | 7533.3153 |
| 24 | AUC_%Extrap_obs | % | 1.5886 |
| 24 | Vz_F_obs | L/kg | 4.8924 |
| 24 | Cl_F_obs | L/hr/kg | 0.1327 |
| 24 | AUCINF_pred | hr*ug/L | 16015.4904 |
| 24 | AUCINF_D_pred | hr*kg*ug/L/mg | 7536.7013 |
| 24 | AUC_%Extrap_pred | % | 1.6328 |
| 24 | Vz_F_pred | L/kg | 4.8902 |
| 24 | Cl_F_pred | L/hr/kg | 0.1327 |
| 24 | AUMClast | hr*hr*ug/L | 165265.0116 |
| 24 | AUMCINF_obs | hr*hr*ug/L | 199051.4226 |
| 24 | AUMC_%Extrap_obs | % | 16.9737 |
| 24 | AUMCINF_pred | hr*hr*ug/L | 200007.3768 |
| 24 | AUMC_%Extrap_pred | % | 17.3705 |
| 24 | MRTlast | hr | 10.4904 |
| 24 | MRTINF_obs | hr | 12.4343 |
| 24 | MRTINF_pred | hr | 12.4884 |

Worksheet: Non-Transposed Final Parameters

| **Animal** | **Constant** | **Value** |
| --- | --- | --- |
| 1 | Dose | 2.125 |
| 1 | Time of Last Dose | 0 |
| 2 | Dose | 2.125 |
| 2 | Time of Last Dose | 0 |
| 3 | Dose | 2.125 |
| 3 | Time of Last Dose | 0 |
| 4 | Dose | 2.125 |
| 4 | Time of Last Dose | 0 |
| 5 | Dose | 2.125 |
| 5 | Time of Last Dose | 0 |
| 6 | Dose | 2.125 |
| 6 | Time of Last Dose | 0 |
| 7 | Dose | 2.125 |
| 7 | Time of Last Dose | 0 |
| 8 | Dose | 2.125 |
| 8 | Time of Last Dose | 0 |
| 9 | Dose | 2.125 |
| 9 | Time of Last Dose | 0 |
| 10 | Dose | 2.125 |
| 10 | Time of Last Dose | 0 |
| 11 | Dose | 2.125 |
| 11 | Time of Last Dose | 0 |
| 12 | Dose | 2.125 |
| 12 | Time of Last Dose | 0 |
| 13 | Dose | 2.125 |
| 13 | Time of Last Dose | 0 |
| 14 | Dose | 2.125 |
| 14 | Time of Last Dose | 0 |
| 15 | Dose | 2.125 |
| 15 | Time of Last Dose | 0 |
| 16 | Dose | 2.125 |
| 16 | Time of Last Dose | 0 |
| 17 | Dose | 2.125 |
| 17 | Time of Last Dose | 0 |
| 18 | Dose | 2.125 |
| 18 | Time of Last Dose | 0 |
| 19 | Dose | 2.125 |
| 19 | Time of Last Dose | 0 |
| 20 | Dose | 2.125 |
| 20 | Time of Last Dose | 0 |
| 21 | Dose | 2.125 |
| 21 | Time of Last Dose | 0 |
| 22 | Dose | 2.125 |
| 22 | Time of Last Dose | 0 |
| 23 | Dose | 2.125 |
| 23 | Time of Last Dose | 0 |
| 24 | Dose | 2.125 |
| 24 | Time of Last Dose | 0 |

Worksheet: Dosing

| **Animal** | **Time (hr)** | **lambda_z_Incl** | **Concentration (ug/L)** | **Predicted (ug/L)** | **Residual (ug/L)** | **AUC (hr*ug/L)** | **AUMC (hr*hr*ug/L)** | **Weight** |
| --- | --- | --- | --- | --- | --- | --- | --- | --- |
| 1 | 0.0000 |  | 0.0000 |  |  | 0.0000 | 0.0000 | 0.0000 |
| 1 | 0.1667 |  | 158.0000 |  |  | 13.1693 | 2.1953 | 0.0000 |
| 1 | 0.5000 |  | 753.0000 |  |  | 164.9875 | 69.3284 | 0.0000 |
| 1 | 1.0000 |  | 1260.0000 |  |  | 668.2375 | 478.4534 | 0.0000 |
| 1 | 2.0000 |  | 2550.0000 |  |  | 2573.2375 | 3658.4534 | 0.0000 |
| 1 | 4.0000 |  | 1550.0000 |  |  | 6673.2375 | 14958.4534 | 0.0000 |
| 1 | 6.0000 |  | 816.0000 |  |  | 9039.2375 | 26054.4534 | 0.0000 |
| 1 | 8.0000 |  | 924.0000 |  |  | 10779.2375 | 38342.4534 | 0.0000 |
| 1 | 10.0000 |  | 432.0000 |  |  | 12135.2375 | 50054.4534 | 0.0000 |
| 1 | 12.0000 |  | 242.0000 |  |  | 12809.2375 | 57278.4534 | 0.0000 |
| 1 | 24.0000 |  | 99.2000 |  |  | 14856.4375 | 88987.2534 | 0.0000 |
| 1 | 36.0000 |  | 33.8000 |  |  | 15654.4375 | 110572.8534 | 0.0000 |
| 1 | 48.0000 | * | 30.2000 | 30.2252 | -0.0252 | 16038.4375 | 126571.2534 | 1.0000 |
| 1 | 72.0000 | * | 19.4000 | 19.3676 | 0.0324 | 16633.6375 | 160728.0534 | 1.0000 |
| 1 | 96.0000 | * | 12.4000 | 12.4104 | -0.0104 | 17015.2375 | 191774.4534 | 1.0000 |
| 2 | 0.0000 |  | 0.0000 |  |  | 0.0000 | 0.0000 | 0.0000 |
| 2 | 0.1667 |  | 210.0000 |  |  | 17.5035 | 2.9178 | 0.0000 |
| 2 | 0.5000 |  | 693.0000 |  |  | 167.9885 | 66.4960 | 0.0000 |
| 2 | 1.0000 |  | 1540.0000 |  |  | 726.2385 | 538.1210 | 0.0000 |
| 2 | 2.0000 |  | 2530.0000 |  |  | 2761.2385 | 3838.1210 | 0.0000 |
| 2 | 4.0000 |  | 1030.0000 |  |  | 6321.2385 | 13018.1210 | 0.0000 |
| 2 | 6.0000 |  | 726.0000 |  |  | 8077.2385 | 21494.1210 | 0.0000 |
| 2 | 8.0000 |  | 1084.0000 |  |  | 9887.2385 | 34522.1210 | 0.0000 |
| 2 | 10.0000 |  | 262.0000 |  |  | 11233.2385 | 45814.1210 | 0.0000 |
| 2 | 12.0000 |  | 198.0000 |  |  | 11693.2385 | 50810.1210 | 0.0000 |
| 2 | 24.0000 |  | 66.4000 |  |  | 13279.6385 | 74627.7210 | 0.0000 |
| 2 | 36.0000 | * | 28.4000 | 27.5501 | 0.8499 | 13848.4385 | 90323.7210 | 1.0000 |
| 2 | 48.0000 | * | 25.6000 | 23.9881 | 1.6119 | 14172.4385 | 103830.9210 | 1.0000 |
| 2 | 72.0000 | * | 14.8000 | 18.1861 | -3.3861 | 14657.2385 | 131363.7210 | 1.0000 |
| 2 | 96.0000 | * | 15.4000 | 13.7874 | 1.6126 | 15019.6385 | 161891.7210 | 1.0000 |
| 3 | 0.0000 |  | 0.0000 |  |  | 0.0000 | 0.0000 | 0.0000 |
| 3 | 0.1667 |  | 171.0000 |  |  | 14.2529 | 2.3760 | 0.0000 |
| 3 | 0.5000 |  | 684.0000 |  |  | 156.7386 | 64.1207 | 0.0000 |
| 3 | 1.0000 |  | 1360.0000 |  |  | 667.7386 | 489.6207 | 0.0000 |
| 3 | 2.0000 |  | 2190.0000 |  |  | 2442.7386 | 3359.6207 | 0.0000 |
| 3 | 4.0000 |  | 1200.0000 |  |  | 5832.7386 | 12539.6207 | 0.0000 |
| 3 | 6.0000 |  | 798.0000 |  |  | 7830.7386 | 22127.6207 | 0.0000 |
| 3 | 8.0000 |  | 832.0000 |  |  | 9460.7386 | 33571.6207 | 0.0000 |
| 3 | 10.0000 |  | 292.0000 |  |  | 10584.7386 | 43147.6207 | 0.0000 |
| 3 | 12.0000 |  | 200.0000 |  |  | 11076.7386 | 48467.6207 | 0.0000 |
| 3 | 24.0000 | * | 63.6000 | 54.6975 | 8.9025 | 12658.3386 | 72026.0207 | 1.0000 |
| 3 | 36.0000 | * | 31.4000 | 38.4537 | -7.0537 | 13228.3386 | 87966.8207 | 1.0000 |
| 3 | 48.0000 | * | 23.8000 | 27.0340 | -3.2340 | 13559.5386 | 101603.6207 | 1.0000 |
| 3 | 72.0000 | * | 18.2000 | 13.3614 | 4.8386 | 14063.5386 | 131037.2207 | 1.0000 |
| 3 | 96.0000 | * | 5.8000 | 6.6038 | -0.8038 | 14351.5386 | 153443.6207 | 1.0000 |
| 4 | 0.0000 |  | 0.0000 |  |  | 0.0000 | 0.0000 | 0.0000 |
| 4 | 0.1667 |  | 210.0000 |  |  | 17.5035 | 2.9178 | 0.0000 |
| 4 | 0.5000 |  | 867.0000 |  |  | 196.9856 | 80.9945 | 0.0000 |
| 4 | 1.0000 |  | 1620.0000 |  |  | 818.7356 | 594.3695 | 0.0000 |
| 4 | 2.0000 |  | 2210.0000 |  |  | 2733.7356 | 3614.3695 | 0.0000 |
| 4 | 4.0000 |  | 1040.0000 |  |  | 5983.7356 | 12194.3695 | 0.0000 |
| 4 | 6.0000 |  | 972.0000 |  |  | 7995.7356 | 22186.3695 | 0.0000 |
| 4 | 8.0000 |  | 500.0000 |  |  | 9467.7356 | 32018.3695 | 0.0000 |
| 4 | 10.0000 |  | 294.0000 |  |  | 10261.7356 | 38958.3695 | 0.0000 |
| 4 | 12.0000 |  | 256.0000 |  |  | 10811.7356 | 44970.3695 | 0.0000 |
| 4 | 24.0000 |  | 115.2000 |  |  | 13038.9356 | 79991.1695 | 0.0000 |
| 4 | 36.0000 | * | 40.8000 | 39.2216 | 1.5784 | 13974.9356 | 105392.7695 | 1.0000 |
| 4 | 48.0000 | * | 28.0000 | 30.7337 | -2.7337 | 14387.7356 | 122269.5695 | 1.0000 |
| 4 | 72.0000 | * | 20.6000 | 18.8709 | 1.7291 | 14970.9356 | 156195.9695 | 1.0000 |
| 4 | 96.0000 | * | 11.2000 | 11.5870 | -0.3870 | 15352.5356 | 186896.7695 | 1.0000 |
| 5 | 0.0000 |  | 0.0000 |  |  | 0.0000 | 0.0000 | 0.0000 |
| 5 | 0.1667 |  | 269.0000 |  |  | 22.4212 | 3.7376 | 0.0000 |
| 5 | 0.5000 |  | 771.0000 |  |  | 195.7372 | 75.4542 | 0.0000 |
| 5 | 1.0000 |  | 1540.0000 |  |  | 773.4872 | 556.8292 | 0.0000 |
| 5 | 2.0000 |  | 2100.0000 |  |  | 2593.4872 | 3426.8292 | 0.0000 |
| 5 | 4.0000 |  | 1330.0000 |  |  | 6023.4872 | 12946.8292 | 0.0000 |
| 5 | 6.0000 |  | 966.0000 |  |  | 8319.4872 | 24062.8292 | 0.0000 |
| 5 | 8.0000 |  | 672.0000 |  |  | 9957.4872 | 35234.8292 | 0.0000 |
| 5 | 10.0000 |  | 432.0000 |  |  | 11061.4872 | 44930.8292 | 0.0000 |
| 5 | 12.0000 | * | 236.0000 | 162.8780 | 73.1220 | 11729.4872 | 52082.8292 | 1.0000 |
| 5 | 24.0000 | * | 111.2000 | 107.0159 | 4.1841 | 13812.6872 | 85087.6292 | 1.0000 |
| 5 | 36.0000 | * | 58.0000 | 70.3128 | -12.3128 | 14827.8872 | 113628.4292 | 1.0000 |
| 5 | 48.0000 | * | 25.2000 | 46.1977 | -20.9977 | 15327.0872 | 133414.0292 | 1.0000 |
| 5 | 72.0000 | * | 26.4000 | 19.9431 | 6.4569 | 15946.2872 | 170738.8292 | 1.0000 |
| 5 | 96.0000 | * | 9.6000 | 8.6092 | 0.9908 | 16378.2872 | 204607.6292 | 1.0000 |
| 6 | 0.0000 |  | 0.0000 |  |  | 0.0000 | 0.0000 | 0.0000 |
| 6 | 0.1667 |  | 182.0000 |  |  | 15.1697 | 2.5288 | 0.0000 |
| 6 | 0.5000 |  | 936.0000 |  |  | 201.4844 | 85.5771 | 0.0000 |
| 6 | 1.0000 |  | 1240.0000 |  |  | 745.4844 | 512.5771 | 0.0000 |
| 6 | 2.0000 |  | 2180.0000 |  |  | 2455.4844 | 3312.5771 | 0.0000 |
| 6 | 4.0000 |  | 1550.0000 |  |  | 6185.4844 | 13872.5771 | 0.0000 |
| 6 | 6.0000 |  | 924.0000 |  |  | 8659.4844 | 25616.5771 | 0.0000 |
| 6 | 8.0000 |  | 564.0000 |  |  | 10147.4844 | 35672.5771 | 0.0000 |
| 6 | 10.0000 |  | 290.0000 |  |  | 11001.4844 | 43084.5771 | 0.0000 |
| 6 | 12.0000 |  | 363.0000 |  |  | 11654.4844 | 50340.5771 | 0.0000 |
| 6 | 24.0000 |  | 76.8000 |  |  | 14293.2844 | 87535.7771 | 0.0000 |
| 6 | 36.0000 |  | 29.4000 |  |  | 14930.4844 | 104945.3771 | 0.0000 |
| 6 | 48.0000 | * | 41.2000 | 40.1369 | 1.0631 | 15354.0844 | 123161.3771 | 1.0000 |
| 6 | 72.0000 | * | 17.2000 | 18.1232 | -0.9232 | 16054.8844 | 161753.3771 | 1.0000 |
| 6 | 96.0000 | * | 8.4000 | 8.1833 | 0.2167 | 16362.0844 | 186290.9771 | 1.0000 |
| 7 | 0.0000 |  | 0.0000 |  |  | 0.0000 | 0.0000 | 0.0000 |
| 7 | 0.1667 |  | 209.0000 |  |  | 17.4202 | 2.9039 | 0.0000 |
| 7 | 0.5000 |  | 660.0000 |  |  | 162.2390 | 63.7046 | 0.0000 |
| 7 | 1.0000 |  | 1230.0000 |  |  | 634.7390 | 453.7046 | 0.0000 |
| 7 | 2.0000 |  | 2300.0000 |  |  | 2399.7390 | 3368.7046 | 0.0000 |
| 7 | 4.0000 |  | 1030.0000 |  |  | 5729.7390 | 12088.7046 | 0.0000 |
| 7 | 6.0000 | * | 654.0000 | 306.6581 | 347.3419 | 7413.7390 | 20132.7046 | 1.0000 |
| 7 | 8.0000 | * | 440.0000 | 281.5968 | 158.4032 | 8507.7390 | 27576.7046 | 1.0000 |
| 7 | 10.0000 | * | 368.0000 | 258.5836 | 109.4164 | 9315.7390 | 34776.7046 | 1.0000 |
| 7 | 12.0000 | * | 226.0000 | 237.4511 | -11.4511 | 9909.7390 | 41168.7046 | 1.0000 |
| 7 | 24.0000 | * | 76.4000 | 142.3684 | -65.9684 | 11724.1390 | 68442.3046 | 1.0000 |
| 7 | 36.0000 | * | 33.2000 | 85.3598 | -52.1598 | 12381.7390 | 86615.1046 | 1.0000 |
| 7 | 48.0000 | * | 29.4000 | 51.1791 | -21.7791 | 12757.3390 | 102253.5046 | 1.0000 |
| 7 | 72.0000 | * | 14.6000 | 18.3980 | -3.7980 | 13285.3390 | 131802.3046 | 1.0000 |
| 7 | 96.0000 | * | 15.4000 | 6.6138 | 8.7862 | 13645.3390 | 162157.5046 | 1.0000 |
| 8 | 0.0000 |  | 0.0000 |  |  | 0.0000 | 0.0000 | 0.0000 |
| 8 | 0.1667 |  | 205.0000 |  |  | 17.0868 | 2.8484 | 0.0000 |
| 8 | 0.5000 |  | 762.0000 |  |  | 178.2373 | 72.0370 | 0.0000 |
| 8 | 1.0000 |  | 1660.0000 |  |  | 783.7373 | 582.2870 | 0.0000 |
| 8 | 2.0000 |  | 2010.0000 |  |  | 2618.7373 | 3422.2870 | 0.0000 |
| 8 | 4.0000 |  | 570.0000 |  |  | 5198.7373 | 9722.2870 | 0.0000 |
| 8 | 6.0000 |  | 618.0000 |  |  | 6386.7373 | 15710.2870 | 0.0000 |
| 8 | 8.0000 |  | 704.0000 |  |  | 7708.7373 | 25050.2870 | 0.0000 |
| 8 | 10.0000 |  | 276.0000 |  |  | 8688.7373 | 33442.2870 | 0.0000 |
| 8 | 12.0000 |  | 233.0000 |  |  | 9197.7373 | 38998.2870 | 0.0000 |
| 8 | 24.0000 |  | 120.8000 |  |  | 11320.5373 | 73169.4870 | 0.0000 |
| 8 | 36.0000 | * | 45.8000 | 44.2881 | 1.5119 | 12320.1373 | 100457.4870 | 1.0000 |
| 8 | 48.0000 | * | 35.0000 | 34.9653 | 0.0347 | 12804.9373 | 120430.2870 | 1.0000 |
| 8 | 72.0000 | * | 20.0000 | 21.7940 | -1.7940 | 13464.9373 | 157870.2870 | 1.0000 |
| 8 | 96.0000 | * | 14.3000 | 13.5843 | 0.7157 | 13876.5373 | 191623.8870 | 1.0000 |
| 9 | 0.0000 |  | 0.0000 |  |  | 0.0000 | 0.0000 | 0.0000 |
| 9 | 0.1667 |  | 188.0000 |  |  | 15.6698 | 2.6122 | 0.0000 |
| 9 | 0.5000 |  | 633.0000 |  |  | 152.4895 | 60.5796 | 0.0000 |
| 9 | 1.0000 |  | 1110.0000 |  |  | 588.2395 | 417.2046 | 0.0000 |
| 9 | 2.0000 |  | 2190.0000 |  |  | 2238.2395 | 3162.2046 | 0.0000 |
| 9 | 4.0000 | * | 690.0000 | 396.7109 | 293.2891 | 5118.2395 | 10302.2046 | 1.0000 |
| 9 | 6.0000 | * | 762.0000 | 359.2798 | 402.7202 | 6570.2395 | 17634.2046 | 1.0000 |
| 9 | 8.0000 | * | 752.0000 | 325.3804 | 426.6196 | 8084.2395 | 28222.2046 | 1.0000 |
| 9 | 10.0000 | * | 300.0000 | 294.6795 | 5.3205 | 9136.2395 | 37238.2046 | 1.0000 |
| 9 | 12.0000 | * | 223.0000 | 266.8754 | -43.8754 | 9659.2395 | 42914.2046 | 1.0000 |
| 9 | 24.0000 | * | 59.2000 | 147.2516 | -88.0516 | 11352.4395 | 67495.0046 | 1.0000 |
| 9 | 36.0000 | * | 25.8000 | 81.2478 | -55.4478 | 11862.4395 | 81592.6046 | 1.0000 |
| 9 | 48.0000 | * | 20.8000 | 44.8294 | -24.0294 | 12142.0395 | 93155.8046 | 1.0000 |
| 9 | 72.0000 | * | 10.0000 | 13.6479 | -3.6479 | 12511.6395 | 113776.6046 | 1.0000 |
| 9 | 96.0000 | * | 13.2000 | 4.1550 | 9.0450 | 12790.0395 | 137623.0046 | 1.0000 |
| 10 | 0.0000 |  | 0.0000 |  |  | 0.0000 | 0.0000 | 0.0000 |
| 10 | 0.1667 |  | 215.0000 |  |  | 17.9203 | 2.9873 | 0.0000 |
| 10 | 0.5000 |  | 846.0000 |  |  | 194.7359 | 79.4531 | 0.0000 |
| 10 | 1.0000 |  | 1300.0000 |  |  | 731.2359 | 510.2031 | 0.0000 |
| 10 | 2.0000 |  | 2440.0000 |  |  | 2601.2359 | 3600.2031 | 0.0000 |
| 10 | 4.0000 |  | 980.0000 |  |  | 6021.2359 | 12400.2031 | 0.0000 |
| 10 | 6.0000 |  | 708.0000 |  |  | 7709.2359 | 20568.2031 | 0.0000 |
| 10 | 8.0000 |  | 468.0000 |  |  | 8885.2359 | 28560.2031 | 0.0000 |
| 10 | 10.0000 |  | 274.0000 |  |  | 9627.2359 | 35044.2031 | 0.0000 |
| 10 | 12.0000 |  | 280.0000 |  |  | 10181.2359 | 41144.2031 | 0.0000 |
| 10 | 24.0000 |  | 90.0000 |  |  | 12401.2359 | 74264.2031 | 0.0000 |
| 10 | 36.0000 |  | 34.4000 |  |  | 13147.6359 | 94654.6031 | 0.0000 |
| 10 | 48.0000 | * | 30.6000 | 31.0958 | -0.4958 | 13537.6359 | 110897.8031 | 1.0000 |
| 10 | 72.0000 | * | 19.6000 | 18.9799 | 0.6201 | 14140.0359 | 145457.8031 | 1.0000 |
| 10 | 96.0000 | * | 11.4000 | 11.5847 | -0.1847 | 14512.0359 | 175525.0031 | 1.0000 |
| 11 | 0.0000 |  | 0.0000 |  |  | 0.0000 | 0.0000 | 0.0000 |
| 11 | 0.1667 |  | 246.0000 |  |  | 20.5041 | 3.4180 | 0.0000 |
| 11 | 0.5000 |  | 942.0000 |  |  | 218.4843 | 88.7442 | 0.0000 |
| 11 | 1.0000 |  | 1030.0000 |  |  | 711.4843 | 463.9942 | 0.0000 |
| 11 | 2.0000 |  | 1950.0000 |  |  | 2201.4843 | 2928.9942 | 0.0000 |
| 11 | 4.0000 |  | 710.0000 |  |  | 4861.4843 | 9668.9942 | 0.0000 |
| 11 | 6.0000 |  | 396.0000 |  |  | 5967.4843 | 14884.9942 | 0.0000 |
| 11 | 8.0000 |  | 556.0000 |  |  | 6919.4843 | 21708.9942 | 0.0000 |
| 11 | 10.0000 |  | 396.0000 |  |  | 7871.4843 | 30116.9942 | 0.0000 |
| 11 | 12.0000 |  | 106.0000 |  |  | 8373.4843 | 35348.9942 | 0.0000 |
| 11 | 24.0000 |  | 57.2000 |  |  | 9352.6843 | 51217.7942 | 0.0000 |
| 11 | 36.0000 | * | 35.2000 | 35.9831 | -0.7831 | 9907.0843 | 67057.7942 | 1.0000 |
| 11 | 48.0000 | * | 28.6000 | 27.3976 | 1.2024 | 10289.8843 | 82897.7942 | 1.0000 |
| 11 | 72.0000 | * | 15.4000 | 15.8832 | -0.4832 | 10817.8843 | 112676.9942 | 1.0000 |
| 11 | 96.0000 | * | 9.3000 | 9.2080 | 0.0920 | 11114.2843 | 136696.1942 | 1.0000 |
| 12 | 0.0000 |  | 0.0000 |  |  | 0.0000 | 0.0000 | 0.0000 |
| 12 | 0.1667 |  | 171.0000 |  |  | 14.2529 | 2.3760 | 0.0000 |
| 12 | 0.5000 |  | 813.0000 |  |  | 178.2365 | 74.8697 | 0.0000 |
| 12 | 1.0000 |  | 1430.0000 |  |  | 738.9865 | 533.9947 | 0.0000 |
| 12 | 2.0000 |  | 2110.0000 |  |  | 2508.9865 | 3358.9947 | 0.0000 |
| 12 | 4.0000 |  | 810.0000 |  |  | 5428.9865 | 10818.9947 | 0.0000 |
| 12 | 6.0000 |  | 558.0000 |  |  | 6796.9865 | 17406.9947 | 0.0000 |
| 12 | 8.0000 |  | 380.0000 |  |  | 7734.9865 | 23794.9947 | 0.0000 |
| 12 | 10.0000 |  | 240.0000 |  |  | 8354.9865 | 29234.9947 | 0.0000 |
| 12 | 12.0000 |  | 153.0000 |  |  | 8747.9865 | 33470.9947 | 0.0000 |
| 12 | 24.0000 |  | 47.6000 |  |  | 9951.5865 | 51341.3947 | 0.0000 |
| 12 | 36.0000 | * | 25.2000 | 25.5645 | -0.3645 | 10388.3865 | 63638.9947 | 1.0000 |
| 12 | 48.0000 | * | 19.0000 | 19.5039 | -0.5039 | 10653.5865 | 74554.1947 | 1.0000 |
| 12 | 72.0000 | * | 12.4000 | 11.3525 | 1.0475 | 11030.3865 | 96211.7947 | 1.0000 |
| 12 | 96.0000 | * | 6.3000 | 6.6079 | -0.3079 | 11254.7865 | 114182.9947 | 1.0000 |
| 13 | 0.0000 |  | 0.0000 |  |  | 0.0000 | 0.0000 | 0.0000 |
| 13 | 0.1667 |  | 115.0000 |  |  | 9.5853 | 1.5979 | 0.0000 |
| 13 | 0.5000 |  | 981.0000 |  |  | 192.2337 | 86.5345 | 0.0000 |
| 13 | 1.0000 |  | 1120.0000 |  |  | 717.4837 | 489.1595 | 0.0000 |
| 13 | 2.0000 |  | 2070.0000 |  |  | 2312.4837 | 3119.1595 | 0.0000 |
| 13 | 4.0000 | * | 800.0000 | 405.4022 | 394.5978 | 5182.4837 | 10459.1595 | 1.0000 |
| 13 | 6.0000 | * | 672.0000 | 362.5513 | 309.4487 | 6654.4837 | 17691.1595 | 1.0000 |
| 13 | 8.0000 | * | 504.0000 | 324.2297 | 179.7703 | 7830.4837 | 25755.1595 | 1.0000 |
| 13 | 10.0000 | * | 226.0000 | 289.9588 | -63.9588 | 8560.4837 | 32047.1595 | 1.0000 |
| 13 | 12.0000 | * | 274.0000 | 259.3102 | 14.6898 | 9060.4837 | 37595.1595 | 1.0000 |
| 13 | 24.0000 | * | 88.0000 | 132.6539 | -44.6539 | 11232.4837 | 69995.1595 | 1.0000 |
| 13 | 36.0000 | * | 27.4000 | 67.8610 | -40.4610 | 11924.8837 | 88585.5595 | 1.0000 |
| 13 | 48.0000 | * | 10.6000 | 34.7153 | -24.1153 | 12152.8837 | 97556.7595 | 1.0000 |
| 13 | 72.0000 | * | 9.4000 | 9.0849 | 0.3151 | 12392.8837 | 111783.9595 | 1.0000 |
| 13 | 96.0000 | * | 6.0000 | 2.3775 | 3.6225 | 12577.6837 | 126817.5595 | 1.0000 |
| 14 | 0.0000 |  | 0.0000 |  |  | 0.0000 | 0.0000 | 0.0000 |
| 14 | 0.1667 |  | 218.0000 |  |  | 18.1703 | 3.0290 | 0.0000 |
| 14 | 0.5000 |  | 912.0000 |  |  | 206.4848 | 85.0776 | 0.0000 |
| 14 | 1.0000 |  | 1140.0000 |  |  | 719.4848 | 484.0776 | 0.0000 |
| 14 | 2.0000 |  | 1930.0000 |  |  | 2254.4848 | 2984.0776 | 0.0000 |
| 14 | 4.0000 |  | 1330.0000 |  |  | 5514.4848 | 12164.0776 | 0.0000 |
| 14 | 6.0000 |  | 852.0000 |  |  | 7696.4848 | 22596.0776 | 0.0000 |
| 14 | 8.0000 |  | 888.0000 |  |  | 9436.4848 | 34812.0776 | 0.0000 |
| 14 | 10.0000 |  | 242.0000 |  |  | 10566.4848 | 44336.0776 | 0.0000 |
| 14 | 12.0000 |  | 230.0000 |  |  | 11038.4848 | 49516.0776 | 0.0000 |
| 14 | 24.0000 |  | 73.6000 |  |  | 12860.0848 | 76674.4776 | 0.0000 |
| 14 | 36.0000 |  | 36.4000 |  |  | 13520.0848 | 95135.2776 | 0.0000 |
| 14 | 48.0000 | * | 25.4000 | 25.2910 | 0.1090 | 13890.8848 | 110312.8776 | 1.0000 |
| 14 | 72.0000 | * | 17.8000 | 17.9537 | -0.1537 | 14409.2848 | 140322.4776 | 1.0000 |
| 14 | 96.0000 | * | 12.8000 | 12.7451 | 0.0549 | 14776.4848 | 170447.2776 | 1.0000 |
| 15 | 0.0000 |  | 0.0000 |  |  | 0.0000 | 0.0000 | 0.0000 |
| 15 | 0.1667 |  | 220.0000 |  |  | 18.3370 | 3.0568 | 0.0000 |
| 15 | 0.5000 |  | 813.0000 |  |  | 190.4865 | 76.9117 | 0.0000 |
| 15 | 1.0000 |  | 1550.0000 |  |  | 781.2365 | 566.0367 | 0.0000 |
| 15 | 2.0000 |  | 1930.0000 |  |  | 2521.2365 | 3271.0367 | 0.0000 |
| 15 | 4.0000 |  | 1140.0000 |  |  | 5591.2365 | 11691.0367 | 0.0000 |
| 15 | 6.0000 | * | 630.0000 | 270.1663 | 359.8337 | 7361.2365 | 20031.0367 | 1.0000 |
| 15 | 8.0000 | * | 500.0000 | 244.5943 | 255.4057 | 8491.2365 | 27811.0367 | 1.0000 |
| 15 | 10.0000 | * | 248.0000 | 221.4427 | 26.5573 | 9239.2365 | 34291.0367 | 1.0000 |
| 15 | 12.0000 | * | 167.0000 | 200.4824 | -33.4824 | 9654.2365 | 38775.0367 | 1.0000 |
| 15 | 24.0000 | * | 54.8000 | 110.3993 | -55.5993 | 10985.0365 | 58690.2367 | 1.0000 |
| 15 | 36.0000 | * | 39.2000 | 60.7934 | -21.5934 | 11549.0365 | 75048.6367 | 1.0000 |
| 15 | 48.0000 | * | 9.6000 | 33.4770 | -23.8770 | 11841.8365 | 86280.6367 | 1.0000 |
| 15 | 72.0000 | * | 11.6000 | 10.1514 | 1.4486 | 12096.2365 | 101832.6367 | 1.0000 |
| 15 | 96.0000 | * | 6.6000 | 3.0783 | 3.5217 | 12314.6365 | 119458.2367 | 1.0000 |
| 16 | 0.0000 |  | 0.0000 |  |  | 0.0000 | 0.0000 | 0.0000 |
| 16 | 0.1667 |  | 174.0000 |  |  | 14.5029 | 2.4176 | 0.0000 |
| 16 | 0.5000 |  | 606.0000 |  |  | 144.4899 | 57.7464 | 0.0000 |
| 16 | 1.0000 |  | 1380.0000 |  |  | 640.9899 | 478.4964 | 0.0000 |
| 16 | 2.0000 |  | 2470.0000 |  |  | 2565.9899 | 3638.4964 | 0.0000 |
| 16 | 4.0000 |  | 1140.0000 |  |  | 6175.9899 | 13138.4964 | 0.0000 |
| 16 | 6.0000 |  | 684.0000 |  |  | 7999.9899 | 21802.4964 | 0.0000 |
| 16 | 8.0000 |  | 476.0000 |  |  | 9159.9899 | 29714.4964 | 0.0000 |
| 16 | 10.0000 |  | 382.0000 |  |  | 10017.9899 | 37342.4964 | 0.0000 |
| 16 | 12.0000 |  | 78.0000 |  |  | 10477.9899 | 42098.4964 | 0.0000 |
| 16 | 24.0000 |  | 53.2000 |  |  | 11265.1899 | 55375.2964 | 0.0000 |
| 16 | 36.0000 |  | 21.8000 |  |  | 11715.1899 | 67744.8964 | 0.0000 |
| 16 | 48.0000 | * | 22.6000 | 23.8369 | -1.2369 | 11981.5899 | 78962.4964 | 1.0000 |
| 16 | 72.0000 | * | 19.8000 | 17.7984 | 2.0016 | 12490.3899 | 109087.2964 | 1.0000 |
| 16 | 96.0000 | * | 12.6000 | 13.2896 | -0.6896 | 12879.1899 | 140709.6964 | 1.0000 |
| 17 | 0.0000 |  | 0.0000 |  |  | 0.0000 | 0.0000 | 0.0000 |
| 17 | 0.1667 |  | 191.0000 |  |  | 15.9199 | 2.6538 | 0.0000 |
| 17 | 0.5000 |  | 795.0000 |  |  | 180.2368 | 74.2033 | 0.0000 |
| 17 | 1.0000 |  | 1360.0000 |  |  | 718.9868 | 513.5783 | 0.0000 |
| 17 | 2.0000 |  | 2870.0000 |  |  | 2833.9868 | 4063.5783 | 0.0000 |
| 17 | 4.0000 |  | 1160.0000 |  |  | 6863.9868 | 14443.5783 | 0.0000 |
| 17 | 6.0000 | * | 510.0000 | 274.0805 | 235.9195 | 8533.9868 | 22143.5783 | 1.0000 |
| 17 | 8.0000 | * | 552.0000 | 251.3459 | 300.6541 | 9595.9868 | 29619.5783 | 1.0000 |
| 17 | 10.0000 | * | 260.0000 | 230.4971 | 29.5029 | 10407.9868 | 36635.5783 | 1.0000 |
| 17 | 12.0000 | * | 238.0000 | 211.3777 | 26.6223 | 10905.9868 | 42091.5783 | 1.0000 |
| 17 | 24.0000 | * | 70.0000 | 125.7249 | -55.7249 | 12753.9868 | 69307.5783 | 1.0000 |
| 17 | 36.0000 | * | 29.4000 | 74.7796 | -45.3796 | 13350.3868 | 85737.9783 | 1.0000 |
| 17 | 48.0000 | * | 15.2000 | 44.4780 | -29.2780 | 13617.9868 | 96465.9783 | 1.0000 |
| 17 | 72.0000 | * | 18.8000 | 15.7351 | 3.0649 | 14025.9868 | 121464.3783 | 1.0000 |
| 17 | 96.0000 | * | 12.0000 | 5.5666 | 6.4334 | 14395.5868 | 151531.5783 | 1.0000 |
| 18 | 0.0000 |  | 0.0000 |  |  | 0.0000 | 0.0000 | 0.0000 |
| 18 | 0.1667 |  | 212.0000 |  |  | 17.6702 | 2.9456 | 0.0000 |
| 18 | 0.5000 |  | 762.0000 |  |  | 179.9873 | 72.3288 | 0.0000 |
| 18 | 1.0000 |  | 1580.0000 |  |  | 765.4873 | 562.5788 | 0.0000 |
| 18 | 2.0000 |  | 2680.0000 |  |  | 2895.4873 | 4032.5788 | 0.0000 |
| 18 | 4.0000 |  | 2080.0000 |  |  | 7655.4873 | 17712.5788 | 0.0000 |
| 18 | 6.0000 | * | 852.0000 | 373.0594 | 478.9406 | 10587.4873 | 31144.5788 | 1.0000 |
| 18 | 8.0000 | * | 472.0000 | 336.5227 | 135.4773 | 11911.4873 | 40032.5788 | 1.0000 |
| 18 | 10.0000 | * | 420.0000 | 303.5643 | 116.4357 | 12803.4873 | 48008.5788 | 1.0000 |
| 18 | 12.0000 | * | 339.0000 | 273.8337 | 65.1663 | 13562.4873 | 56276.5788 | 1.0000 |
| 18 | 24.0000 | * | 82.0000 | 147.5383 | -65.5383 | 16088.4873 | 92492.5788 | 1.0000 |
| 18 | 36.0000 | * | 29.2000 | 79.4919 | -50.2919 | 16755.6873 | 110607.7788 | 1.0000 |
| 18 | 48.0000 | * | 15.2000 | 42.8293 | -27.6293 | 17022.0873 | 121292.5788 | 1.0000 |
| 18 | 72.0000 | * | 13.6000 | 12.4330 | 1.1670 | 17367.6873 | 141798.1788 | 1.0000 |
| 18 | 96.0000 | * | 8.3000 | 3.6092 | 4.6908 | 17630.4873 | 163110.1788 | 1.0000 |
| 19 | 0.0000 |  | 0.0000 |  |  | 0.0000 | 0.0000 | 0.0000 |
| 19 | 0.1667 |  | 128.0000 |  |  | 10.6688 | 1.7785 | 0.0000 |
| 19 | 0.5000 |  | 861.0000 |  |  | 175.4857 | 77.0772 | 0.0000 |
| 19 | 1.0000 |  | 1440.0000 |  |  | 750.7357 | 544.7022 | 0.0000 |
| 19 | 2.0000 |  | 2870.0000 |  |  | 2905.7357 | 4134.7022 | 0.0000 |
| 19 | 4.0000 |  | 1090.0000 |  |  | 6865.7357 | 14234.7022 | 0.0000 |
| 19 | 6.0000 |  | 978.0000 |  |  | 8933.7357 | 24462.7022 | 0.0000 |
| 19 | 8.0000 |  | 652.0000 |  |  | 10563.7357 | 35546.7022 | 0.0000 |
| 19 | 10.0000 |  | 372.0000 |  |  | 11587.7357 | 44482.7022 | 0.0000 |
| 19 | 12.0000 |  | 299.0000 |  |  | 12258.7357 | 51790.7022 | 0.0000 |
| 19 | 24.0000 |  | 87.6000 |  |  | 14578.3357 | 85933.1022 | 0.0000 |
| 19 | 36.0000 | * | 41.6000 | 36.6240 | 4.9760 | 15353.5357 | 107533.1022 | 1.0000 |
| 19 | 48.0000 | * | 25.4000 | 30.5052 | -5.1052 | 15755.5357 | 123833.9022 | 1.0000 |
| 19 | 72.0000 | * | 22.2000 | 21.1637 | 1.0363 | 16326.7357 | 157645.1022 | 1.0000 |
| 19 | 96.0000 | * | 14.8000 | 14.6828 | 0.1172 | 16770.7357 | 193875.5022 | 1.0000 |
| 20 | 0.0000 |  | 0.0000 |  |  | 0.0000 | 0.0000 | 0.0000 |
| 20 | 0.1667 |  | 210.0000 |  |  | 17.5035 | 2.9178 | 0.0000 |
| 20 | 0.5000 |  | 780.0000 |  |  | 182.4870 | 73.7453 | 0.0000 |
| 20 | 1.0000 |  | 1910.0000 |  |  | 854.9870 | 648.7453 | 0.0000 |
| 20 | 2.0000 | * | 2210.0000 | 776.9532 | 1433.0468 | 2914.9870 | 3813.7453 | 1.0000 |
| 20 | 4.0000 | * | 1650.0000 | 699.0294 | 950.9706 | 6774.9870 | 14833.7453 | 1.0000 |
| 20 | 6.0000 | * | 1014.0000 | 628.9210 | 385.0790 | 9438.9870 | 27517.7453 | 1.0000 |
| 20 | 8.0000 | * | 540.0000 | 565.8439 | -25.8439 | 10992.9870 | 37921.7453 | 1.0000 |
| 20 | 10.0000 | * | 502.0000 | 509.0932 | -7.0932 | 12034.9870 | 47261.7453 | 1.0000 |
| 20 | 12.0000 | * | 386.0000 | 458.0342 | -72.0342 | 12922.9870 | 56913.7453 | 1.0000 |
| 20 | 24.0000 | * | 94.8000 | 242.9414 | -148.1414 | 15807.7870 | 98356.9453 | 1.0000 |
| 20 | 36.0000 | * | 24.4000 | 128.8561 | -104.4561 | 16522.9870 | 117278.5453 | 1.0000 |
| 20 | 48.0000 | * | 28.0000 | 68.3453 | -40.3453 | 16837.3870 | 130612.9453 | 1.0000 |
| 20 | 72.0000 | * | 29.0000 | 19.2272 | 9.7728 | 17521.3870 | 171796.9453 | 1.0000 |
| 20 | 96.0000 | * | 13.8000 | 5.4091 | 8.3909 | 18034.9870 | 212750.5453 | 1.0000 |
| 21 | 0.0000 |  | 0.0000 |  |  | 0.0000 | 0.0000 | 0.0000 |
| 21 | 0.1667 |  | 160.0000 |  |  | 13.3360 | 2.2231 | 0.0000 |
| 21 | 0.5000 |  | 816.0000 |  |  | 175.9864 | 74.6612 | 0.0000 |
| 21 | 1.0000 |  | 1700.0000 |  |  | 804.9864 | 601.6612 | 0.0000 |
| 21 | 2.0000 | * | 2070.0000 | 714.7818 | 1355.2182 | 2689.9864 | 3521.6612 | 1.0000 |
| 21 | 4.0000 | * | 1770.0000 | 638.8180 | 1131.1820 | 6529.9864 | 14741.6612 | 1.0000 |
| 21 | 6.0000 | * | 900.0000 | 570.9272 | 329.0728 | 9199.9864 | 27221.6612 | 1.0000 |
| 21 | 8.0000 | * | 720.0000 | 510.2516 | 209.7484 | 10819.9864 | 38381.6612 | 1.0000 |
| 21 | 10.0000 | * | 324.0000 | 456.0244 | -132.0244 | 11863.9864 | 47381.6612 | 1.0000 |
| 21 | 12.0000 | * | 370.0000 | 407.5602 | -37.5602 | 12557.9864 | 55061.6612 | 1.0000 |
| 21 | 24.0000 | * | 71.2000 | 207.6891 | -136.4891 | 15205.1864 | 91954.4612 | 1.0000 |
| 21 | 36.0000 | * | 24.0000 | 105.8365 | -81.8365 | 15776.3864 | 107391.2612 | 1.0000 |
| 21 | 48.0000 | * | 13.8000 | 53.9334 | -40.1334 | 16003.1864 | 116549.6612 | 1.0000 |
| 21 | 72.0000 | * | 16.6000 | 14.0056 | 2.5944 | 16367.9864 | 138840.8612 | 1.0000 |
| 21 | 96.0000 | * | 13.4000 | 3.6370 | 9.7630 | 16727.9864 | 168620.0612 | 1.0000 |
| 22 | 0.0000 |  | 0.0000 |  |  | 0.0000 | 0.0000 | 0.0000 |
| 22 | 0.1667 |  | 186.0000 |  |  | 15.5031 | 2.5844 | 0.0000 |
| 22 | 0.5000 |  | 723.0000 |  |  | 166.9880 | 67.9955 | 0.0000 |
| 22 | 1.0000 |  | 1930.0000 |  |  | 830.2380 | 640.8705 | 0.0000 |
| 22 | 2.0000 |  | 1830.0000 |  |  | 2710.2380 | 3435.8705 | 0.0000 |
| 22 | 4.0000 |  | 1150.0000 |  |  | 5690.2380 | 11695.8705 | 0.0000 |
| 22 | 6.0000 |  | 732.0000 |  |  | 7572.2380 | 20687.8705 | 0.0000 |
| 22 | 8.0000 |  | 548.0000 |  |  | 8852.2380 | 29463.8705 | 0.0000 |
| 22 | 10.0000 |  | 282.0000 |  |  | 9682.2380 | 36667.8705 | 0.0000 |
| 22 | 12.0000 |  | 152.0000 |  |  | 10116.2380 | 41311.8705 | 0.0000 |
| 22 | 24.0000 |  | 39.6000 |  |  | 11265.8380 | 57958.2705 | 0.0000 |
| 22 | 36.0000 |  | 22.2000 |  |  | 11636.6380 | 68455.8705 | 0.0000 |
| 22 | 48.0000 | * | 14.4000 | 14.2859 | 0.1141 | 11856.2380 | 77398.2705 | 1.0000 |
| 22 | 72.0000 | * | 12.4000 | 12.5989 | -0.1989 | 12177.8380 | 96406.2705 | 1.0000 |
| 22 | 96.0000 | * | 11.2000 | 11.1112 | 0.0888 | 12461.0380 | 120022.2705 | 1.0000 |
| 23 | 0.0000 |  | 0.0000 |  |  | 0.0000 | 0.0000 | 0.0000 |
| 23 | 0.1667 |  | 214.0000 |  |  | 17.8369 | 2.9734 | 0.0000 |
| 23 | 0.5000 |  | 900.0000 |  |  | 203.4850 | 83.9110 | 0.0000 |
| 23 | 1.0000 |  | 1500.0000 |  |  | 803.4850 | 571.4110 | 0.0000 |
| 23 | 2.0000 |  | 2140.0000 |  |  | 2623.4850 | 3461.4110 | 0.0000 |
| 23 | 4.0000 |  | 1180.0000 |  |  | 5943.4850 | 12461.4110 | 0.0000 |
| 23 | 6.0000 | * | 432.0000 | 267.5002 | 164.4998 | 7555.4850 | 19773.4110 | 1.0000 |
| 23 | 8.0000 | * | 600.0000 | 245.4291 | 354.5709 | 8587.4850 | 27165.4110 | 1.0000 |
| 23 | 10.0000 | * | 280.0000 | 225.1790 | 54.8210 | 9467.4850 | 34765.4110 | 1.0000 |
| 23 | 12.0000 | * | 193.0000 | 206.5997 | -13.5997 | 9940.4850 | 39881.4110 | 1.0000 |
| 23 | 24.0000 | * | 62.8000 | 123.2370 | -60.4370 | 11475.2850 | 62820.6110 | 1.0000 |
| 23 | 36.0000 | * | 34.4000 | 73.5110 | -39.1110 | 12058.4850 | 79294.2110 | 1.0000 |
| 23 | 48.0000 | * | 15.2000 | 43.8494 | -28.6494 | 12356.0850 | 91102.2110 | 1.0000 |
| 23 | 72.0000 | * | 23.8000 | 15.6022 | 8.1978 | 12824.0850 | 120420.6110 | 1.0000 |
| 23 | 96.0000 | * | 9.6000 | 5.5515 | 4.0485 | 13224.8850 | 152043.0110 | 1.0000 |
| 24 | 0.0000 |  | 0.0000 |  |  | 0.0000 | 0.0000 | 0.0000 |
| 24 | 0.1667 |  | 172.0000 |  |  | 14.3362 | 2.3898 | 0.0000 |
| 24 | 0.5000 |  | 786.0000 |  |  | 173.9869 | 72.6616 | 0.0000 |
| 24 | 1.0000 |  | 1790.0000 |  |  | 817.9869 | 618.4116 | 0.0000 |
| 24 | 2.0000 |  | 2250.0000 |  |  | 2837.9869 | 3763.4116 | 0.0000 |
| 24 | 4.0000 |  | 1560.0000 |  |  | 6647.9869 | 14503.4116 | 0.0000 |
| 24 | 6.0000 |  | 786.0000 |  |  | 8993.9869 | 25459.4116 | 0.0000 |
| 24 | 8.0000 |  | 388.0000 |  |  | 10167.9869 | 33279.4116 | 0.0000 |
| 24 | 10.0000 |  | 290.0000 |  |  | 10845.9869 | 39283.4116 | 0.0000 |
| 24 | 12.0000 |  | 316.0000 |  |  | 11451.9869 | 45975.4116 | 0.0000 |
| 24 | 24.0000 |  | 90.4000 |  |  | 13890.3869 | 81745.0116 | 0.0000 |
| 24 | 36.0000 | * | 35.2000 | 36.1389 | -0.9389 | 14643.9869 | 102365.8116 | 1.0000 |
| 24 | 48.0000 | * | 26.4000 | 26.0959 | 0.3041 | 15013.5869 | 117572.2116 | 1.0000 |
| 24 | 72.0000 | * | 14.2000 | 13.6072 | 0.5928 | 15500.7869 | 145047.4116 | 1.0000 |
| 24 | 96.0000 | * | 6.9000 | 7.0952 | -0.1952 | 15753.9869 | 165265.0116 | 1.0000 |

Worksheet: Summary Table

| **User_Defined_Settings** |
| --- |
| NCA Model 200 - [??????-??????????] |
| Linear trapezoidal (Linear Interpolation) method used |

Worksheet: User Settings

Worksheet: History

| **Variable** | **Parameter** | **N** | **Nmiss** | **Nobs** | **Mean** | **SD** | **SE** | **Variance** | **Min** | **Median** | **Max** | **Range** | **CV%** | **Geometric_Mean** | **Harmonic_Mean** | **Pseudo_SD** | **Mean_Log** | **SD_Log** | **CV%_Geometric_Mean** | **Skewness** | **Kurtosis** | **KS_pvalue** |
| --- | --- | --- | --- | --- | --- | --- | --- | --- | --- | --- | --- | --- | --- | --- | --- | --- | --- | --- | --- | --- | --- | --- |
| Estimate | AUC_%Extrap_obs | 24 | 0 | 24 | 3.4691 | 3.1400 | 0.6410 | 9.8599 | 0.8468 | 2.2313 | 14.6515 | 13.8047 | 90.5154 | 2.5915 | 2.0553 | 1.3322 | 0.9522 | 0.7480 | 86.5959 | 2.1188 | 4.7735 | 0.2384 |
| Estimate | AUC_%Extrap_pred | 24 | 0 | 24 | 3.1130 | 3.3150 | 0.6767 | 10.9889 | 0.3373 | 1.5879 | 14.5523 | 14.2150 | 106.4864 | 1.8562 | 1.1189 | 1.1556 | 0.6185 | 1.0797 | 148.6000 | 1.8982 | 3.9141 | 0.2214 |
| Estimate | AUCall | 24 | 0 | 24 | 14550.8347 | 1992.0914 | 406.6340 | 3968428.0758 | 11114.2843 | 14453.8113 | 18034.9870 | 6920.7027 | 13.6906 | 14418.6515 | 14285.5498 | 2008.6610 | 9.5763 | 0.1387 | 13.9333 | 0.0238 | -1.0308 | 0.9333 |
| Estimate | AUCINF_D_obs | 24 | 0 | 24 | 7094.0053 | 938.7769 | 191.6270 | 881302.1451 | 5422.9086 | 6997.4060 | 8609.9458 | 3187.0372 | 13.2334 | 7032.8324 | 6970.2115 | 971.4358 | 8.8583 | 0.1356 | 13.6243 | -0.1589 | -0.9628 | 0.9980 |
| Estimate | AUCINF_D_pred | 24 | 0 | 24 | 7068.5038 | 936.7371 | 191.2107 | 877476.4644 | 5421.0020 | 6978.4567 | 8535.2221 | 3114.2202 | 13.2523 | 7007.3745 | 6944.8299 | 968.4438 | 8.8547 | 0.1358 | 13.6432 | -0.1643 | -1.0132 | 0.9880 |
| Estimate | AUCINF_obs | 24 | 0 | 24 | 15074.7612 | 1994.9010 | 407.2075 | 3979629.9989 | 11523.6808 | 14869.4878 | 18296.1348 | 6772.4540 | 13.2334 | 14944.7689 | 14811.6994 | 2064.3011 | 9.6121 | 0.1356 | 13.6243 | -0.1589 | -0.9628 | 0.9980 |
| Estimate | AUCINF_pred | 24 | 0 | 24 | 15020.5707 | 1990.5664 | 406.3227 | 3962354.6596 | 11519.6292 | 14829.2205 | 18137.3470 | 6617.7178 | 13.2523 | 14890.6707 | 14757.7636 | 2057.9431 | 9.6085 | 0.1358 | 13.6432 | -0.1643 | -1.0132 | 0.9880 |
| Estimate | AUClast | 24 | 0 | 24 | 14550.8347 | 1992.0914 | 406.6340 | 3968428.0758 | 11114.2843 | 14453.8113 | 18034.9870 | 6920.7027 | 13.6906 | 14418.6515 | 14285.5498 | 2008.6610 | 9.5763 | 0.1387 | 13.9333 | 0.0238 | -1.0308 | 0.9333 |
| Estimate | AUMC_%Extrap_obs | 24 | 0 | 24 | 27.8105 | 18.8757 | 3.8530 | 356.2915 | 8.7989 | 19.6892 | 83.6471 | 74.8481 | 67.8725 | 22.9975 | 19.5252 | 10.7294 | 3.1354 | 0.6153 | 67.8368 | 1.3763 | 1.3851 | 0.3409 |
| Estimate | AUMC_%Extrap_pred | 24 | 0 | 24 | 24.8603 | 21.0644 | 4.2997 | 443.7080 | 3.6822 | 16.6903 | 83.5379 | 79.8557 | 84.7310 | 16.9851 | 11.3902 | 10.5691 | 2.8323 | 0.9381 | 118.7781 | 1.0988 | 0.6248 | 0.4142 |
| Estimate | AUMCINF_obs | 24 | 0 | 24 | 252570.0008 | 124373.6854 | 25387.6722 | 15468813621.9795 | 134871.9461 | 211799.1868 | 733950.1796 | 599078.2335 | 49.2433 | 233049.0345 | 219126.2124 | 71836.9800 | 12.3590 | 0.3818 | 39.6167 | 2.5419 | 7.4361 | 0.4767 |
| Estimate | AUMCINF_pred | 24 | 0 | 24 | 245872.7307 | 125649.5814 | 25648.1134 | 15787817298.2602 | 126647.2666 | 208733.5961 | 729083.8243 | 602436.5576 | 51.1035 | 225190.5929 | 210329.2553 | 72933.9512 | 12.3247 | 0.4009 | 41.7589 | 2.4687 | 7.1519 | 0.4816 |
| Estimate | AUMClast | 24 | 0 | 24 | 161973.5287 | 27785.6931 | 5671.7309 | 772044742.4983 | 114182.9947 | 162633.8417 | 212750.5453 | 98567.5506 | 17.1545 | 159625.5861 | 157227.1102 | 28601.3106 | 11.9806 | 0.1764 | 17.7731 | -0.0404 | -0.9125 | 0.9674 |
| Estimate | Cl_F_obs | 24 | 0 | 24 | 0.1435 | 0.0199 | 0.0041 | 0.0004 | 0.1161 | 0.1429 | 0.1844 | 0.0683 | 13.8881 | 0.1422 | 0.1410 | 0.0186 | -1.9506 | 0.1356 | 13.6243 | 0.5779 | -0.5691 | 0.9090 |
| Estimate | Cl_F_pred | 24 | 0 | 24 | 0.1440 | 0.0200 | 0.0041 | 0.0004 | 0.1172 | 0.1433 | 0.1845 | 0.0673 | 13.8975 | 0.1427 | 0.1415 | 0.0187 | -1.9470 | 0.1358 | 13.6432 | 0.5588 | -0.6641 | 0.8762 |
| Estimate | Clast | 24 | 0 | 24 | 10.8625 | 3.1082 | 0.6345 | 9.6607 | 5.8000 | 11.3000 | 15.4000 | 9.6000 | 28.6137 | 10.3906 | 9.8862 | 3.3926 | 2.3409 | 0.3147 | 32.2642 | -0.2339 | -1.1912 | 0.8367 |
| Estimate | Cmax | 24 | 0 | 24 | 2257.5000 | 279.5066 | 57.0540 | 78123.9130 | 1930.0000 | 2190.0000 | 2870.0000 | 940.0000 | 12.3812 | 2241.8258 | 2227.0468 | 254.4814 | 7.7150 | 0.1191 | 11.9501 | 0.8607 | -0.1725 | 0.3363 |
| Estimate | Cmax_D | 24 | 0 | 24 | 1062.3529 | 131.5325 | 26.8490 | 17300.7974 | 908.2353 | 1030.5882 | 1350.5882 | 442.3529 | 12.3812 | 1054.9768 | 1048.0220 | 119.7560 | 6.9613 | 0.1191 | 11.9501 | 0.8607 | -0.1725 | 0.3363 |
| Estimate | Corr_XY | 24 | 0 | 24 | -0.9497 | 0.0463 | 0.0095 | 0.0021 | -1.0000 | -0.9523 | -0.8735 | 0.1265 | -4.8775 | Missing | Missing | Missing | Missing | Missing | Missing | 0.2363 | -1.5510 | 0.1944 |
| Estimate | HL_Lambda_z | 24 | 0 | 24 | 31.7741 | 25.7539 | 5.2570 | 663.2652 | 12.3382 | 24.5763 | 132.3882 | 120.0500 | 81.0534 | 25.8362 | 22.1173 | 11.3403 | 3.2518 | 0.6143 | 67.7094 | 2.6283 | 7.7850 | 0.1751 |
| Estimate | Lambda_z | 24 | 0 | 24 | 0.0313 | 0.0160 | 0.0033 | 0.0003 | 0.0052 | 0.0282 | 0.0562 | 0.0509 | 51.0343 | 0.0268 | 0.0218 | 0.0196 | -3.6183 | 0.6143 | 67.7094 | 0.1726 | -1.3413 | 0.5430 |
| Estimate | Lambda_z_lower | 24 | 0 | 24 | 25.7500 | 18.5595 | 3.7885 | 344.4565 | 2.0000 | 36.0000 | 48.0000 | 46.0000 | 72.0759 | 16.3711 | 8.6400 | 11.6758 | 2.7955 | 1.1302 | 160.8567 | -0.1033 | -1.6892 | 0.0965 |
| Estimate | Lambda_z_upper | 24 | 0 | 24 | 96.0000 | 0.0000 | 0.0000 | 0.0000 | 96.0000 | 96.0000 | 96.0000 | 0.0000 | 0.0000 | 96.0000 | 96.0000 | 0.0000 | 4.5643 | 0.0000 | 0.0000 | 0.0000 | 0.0000 | Missing |
| Estimate | MRTINF_obs | 24 | 0 | 24 | 16.7453 | 8.1729 | 1.6683 | 66.7966 | 10.2125 | 13.8189 | 50.2699 | 40.0574 | 48.8073 | 15.5940 | 14.8450 | 4.1262 | 2.7469 | 0.3492 | 36.0124 | 3.0352 | 9.9801 | 0.1287 |
| Estimate | MRTINF_pred | 24 | 0 | 24 | 16.3485 | 8.2793 | 1.6900 | 68.5474 | 9.6716 | 13.2713 | 49.9947 | 40.3231 | 50.6428 | 15.1229 | 14.3135 | 4.2542 | 2.7162 | 0.3674 | 38.0182 | 2.9383 | 9.5159 | 0.2108 |
| Estimate | MRTlast | 24 | 0 | 24 | 11.1193 | 1.0684 | 0.2181 | 1.1415 | 9.2516 | 11.0981 | 13.8092 | 4.5576 | 9.6085 | 11.0708 | 11.0229 | 1.0461 | 2.4043 | 0.0953 | 9.5564 | 0.3765 | -0.0292 | 0.9962 |
| Estimate | No_points_lambda_z | 24 | 0 | 24 | 6.0000 | 3.0217 | 0.6168 | 9.1304 | 3.0000 | 4.0000 | 11.0000 | 8.0000 | 50.3610 | 5.3131 | 4.7487 | 2.1357 | 1.6702 | 0.5018 | 53.5066 | 0.4733 | -1.4947 | 0.0377 |
| Estimate | Rsq | 24 | 0 | 24 | 0.9040 | 0.0875 | 0.0179 | 0.0077 | 0.7630 | 0.9068 | 1.0000 | 0.2370 | 9.6791 | 0.8999 | 0.8957 | 0.0891 | -0.1055 | 0.0982 | 9.8460 | -0.2082 | -1.5755 | 0.1953 |
| Estimate | Rsq_adjusted | 24 | 0 | 24 | 0.8815 | 0.1015 | 0.0207 | 0.0103 | 0.7366 | 0.8685 | 1.0000 | 0.2633 | 11.5187 | 0.8759 | 0.8702 | 0.1017 | -0.1325 | 0.1163 | 11.6727 | -0.0617 | -1.6561 | 0.1973 |
| Estimate | Tlag | 24 | 0 | 24 | 0.0000 | 0.0000 | 0.0000 | 0.0000 | 0.0000 | 0.0000 | 0.0000 | 0.0000 | Missing | Missing | Missing | Missing | Missing | Missing | Missing | 0.0000 | 0.0000 | Missing |
| Estimate | Tlast | 24 | 0 | 24 | 96.0000 | 0.0000 | 0.0000 | 0.0000 | 96.0000 | 96.0000 | 96.0000 | 0.0000 | 0.0000 | 96.0000 | 96.0000 | 0.0000 | 4.5643 | 0.0000 | 0.0000 | 0.0000 | 0.0000 | Missing |
| Estimate | Tmax | 24 | 0 | 24 | 1.9583 | 0.2041 | 0.0417 | 0.0417 | 1.0000 | 2.0000 | 2.0000 | 1.0000 | 10.4234 | 1.9431 | 1.9200 | 0.3912 | 0.6643 | 0.1415 | 14.2199 | -4.5873 | 19.0435 | 0.0000 |
| Estimate | Vz_F_obs | 24 | 0 | 24 | 6.5401 | 5.3685 | 1.0958 | 28.8207 | 2.1979 | 4.9333 | 27.7987 | 25.6008 | 82.0857 | 5.3000 | 4.5066 | 2.4565 | 1.6677 | 0.6218 | 68.7027 | 2.7229 | 8.3552 | 0.2216 |
| Estimate | Vz_F_pred | 24 | 0 | 24 | 6.5538 | 5.3686 | 1.0959 | 28.8221 | 2.2171 | 4.9275 | 27.8310 | 25.6139 | 81.9160 | 5.3192 | 4.5305 | 2.4548 | 1.6713 | 0.6189 | 68.3188 | 2.7303 | 8.3912 | 0.2142 |

Worksheet: Sheet1
