# Supplementary material for: Pharmacokinetics and bioequivalence of two imidocarb formulations in cattle after subcutaneous injection
Source: PLoS One. 2022 Jun 24;17(6):e0270130. doi: 10.1371/journal.pone.0270130 (PMC9231748; doi:10.1371/journal.pone.0270130)
Supplement: S2 File — (DOC) [file pone.0270130.s003.doc]

| **Animal** | **Time (hr)** | **Concentration (ug/L)** |
| --- | --- | --- |
| 25 | 0.1667 | 210 |
| 25 | 0.5 | 465 |
| 25 | 1 | 1550 |
| 25 | 2 | 2220 |
| 25 | 4 | 1390 |
| 25 | 6 | 1020 |
| 25 | 8 | 468 |
| 25 | 10 | 398 |
| 25 | 12 | 199 |
| 25 | 24 | 75.2 |
| 25 | 36 | 32.2 |
| 25 | 48 | 26.6 |
| 25 | 72 | 16.8 |
| 25 | 96 | 14.2 |
| 26 | 0.1667 | 237 |
| 26 | 0.5 | 726 |
| 26 | 1 | 1500 |
| 26 | 2 | 2490 |
| 26 | 4 | 1240 |
| 26 | 6 | 654 |
| 26 | 8 | 704 |
| 26 | 10 | 296 |
| 26 | 12 | 288 |
| 26 | 24 | 73.6 |
| 26 | 36 | 30.2 |
| 26 | 48 | 28.4 |
| 26 | 72 | 16.6 |
| 26 | 96 | 12 |
| 27 | 0.1667 | 126 |
| 27 | 0.5 | 699 |
| 27 | 1 | 1350 |
| 27 | 2 | 2750 |
| 27 | 4 | 1370 |
| 27 | 6 | 696 |
| 27 | 8 | 452 |
| 27 | 10 | 330 |
| 27 | 12 | 232 |
| 27 | 24 | 106.4 |
| 27 | 36 | 34.8 |
| 27 | 48 | 26.4 |
| 27 | 72 | 14.8 |
| 27 | 96 | 12.6 |
| 28 | 0.1667 | 160 |
| 28 | 0.5 | 819 |
| 28 | 1 | 1650 |
| 28 | 2 | 2570 |
| 28 | 4 | 1430 |
| 28 | 6 | 1128 |
| 28 | 8 | 700 |
| 28 | 10 | 426 |
| 28 | 12 | 263 |
| 28 | 24 | 157.6 |
| 28 | 36 | 47.6 |
| 28 | 48 | 25.8 |
| 28 | 72 | 18.2 |
| 28 | 96 | 12.6 |
| 29 | 0.1667 | 222 |
| 29 | 0.5 | 837 |
| 29 | 1 | 1430 |
| 29 | 2 | 2210 |
| 29 | 4 | 1830 |
| 29 | 6 | 708 |
| 29 | 8 | 828 |
| 29 | 10 | 438 |
| 29 | 12 | 312 |
| 29 | 24 | 125.6 |
| 29 | 36 | 43.8 |
| 29 | 48 | 32 |
| 29 | 72 | 21.6 |
| 29 | 96 | 11.4 |
| 30 | 0.1667 | 139 |
| 30 | 0.5 | 645 |
| 30 | 1 | 1850 |
| 30 | 2 | 2100 |
| 30 | 4 | 1760 |
| 30 | 6 | 1038 |
| 30 | 8 | 780 |
| 30 | 10 | 366 |
| 30 | 12 | 236 |
| 30 | 24 | 94 |
| 30 | 36 | 31.6 |
| 30 | 48 | 34.2 |
| 30 | 72 | 14.4 |
| 30 | 96 | 14.5 |
| 31 | 0.1667 | 199 |
| 31 | 0.5 | 804 |
| 31 | 1 | 1540 |
| 31 | 2 | 2330 |
| 31 | 4 | 1080 |
| 31 | 6 | 666 |
| 31 | 8 | 772 |
| 31 | 10 | 384 |
| 31 | 12 | 210 |
| 31 | 24 | 98.4 |
| 31 | 36 | 39.4 |
| 31 | 48 | 29.4 |
| 31 | 72 | 13.4 |
| 31 | 96 | 20 |
| 32 | 0.1667 | 208 |
| 32 | 0.5 | 927 |
| 32 | 1 | 1760 |
| 32 | 2 | 1610 |
| 32 | 4 | 980 |
| 32 | 6 | 528 |
| 32 | 8 | 596 |
| 32 | 10 | 334 |
| 32 | 12 | 224 |
| 32 | 24 | 76.8 |
| 32 | 36 | 35.4 |
| 32 | 48 | 27.4 |
| 32 | 72 | 15.8 |
| 32 | 96 | 15.6 |
| 33 | 0.1667 | 212 |
| 33 | 0.5 | 717 |
| 33 | 1 | 1220 |
| 33 | 2 | 2380 |
| 33 | 4 | 1340 |
| 33 | 6 | 558 |
| 33 | 8 | 536 |
| 33 | 10 | 374 |
| 33 | 12 | 203 |
| 33 | 24 | 86 |
| 33 | 36 | 31.4 |
| 33 | 48 | 24.4 |
| 33 | 72 | 16.6 |
| 33 | 96 | 11.6 |
| 34 | 0.1667 | 238 |
| 34 | 0.5 | 807 |
| 34 | 1 | 1740 |
| 34 | 2 | 2350 |
| 34 | 4 | 1250 |
| 34 | 6 | 618 |
| 34 | 8 | 848 |
| 34 | 10 | 324 |
| 34 | 12 | 185 |
| 34 | 24 | 112.4 |
| 34 | 36 | 37.6 |
| 34 | 48 | 29.8 |
| 34 | 72 | 24.6 |
| 34 | 96 | 7.8 |
| 35 | 0.1667 | 161 |
| 35 | 0.5 | 639 |
| 35 | 1 | 1170 |
| 35 | 2 | 2180 |
| 35 | 4 | 1100 |
| 35 | 6 | 498 |
| 35 | 8 | 448 |
| 35 | 10 | 310 |
| 35 | 12 | 120 |
| 35 | 24 | 88.8 |
| 35 | 36 | 29.6 |
| 35 | 48 | 25.6 |
| 35 | 72 | 14.8 |
| 35 | 96 | 10.7 |
| 36 | 0.1667 | 195 |
| 36 | 0.5 | 642 |
| 36 | 1 | 1470 |
| 36 | 2 | 2360 |
| 36 | 4 | 1430 |
| 36 | 6 | 684 |
| 36 | 8 | 848 |
| 36 | 10 | 366 |
| 36 | 12 | 191 |
| 36 | 24 | 98.8 |
| 36 | 36 | 29 |
| 36 | 48 | 23 |
| 36 | 72 | 17 |
| 36 | 96 | 9.3 |
| 37 | 0.1667 | 169 |
| 37 | 0.5 | 714 |
| 37 | 1 | 1540 |
| 37 | 2 | 2010 |
| 37 | 4 | 1840 |
| 37 | 6 | 786 |
| 37 | 8 | 624 |
| 37 | 10 | 242 |
| 37 | 12 | 313 |
| 37 | 24 | 73.2 |
| 37 | 36 | 32.8 |
| 37 | 48 | 19.2 |
| 37 | 72 | 37.4 |
| 37 | 96 | 14.2 |
| 38 | 0.1667 | 159 |
| 38 | 0.5 | 864 |
| 38 | 1 | 1840 |
| 38 | 2 | 2570 |
| 38 | 4 | 1750 |
| 38 | 6 | 654 |
| 38 | 8 | 752 |
| 38 | 10 | 340 |
| 38 | 12 | 195 |
| 38 | 24 | 80.4 |
| 38 | 36 | 34.6 |
| 38 | 48 | 19 |
| 38 | 72 | 17 |
| 38 | 96 | 11.8 |
| 39 | 0.1667 | 217 |
| 39 | 0.5 | 636 |
| 39 | 1 | 1340 |
| 39 | 2 | 2200 |
| 39 | 4 | 1390 |
| 39 | 6 | 504 |
| 39 | 8 | 632 |
| 39 | 10 | 276 |
| 39 | 12 | 166 |
| 39 | 24 | 47.2 |
| 39 | 36 | 21 |
| 39 | 48 | 15.6 |
| 39 | 72 | 16.4 |
| 39 | 96 | 11.7 |
| 40 | 0.1667 | 205 |
| 40 | 0.5 | 699 |
| 40 | 1 | 1190 |
| 40 | 2 | 2470 |
| 40 | 4 | 1510 |
| 40 | 6 | 540 |
| 40 | 8 | 452 |
| 40 | 10 | 652 |
| 40 | 12 | 346 |
| 40 | 24 | 62 |
| 40 | 36 | 27.4 |
| 40 | 48 | 19.2 |
| 40 | 72 | 17.2 |
| 40 | 96 | 12.5 |
| 41 | 0.1667 | 169 |
| 41 | 0.5 | 642 |
| 41 | 1 | 1340 |
| 41 | 2 | 2730 |
| 41 | 4 | 1730 |
| 41 | 6 | 684 |
| 41 | 8 | 484 |
| 41 | 10 | 212 |
| 41 | 12 | 329 |
| 41 | 24 | 77.2 |
| 41 | 36 | 28.8 |
| 41 | 48 | 18.8 |
| 41 | 72 | 15.4 |
| 41 | 96 | 9.6 |
| 42 | 0.1667 | 149 |
| 42 | 0.5 | 540 |
| 42 | 1 | 1520 |
| 42 | 2 | 2750 |
| 42 | 4 | 1430 |
| 42 | 6 | 1128 |
| 42 | 8 | 812 |
| 42 | 10 | 372 |
| 42 | 12 | 386 |
| 42 | 24 | 75.2 |
| 42 | 36 | 35.2 |
| 42 | 48 | 29.6 |
| 42 | 72 | 19 |
| 42 | 96 | 14.1 |
| 43 | 0.1667 | 163 |
| 43 | 0.5 | 618 |
| 43 | 1 | 1650 |
| 43 | 2 | 2130 |
| 43 | 4 | 1460 |
| 43 | 6 | 1050 |
| 43 | 8 | 432 |
| 43 | 10 | 570 |
| 43 | 12 | 461 |
| 43 | 24 | 104.4 |
| 43 | 36 | 48.4 |
| 43 | 48 | 29.2 |
| 43 | 72 | 30 |
| 43 | 96 | 20.2 |
| 44 | 0.1667 | 172 |
| 44 | 0.5 | 885 |
| 44 | 1 | 1590 |
| 44 | 2 | 2000 |
| 44 | 4 | 1460 |
| 44 | 6 | 1056 |
| 44 | 8 | 672 |
| 44 | 10 | 492 |
| 44 | 12 | 388 |
| 44 | 24 | 94 |
| 44 | 36 | 35.8 |
| 44 | 48 | 26 |
| 44 | 72 | 22.2 |
| 44 | 96 | 15.9 |
| 45 | 0.1667 | 195 |
| 45 | 0.5 | 717 |
| 45 | 1 | 1790 |
| 45 | 2 | 2110 |
| 45 | 4 | 1830 |
| 45 | 6 | 1170 |
| 45 | 8 | 592 |
| 45 | 10 | 368 |
| 45 | 12 | 285 |
| 45 | 24 | 58 |
| 45 | 36 | 24.8 |
| 45 | 48 | 19 |
| 45 | 72 | 17 |
| 45 | 96 | 11.8 |
| 46 | 0.1667 | 186 |
| 46 | 0.5 | 1062 |
| 46 | 1 | 1260 |
| 46 | 2 | 1810 |
| 46 | 4 | 1560 |
| 46 | 6 | 582 |
| 46 | 8 | 400 |
| 46 | 10 | 288 |
| 46 | 12 | 178 |
| 46 | 24 | 56.4 |
| 46 | 36 | 25.4 |
| 46 | 48 | 18 |
| 46 | 72 | 14.2 |
| 46 | 96 | 7.6 |
| 47 | 0.1667 | 210 |
| 47 | 0.5 | 786 |
| 47 | 1 | 1700 |
| 47 | 2 | 2520 |
| 47 | 4 | 1320 |
| 47 | 6 | 630 |
| 47 | 8 | 752 |
| 47 | 10 | 362 |
| 47 | 12 | 253 |
| 47 | 24 | 73.2 |
| 47 | 36 | 30.6 |
| 47 | 48 | 20.4 |
| 47 | 72 | 15.4 |
| 47 | 96 | 11.5 |
| 48 | 0.1667 | 220 |
| 48 | 0.5 | 618 |
| 48 | 1 | 1430 |
| 48 | 2 | 1920 |
| 48 | 4 | 1450 |
| 48 | 6 | 666 |
| 48 | 8 | 340 |
| 48 | 10 | 380 |
| 48 | 12 | 290 |
| 48 | 24 | 94.8 |
| 48 | 36 | 31.6 |
| 48 | 48 | 20 |
| 48 | 72 | 17.8 |
| 48 | 96 | 8.4 |

| **Animal** | **Rsq** | **Rsq_adjusted** | **Corr_XY** | **No_points_lambda_z** | **Lambda_z (1/hr)** | **Lambda_z_lower (hr)** | **Lambda_z_upper (hr)** | **HL_Lambda_z (hr)** | **Tlag (hr)** | **Tmax (hr)** | **Cmax (ug/L)** | **Cmax_D (kg*ug/L/mg)** | **Tlast (hr)** | **Clast (ug/L)** | **AUClast (hr*ug/L)** | **AUCall (hr*ug/L)** | **AUCINF_obs (hr*ug/L)** | **AUCINF_D_obs (hr*kg*ug/L/mg)** | **AUC_%Extrap_obs (%)** | **Vz_F_obs (L/kg)** | **Cl_F_obs (L/hr/kg)** | **AUCINF_pred (hr*ug/L)** | **AUCINF_D_pred (hr*kg*ug/L/mg)** | **AUC_%Extrap_pred (%)** | **Vz_F_pred (L/kg)** | **Cl_F_pred (L/hr/kg)** | **AUMClast (hr*hr*ug/L)** | **AUMCINF_obs (hr*hr*ug/L)** | **AUMC_%Extrap_obs (%)** | **AUMCINF_pred (hr*hr*ug/L)** | **AUMC_%Extrap_pred (%)** | **MRTlast (hr)** | **MRTINF_obs (hr)** | **MRTINF_pred (hr)** |
| --- | --- | --- | --- | --- | --- | --- | --- | --- | --- | --- | --- | --- | --- | --- | --- | --- | --- | --- | --- | --- | --- | --- | --- | --- | --- | --- | --- | --- | --- | --- | --- | --- | --- | --- |
| 25 | 0.9616 | 0.9424 | -0.9806 | 4.0000 | 0.0141 | 36.0000 | 96.0000 | 49.0628 | 0.0000 | 2.0000 | 2220.0000 | 1044.7059 | 96.0000 | 14.2000 | 15024.9423 | 15024.9423 | 16030.0554 | 7543.5555 | 6.2702 | 9.3832 | 0.1326 | 15969.0798 | 7514.8611 | 5.9123 | 9.4190 | 0.1331 | 167391.3229 | 335026.7250 | 50.0364 | 324857.0607 | 48.4723 | 11.1409 | 20.8999 | 20.3429 |
| 26 | 0.9775 | 0.9662 | -0.9887 | 4.0000 | 0.0164 | 36.0000 | 96.0000 | 42.1593 | 0.0000 | 2.0000 | 2490.0000 | 1171.7647 | 96.0000 | 12.0000 | 15324.9379 | 15324.9379 | 16054.8134 | 7555.2063 | 4.5461 | 8.0505 | 0.1324 | 16043.7098 | 7549.9811 | 4.4801 | 8.0560 | 0.1325 | 169189.7209 | 283650.9504 | 40.3528 | 281909.6578 | 39.9844 | 11.0402 | 17.6677 | 17.5714 |
| 27 | 0.9423 | 0.9134 | -0.9707 | 4.0000 | 0.0175 | 36.0000 | 96.0000 | 39.7083 | 0.0000 | 2.0000 | 2750.0000 | 1294.1176 | 96.0000 | 12.6000 | 15456.2384 | 15456.2384 | 16178.0542 | 7613.2020 | 4.4617 | 7.5247 | 0.1314 | 16111.3807 | 7581.8262 | 4.0663 | 7.5558 | 0.1319 | 170621.3702 | 281266.3465 | 39.3381 | 271046.1547 | 37.0508 | 11.0390 | 17.3857 | 16.8233 |
| 28 | 0.9998 | 0.9995 | -0.9999 | 3.0000 | 0.0149 | 48.0000 | 96.0000 | 46.4240 | 0.0000 | 2.0000 | 2570.0000 | 1209.4118 | 96.0000 | 12.6000 | 18197.5364 | 18197.5364 | 19041.4304 | 8960.6731 | 4.4319 | 7.4744 | 0.1116 | 19044.0747 | 8961.9175 | 4.4452 | 7.4734 | 0.1116 | 210120.7862 | 347655.0161 | 39.5605 | 348085.9859 | 39.6354 | 11.5467 | 18.2578 | 18.2779 |
| 29 | 0.9899 | 0.9849 | -0.9949 | 4.0000 | 0.0217 | 36.0000 | 96.0000 | 31.9548 | 0.0000 | 2.0000 | 2210.0000 | 1040.0000 | 96.0000 | 11.4000 | 17847.7361 | 17847.7361 | 18373.2884 | 8646.2534 | 2.8604 | 5.3319 | 0.1157 | 18393.9098 | 8655.9576 | 2.9693 | 5.3259 | 0.1155 | 212438.1199 | 287119.6759 | 26.0106 | 290050.0040 | 26.7581 | 11.9028 | 15.6270 | 15.7688 |
| 30 | 0.8230 | 0.7876 | -0.9072 | 7.0000 | 0.0368 | 10.0000 | 96.0000 | 18.8274 | 0.0000 | 2.0000 | 2100.0000 | 988.2353 | 96.0000 | 14.5000 | 17023.3893 | 17023.3893 | 17417.2405 | 8196.3485 | 2.2613 | 3.3139 | 0.1220 | 17237.9495 | 8111.9762 | 1.2447 | 3.3484 | 0.1233 | 185846.8625 | 234354.4308 | 20.6984 | 212272.5656 | 12.4489 | 10.9171 | 13.4553 | 12.3143 |
| 31 | 0.7980 | 0.7728 | -0.8933 | 10.0000 | 0.0464 | 4.0000 | 96.0000 | 14.9509 | 0.0000 | 2.0000 | 2330.0000 | 1096.4706 | 96.0000 | 20.0000 | 15053.1366 | 15053.1366 | 15484.5282 | 7286.8368 | 2.7860 | 2.9601 | 0.1372 | 15194.6438 | 7150.4206 | 0.9313 | 3.0165 | 0.1399 | 179581.5866 | 230300.1126 | 22.0228 | 196218.5242 | 8.4788 | 11.9298 | 14.8729 | 12.9137 |
| 32 | 0.8700 | 0.8049 | -0.9327 | 4.0000 | 0.0143 | 36.0000 | 96.0000 | 48.3348 | 0.0000 | 1.0000 | 1760.0000 | 828.2353 | 96.0000 | 15.6000 | 13023.2346 | 13023.2346 | 14111.0587 | 6640.4982 | 7.7090 | 10.5011 | 0.1506 | 13983.8360 | 6580.6287 | 6.8694 | 10.5966 | 0.1520 | 161918.1857 | 342205.8037 | 52.6840 | 321120.8820 | 49.5772 | 12.4330 | 24.2509 | 22.9637 |
| 33 | 0.9996 | 0.9991 | -0.9998 | 3.0000 | 0.0155 | 48.0000 | 96.0000 | 44.7446 | 0.0000 | 2.0000 | 2380.0000 | 1120.0000 | 96.0000 | 11.6000 | 14259.3381 | 14259.3381 | 15008.1498 | 7062.6587 | 4.9894 | 9.1400 | 0.1416 | 15004.8147 | 7061.0893 | 4.9682 | 9.1420 | 0.1416 | 160199.6041 | 280423.3895 | 42.8722 | 279887.9298 | 42.7629 | 11.2347 | 18.6847 | 18.6532 |
| 34 | 0.9134 | 0.8918 | -0.9557 | 6.0000 | 0.0346 | 12.0000 | 96.0000 | 20.0425 | 0.0000 | 2.0000 | 2350.0000 | 1105.8824 | 96.0000 | 7.8000 | 15621.1366 | 15621.1366 | 15846.6753 | 7457.2590 | 1.4233 | 3.8775 | 0.1341 | 15844.2791 | 7456.1313 | 1.4083 | 3.8780 | 0.1341 | 187393.0369 | 215566.2661 | 13.0694 | 215266.9336 | 12.9485 | 11.9961 | 13.6032 | 13.5864 |
| 35 | 0.9869 | 0.9804 | -0.9934 | 4.0000 | 0.0177 | 36.0000 | 96.0000 | 39.1148 | 0.0000 | 2.0000 | 2180.0000 | 1025.8824 | 96.0000 | 10.7000 | 12371.1894 | 12371.1894 | 12974.9975 | 6105.8812 | 4.6536 | 9.2420 | 0.1638 | 12956.4363 | 6097.1465 | 4.5170 | 9.2553 | 0.1640 | 144162.1294 | 236201.0185 | 38.9663 | 233371.7162 | 38.2264 | 11.6531 | 18.2043 | 18.0120 |
| 36 | 0.9781 | 0.9672 | -0.9890 | 4.0000 | 0.0183 | 36.0000 | 96.0000 | 37.8859 | 0.0000 | 2.0000 | 2360.0000 | 1110.5882 | 96.0000 | 9.3000 | 15418.9393 | 15418.9393 | 15927.2563 | 7495.1794 | 3.1915 | 7.2924 | 0.1334 | 15957.4666 | 7509.3960 | 3.3748 | 7.2786 | 0.1332 | 165848.3713 | 242430.2556 | 31.5892 | 246981.6691 | 32.8499 | 10.7561 | 15.2211 | 15.4775 |
| 37 | 0.7358 | 0.7065 | -0.8578 | 11.0000 | 0.0499 | 2.0000 | 96.0000 | 13.9005 | 0.0000 | 2.0000 | 2010.0000 | 945.8824 | 96.0000 | 14.2000 | 16370.3381 | 16370.3381 | 16655.1079 | 7837.6978 | 1.7098 | 2.5587 | 0.1276 | 16490.2866 | 7760.1349 | 0.7274 | 2.5843 | 0.1289 | 205434.3871 | 238483.1239 | 13.8579 | 219354.9178 | 6.3461 | 12.5492 | 14.3189 | 13.3021 |
| 38 | 0.9135 | 0.8270 | -0.9558 | 3.0000 | 0.0099 | 48.0000 | 96.0000 | 69.8474 | 0.0000 | 2.0000 | 2570.0000 | 1209.4118 | 96.0000 | 11.8000 | 16263.3356 | 16263.3356 | 17452.4036 | 8212.8958 | 6.8132 | 12.2695 | 0.1218 | 17503.7984 | 8237.0816 | 7.0868 | 12.2335 | 0.1214 | 163824.6191 | 397795.7192 | 58.8169 | 407908.5878 | 59.8379 | 10.0732 | 22.7932 | 23.3040 |
| 39 | 0.7307 | 0.7008 | -0.8548 | 11.0000 | 0.0530 | 2.0000 | 96.0000 | 13.0726 | 0.0000 | 2.0000 | 2200.0000 | 1035.2941 | 96.0000 | 11.7000 | 13023.4394 | 13023.4394 | 13244.0993 | 6232.5173 | 1.6661 | 3.0260 | 0.1604 | 13091.4528 | 6160.6837 | 0.5195 | 3.0613 | 0.1623 | 132452.1382 | 157797.0948 | 16.0617 | 140264.1419 | 5.5695 | 10.1703 | 11.9145 | 10.7142 |
| 40 | 0.9178 | 0.8767 | -0.9580 | 4.0000 | 0.0117 | 36.0000 | 96.0000 | 59.4724 | 0.0000 | 2.0000 | 2470.0000 | 1162.3529 | 96.0000 | 12.5000 | 15651.1884 | 15651.1884 | 16723.6952 | 7869.9742 | 6.4131 | 10.9023 | 0.1271 | 16722.2818 | 7869.3091 | 6.4052 | 10.9032 | 0.1271 | 166755.8626 | 361738.2051 | 53.9015 | 361481.2455 | 53.8687 | 10.6545 | 21.6303 | 21.6167 |
| 41 | 0.9505 | 0.9258 | -0.9749 | 4.0000 | 0.0167 | 36.0000 | 96.0000 | 41.4828 | 0.0000 | 2.0000 | 2730.0000 | 1284.7059 | 96.0000 | 9.6000 | 16027.9393 | 16027.9393 | 16602.4705 | 7812.9273 | 3.4605 | 7.6600 | 0.1280 | 16607.9091 | 7815.4866 | 3.4921 | 7.6575 | 0.1280 | 159592.5877 | 249131.5590 | 35.9404 | 249979.1428 | 36.1576 | 9.9571 | 15.0057 | 15.0518 |
| 42 | 0.9931 | 0.9897 | -0.9966 | 4.0000 | 0.0156 | 36.0000 | 96.0000 | 44.4235 | 0.0000 | 2.0000 | 2750.0000 | 1294.1176 | 96.0000 | 14.1000 | 18196.0410 | 18196.0410 | 19099.7033 | 8988.0957 | 4.7313 | 7.1305 | 0.1113 | 19076.3164 | 8977.0901 | 4.6145 | 7.1392 | 0.1114 | 198854.3051 | 343521.1782 | 42.1129 | 339777.1730 | 41.4751 | 10.9284 | 17.9857 | 17.8115 |
| 43 | 0.7982 | 0.7758 | -0.8934 | 11.0000 | 0.0493 | 2.0000 | 96.0000 | 14.0647 | 0.0000 | 2.0000 | 2130.0000 | 1002.3529 | 96.0000 | 20.2000 | 18303.3397 | 18303.3397 | 18713.2207 | 8806.2215 | 2.1903 | 2.3042 | 0.1136 | 18458.7157 | 8686.4544 | 0.8417 | 2.3360 | 0.1151 | 240394.2379 | 288059.7690 | 16.5471 | 258463.0866 | 6.9909 | 13.1339 | 15.3934 | 14.0022 |
| 44 | 0.9515 | 0.9273 | -0.9755 | 4.0000 | 0.0124 | 36.0000 | 96.0000 | 56.0072 | 0.0000 | 2.0000 | 2000.0000 | 941.1765 | 96.0000 | 15.9000 | 17429.4353 | 17429.4353 | 18714.1772 | 8806.6716 | 6.8651 | 9.1750 | 0.1136 | 18715.3159 | 8807.2075 | 6.8707 | 9.1745 | 0.1135 | 206600.0357 | 433744.1811 | 52.3682 | 433945.5151 | 52.3903 | 11.8535 | 23.1773 | 23.1867 |
| 45 | 0.9465 | 0.9197 | -0.9729 | 4.0000 | 0.0113 | 36.0000 | 96.0000 | 61.5663 | 0.0000 | 2.0000 | 2110.0000 | 992.9412 | 96.0000 | 11.8000 | 16655.1881 | 16655.1881 | 17703.2805 | 8330.9555 | 5.9203 | 10.6616 | 0.1200 | 17729.2944 | 8343.1973 | 6.0584 | 10.6460 | 0.1199 | 164085.1957 | 357795.0911 | 54.1399 | 362603.0166 | 54.7480 | 9.8519 | 20.2107 | 20.4522 |
| 46 | 0.9655 | 0.9483 | -0.9826 | 4.0000 | 0.0188 | 36.0000 | 96.0000 | 36.9116 | 0.0000 | 2.0000 | 1810.0000 | 851.7647 | 96.0000 | 7.6000 | 12792.5823 | 12792.5823 | 13197.2984 | 6210.4934 | 3.0667 | 8.5745 | 0.1610 | 13219.6625 | 6221.0177 | 3.2306 | 8.5600 | 0.1607 | 129240.7927 | 189645.5341 | 31.8514 | 192983.4201 | 33.0301 | 10.1028 | 14.3700 | 14.5982 |
| 47 | 0.9999 | 0.9998 | -0.9999 | 3.0000 | 0.0119 | 48.0000 | 96.0000 | 58.0457 | 0.0000 | 2.0000 | 2520.0000 | 1185.8824 | 96.0000 | 11.5000 | 15454.3869 | 15454.3869 | 16417.4219 | 7725.8456 | 5.8659 | 10.8392 | 0.1294 | 16419.1655 | 7726.6661 | 5.8759 | 10.8381 | 0.1294 | 159437.0952 | 332535.1107 | 52.0541 | 332848.5019 | 52.0992 | 10.3166 | 20.2550 | 20.2719 |
| 48 | 0.9178 | 0.8767 | -0.9580 | 4.0000 | 0.0198 | 36.0000 | 96.0000 | 35.0311 | 0.0000 | 2.0000 | 1920.0000 | 903.5294 | 96.0000 | 8.4000 | 14371.7897 | 14371.7897 | 14796.3189 | 6962.9736 | 2.8692 | 7.2583 | 0.1436 | 14833.0074 | 6980.2388 | 3.1094 | 7.2403 | 0.1433 | 162631.2134 | 224841.3730 | 27.6685 | 230217.6853 | 29.3576 | 11.3160 | 15.1958 | 15.5206 |

| **Animal** | **Parameter** | **Units** | **Estimate** |
| --- | --- | --- | --- |
| 25 | Rsq |  | 0.9616 |
| 25 | Rsq_adjusted |  | 0.9424 |
| 25 | Corr_XY |  | -0.9806 |
| 25 | No_points_lambda_z |  | 4.0000 |
| 25 | Lambda_z | 1/hr | 0.0141 |
| 25 | Lambda_z_lower | hr | 36.0000 |
| 25 | Lambda_z_upper | hr | 96.0000 |
| 25 | HL_Lambda_z | hr | 49.0628 |
| 25 | Tlag | hr | 0.0000 |
| 25 | Tmax | hr | 2.0000 |
| 25 | Cmax | ug/L | 2220.0000 |
| 25 | Cmax_D | kg*ug/L/mg | 1044.7059 |
| 25 | Tlast | hr | 96.0000 |
| 25 | Clast | ug/L | 14.2000 |
| 25 | AUClast | hr*ug/L | 15024.9423 |
| 25 | AUCall | hr*ug/L | 15024.9423 |
| 25 | AUCINF_obs | hr*ug/L | 16030.0554 |
| 25 | AUCINF_D_obs | hr*kg*ug/L/mg | 7543.5555 |
| 25 | AUC_%Extrap_obs | % | 6.2702 |
| 25 | Vz_F_obs | L/kg | 9.3832 |
| 25 | Cl_F_obs | L/hr/kg | 0.1326 |
| 25 | AUCINF_pred | hr*ug/L | 15969.0798 |
| 25 | AUCINF_D_pred | hr*kg*ug/L/mg | 7514.8611 |
| 25 | AUC_%Extrap_pred | % | 5.9123 |
| 25 | Vz_F_pred | L/kg | 9.4190 |
| 25 | Cl_F_pred | L/hr/kg | 0.1331 |
| 25 | AUMClast | hr*hr*ug/L | 167391.3229 |
| 25 | AUMCINF_obs | hr*hr*ug/L | 335026.7250 |
| 25 | AUMC_%Extrap_obs | % | 50.0364 |
| 25 | AUMCINF_pred | hr*hr*ug/L | 324857.0607 |
| 25 | AUMC_%Extrap_pred | % | 48.4723 |
| 25 | MRTlast | hr | 11.1409 |
| 25 | MRTINF_obs | hr | 20.8999 |
| 25 | MRTINF_pred | hr | 20.3429 |
| 26 | Rsq |  | 0.9775 |
| 26 | Rsq_adjusted |  | 0.9662 |
| 26 | Corr_XY |  | -0.9887 |
| 26 | No_points_lambda_z |  | 4.0000 |
| 26 | Lambda_z | 1/hr | 0.0164 |
| 26 | Lambda_z_lower | hr | 36.0000 |
| 26 | Lambda_z_upper | hr | 96.0000 |
| 26 | HL_Lambda_z | hr | 42.1593 |
| 26 | Tlag | hr | 0.0000 |
| 26 | Tmax | hr | 2.0000 |
| 26 | Cmax | ug/L | 2490.0000 |
| 26 | Cmax_D | kg*ug/L/mg | 1171.7647 |
| 26 | Tlast | hr | 96.0000 |
| 26 | Clast | ug/L | 12.0000 |
| 26 | AUClast | hr*ug/L | 15324.9379 |
| 26 | AUCall | hr*ug/L | 15324.9379 |
| 26 | AUCINF_obs | hr*ug/L | 16054.8134 |
| 26 | AUCINF_D_obs | hr*kg*ug/L/mg | 7555.2063 |
| 26 | AUC_%Extrap_obs | % | 4.5461 |
| 26 | Vz_F_obs | L/kg | 8.0505 |
| 26 | Cl_F_obs | L/hr/kg | 0.1324 |
| 26 | AUCINF_pred | hr*ug/L | 16043.7098 |
| 26 | AUCINF_D_pred | hr*kg*ug/L/mg | 7549.9811 |
| 26 | AUC_%Extrap_pred | % | 4.4801 |
| 26 | Vz_F_pred | L/kg | 8.0560 |
| 26 | Cl_F_pred | L/hr/kg | 0.1325 |
| 26 | AUMClast | hr*hr*ug/L | 169189.7209 |
| 26 | AUMCINF_obs | hr*hr*ug/L | 283650.9504 |
| 26 | AUMC_%Extrap_obs | % | 40.3528 |
| 26 | AUMCINF_pred | hr*hr*ug/L | 281909.6578 |
| 26 | AUMC_%Extrap_pred | % | 39.9844 |
| 26 | MRTlast | hr | 11.0402 |
| 26 | MRTINF_obs | hr | 17.6677 |
| 26 | MRTINF_pred | hr | 17.5714 |
| 27 | Rsq |  | 0.9423 |
| 27 | Rsq_adjusted |  | 0.9134 |
| 27 | Corr_XY |  | -0.9707 |
| 27 | No_points_lambda_z |  | 4.0000 |
| 27 | Lambda_z | 1/hr | 0.0175 |
| 27 | Lambda_z_lower | hr | 36.0000 |
| 27 | Lambda_z_upper | hr | 96.0000 |
| 27 | HL_Lambda_z | hr | 39.7083 |
| 27 | Tlag | hr | 0.0000 |
| 27 | Tmax | hr | 2.0000 |
| 27 | Cmax | ug/L | 2750.0000 |
| 27 | Cmax_D | kg*ug/L/mg | 1294.1176 |
| 27 | Tlast | hr | 96.0000 |
| 27 | Clast | ug/L | 12.6000 |
| 27 | AUClast | hr*ug/L | 15456.2384 |
| 27 | AUCall | hr*ug/L | 15456.2384 |
| 27 | AUCINF_obs | hr*ug/L | 16178.0542 |
| 27 | AUCINF_D_obs | hr*kg*ug/L/mg | 7613.2020 |
| 27 | AUC_%Extrap_obs | % | 4.4617 |
| 27 | Vz_F_obs | L/kg | 7.5247 |
| 27 | Cl_F_obs | L/hr/kg | 0.1314 |
| 27 | AUCINF_pred | hr*ug/L | 16111.3807 |
| 27 | AUCINF_D_pred | hr*kg*ug/L/mg | 7581.8262 |
| 27 | AUC_%Extrap_pred | % | 4.0663 |
| 27 | Vz_F_pred | L/kg | 7.5558 |
| 27 | Cl_F_pred | L/hr/kg | 0.1319 |
| 27 | AUMClast | hr*hr*ug/L | 170621.3702 |
| 27 | AUMCINF_obs | hr*hr*ug/L | 281266.3465 |
| 27 | AUMC_%Extrap_obs | % | 39.3381 |
| 27 | AUMCINF_pred | hr*hr*ug/L | 271046.1547 |
| 27 | AUMC_%Extrap_pred | % | 37.0508 |
| 27 | MRTlast | hr | 11.0390 |
| 27 | MRTINF_obs | hr | 17.3857 |
| 27 | MRTINF_pred | hr | 16.8233 |
| 28 | Rsq |  | 0.9998 |
| 28 | Rsq_adjusted |  | 0.9995 |
| 28 | Corr_XY |  | -0.9999 |
| 28 | No_points_lambda_z |  | 3.0000 |
| 28 | Lambda_z | 1/hr | 0.0149 |
| 28 | Lambda_z_lower | hr | 48.0000 |
| 28 | Lambda_z_upper | hr | 96.0000 |
| 28 | HL_Lambda_z | hr | 46.4240 |
| 28 | Tlag | hr | 0.0000 |
| 28 | Tmax | hr | 2.0000 |
| 28 | Cmax | ug/L | 2570.0000 |
| 28 | Cmax_D | kg*ug/L/mg | 1209.4118 |
| 28 | Tlast | hr | 96.0000 |
| 28 | Clast | ug/L | 12.6000 |
| 28 | AUClast | hr*ug/L | 18197.5364 |
| 28 | AUCall | hr*ug/L | 18197.5364 |
| 28 | AUCINF_obs | hr*ug/L | 19041.4304 |
| 28 | AUCINF_D_obs | hr*kg*ug/L/mg | 8960.6731 |
| 28 | AUC_%Extrap_obs | % | 4.4319 |
| 28 | Vz_F_obs | L/kg | 7.4744 |
| 28 | Cl_F_obs | L/hr/kg | 0.1116 |
| 28 | AUCINF_pred | hr*ug/L | 19044.0747 |
| 28 | AUCINF_D_pred | hr*kg*ug/L/mg | 8961.9175 |
| 28 | AUC_%Extrap_pred | % | 4.4452 |
| 28 | Vz_F_pred | L/kg | 7.4734 |
| 28 | Cl_F_pred | L/hr/kg | 0.1116 |
| 28 | AUMClast | hr*hr*ug/L | 210120.7862 |
| 28 | AUMCINF_obs | hr*hr*ug/L | 347655.0161 |
| 28 | AUMC_%Extrap_obs | % | 39.5605 |
| 28 | AUMCINF_pred | hr*hr*ug/L | 348085.9859 |
| 28 | AUMC_%Extrap_pred | % | 39.6354 |
| 28 | MRTlast | hr | 11.5467 |
| 28 | MRTINF_obs | hr | 18.2578 |
| 28 | MRTINF_pred | hr | 18.2779 |
| 29 | Rsq |  | 0.9899 |
| 29 | Rsq_adjusted |  | 0.9849 |
| 29 | Corr_XY |  | -0.9949 |
| 29 | No_points_lambda_z |  | 4.0000 |
| 29 | Lambda_z | 1/hr | 0.0217 |
| 29 | Lambda_z_lower | hr | 36.0000 |
| 29 | Lambda_z_upper | hr | 96.0000 |
| 29 | HL_Lambda_z | hr | 31.9548 |
| 29 | Tlag | hr | 0.0000 |
| 29 | Tmax | hr | 2.0000 |
| 29 | Cmax | ug/L | 2210.0000 |
| 29 | Cmax_D | kg*ug/L/mg | 1040.0000 |
| 29 | Tlast | hr | 96.0000 |
| 29 | Clast | ug/L | 11.4000 |
| 29 | AUClast | hr*ug/L | 17847.7361 |
| 29 | AUCall | hr*ug/L | 17847.7361 |
| 29 | AUCINF_obs | hr*ug/L | 18373.2884 |
| 29 | AUCINF_D_obs | hr*kg*ug/L/mg | 8646.2534 |
| 29 | AUC_%Extrap_obs | % | 2.8604 |
| 29 | Vz_F_obs | L/kg | 5.3319 |
| 29 | Cl_F_obs | L/hr/kg | 0.1157 |
| 29 | AUCINF_pred | hr*ug/L | 18393.9098 |
| 29 | AUCINF_D_pred | hr*kg*ug/L/mg | 8655.9576 |
| 29 | AUC_%Extrap_pred | % | 2.9693 |
| 29 | Vz_F_pred | L/kg | 5.3259 |
| 29 | Cl_F_pred | L/hr/kg | 0.1155 |
| 29 | AUMClast | hr*hr*ug/L | 212438.1199 |
| 29 | AUMCINF_obs | hr*hr*ug/L | 287119.6759 |
| 29 | AUMC_%Extrap_obs | % | 26.0106 |
| 29 | AUMCINF_pred | hr*hr*ug/L | 290050.0040 |
| 29 | AUMC_%Extrap_pred | % | 26.7581 |
| 29 | MRTlast | hr | 11.9028 |
| 29 | MRTINF_obs | hr | 15.6270 |
| 29 | MRTINF_pred | hr | 15.7688 |
| 30 | Rsq |  | 0.8230 |
| 30 | Rsq_adjusted |  | 0.7876 |
| 30 | Corr_XY |  | -0.9072 |
| 30 | No_points_lambda_z |  | 7.0000 |
| 30 | Lambda_z | 1/hr | 0.0368 |
| 30 | Lambda_z_lower | hr | 10.0000 |
| 30 | Lambda_z_upper | hr | 96.0000 |
| 30 | HL_Lambda_z | hr | 18.8274 |
| 30 | Tlag | hr | 0.0000 |
| 30 | Tmax | hr | 2.0000 |
| 30 | Cmax | ug/L | 2100.0000 |
| 30 | Cmax_D | kg*ug/L/mg | 988.2353 |
| 30 | Tlast | hr | 96.0000 |
| 30 | Clast | ug/L | 14.5000 |
| 30 | AUClast | hr*ug/L | 17023.3893 |
| 30 | AUCall | hr*ug/L | 17023.3893 |
| 30 | AUCINF_obs | hr*ug/L | 17417.2405 |
| 30 | AUCINF_D_obs | hr*kg*ug/L/mg | 8196.3485 |
| 30 | AUC_%Extrap_obs | % | 2.2613 |
| 30 | Vz_F_obs | L/kg | 3.3139 |
| 30 | Cl_F_obs | L/hr/kg | 0.1220 |
| 30 | AUCINF_pred | hr*ug/L | 17237.9495 |
| 30 | AUCINF_D_pred | hr*kg*ug/L/mg | 8111.9762 |
| 30 | AUC_%Extrap_pred | % | 1.2447 |
| 30 | Vz_F_pred | L/kg | 3.3484 |
| 30 | Cl_F_pred | L/hr/kg | 0.1233 |
| 30 | AUMClast | hr*hr*ug/L | 185846.8625 |
| 30 | AUMCINF_obs | hr*hr*ug/L | 234354.4308 |
| 30 | AUMC_%Extrap_obs | % | 20.6984 |
| 30 | AUMCINF_pred | hr*hr*ug/L | 212272.5656 |
| 30 | AUMC_%Extrap_pred | % | 12.4489 |
| 30 | MRTlast | hr | 10.9171 |
| 30 | MRTINF_obs | hr | 13.4553 |
| 30 | MRTINF_pred | hr | 12.3143 |
| 31 | Rsq |  | 0.7980 |
| 31 | Rsq_adjusted |  | 0.7728 |
| 31 | Corr_XY |  | -0.8933 |
| 31 | No_points_lambda_z |  | 10.0000 |
| 31 | Lambda_z | 1/hr | 0.0464 |
| 31 | Lambda_z_lower | hr | 4.0000 |
| 31 | Lambda_z_upper | hr | 96.0000 |
| 31 | HL_Lambda_z | hr | 14.9509 |
| 31 | Tlag | hr | 0.0000 |
| 31 | Tmax | hr | 2.0000 |
| 31 | Cmax | ug/L | 2330.0000 |
| 31 | Cmax_D | kg*ug/L/mg | 1096.4706 |
| 31 | Tlast | hr | 96.0000 |
| 31 | Clast | ug/L | 20.0000 |
| 31 | AUClast | hr*ug/L | 15053.1366 |
| 31 | AUCall | hr*ug/L | 15053.1366 |
| 31 | AUCINF_obs | hr*ug/L | 15484.5282 |
| 31 | AUCINF_D_obs | hr*kg*ug/L/mg | 7286.8368 |
| 31 | AUC_%Extrap_obs | % | 2.7860 |
| 31 | Vz_F_obs | L/kg | 2.9601 |
| 31 | Cl_F_obs | L/hr/kg | 0.1372 |
| 31 | AUCINF_pred | hr*ug/L | 15194.6438 |
| 31 | AUCINF_D_pred | hr*kg*ug/L/mg | 7150.4206 |
| 31 | AUC_%Extrap_pred | % | 0.9313 |
| 31 | Vz_F_pred | L/kg | 3.0165 |
| 31 | Cl_F_pred | L/hr/kg | 0.1399 |
| 31 | AUMClast | hr*hr*ug/L | 179581.5866 |
| 31 | AUMCINF_obs | hr*hr*ug/L | 230300.1126 |
| 31 | AUMC_%Extrap_obs | % | 22.0228 |
| 31 | AUMCINF_pred | hr*hr*ug/L | 196218.5242 |
| 31 | AUMC_%Extrap_pred | % | 8.4788 |
| 31 | MRTlast | hr | 11.9298 |
| 31 | MRTINF_obs | hr | 14.8729 |
| 31 | MRTINF_pred | hr | 12.9137 |
| 32 | Rsq |  | 0.8700 |
| 32 | Rsq_adjusted |  | 0.8049 |
| 32 | Corr_XY |  | -0.9327 |
| 32 | No_points_lambda_z |  | 4.0000 |
| 32 | Lambda_z | 1/hr | 0.0143 |
| 32 | Lambda_z_lower | hr | 36.0000 |
| 32 | Lambda_z_upper | hr | 96.0000 |
| 32 | HL_Lambda_z | hr | 48.3348 |
| 32 | Tlag | hr | 0.0000 |
| 32 | Tmax | hr | 1.0000 |
| 32 | Cmax | ug/L | 1760.0000 |
| 32 | Cmax_D | kg*ug/L/mg | 828.2353 |
| 32 | Tlast | hr | 96.0000 |
| 32 | Clast | ug/L | 15.6000 |
| 32 | AUClast | hr*ug/L | 13023.2346 |
| 32 | AUCall | hr*ug/L | 13023.2346 |
| 32 | AUCINF_obs | hr*ug/L | 14111.0587 |
| 32 | AUCINF_D_obs | hr*kg*ug/L/mg | 6640.4982 |
| 32 | AUC_%Extrap_obs | % | 7.7090 |
| 32 | Vz_F_obs | L/kg | 10.5011 |
| 32 | Cl_F_obs | L/hr/kg | 0.1506 |
| 32 | AUCINF_pred | hr*ug/L | 13983.8360 |
| 32 | AUCINF_D_pred | hr*kg*ug/L/mg | 6580.6287 |
| 32 | AUC_%Extrap_pred | % | 6.8694 |
| 32 | Vz_F_pred | L/kg | 10.5966 |
| 32 | Cl_F_pred | L/hr/kg | 0.1520 |
| 32 | AUMClast | hr*hr*ug/L | 161918.1857 |
| 32 | AUMCINF_obs | hr*hr*ug/L | 342205.8037 |
| 32 | AUMC_%Extrap_obs | % | 52.6840 |
| 32 | AUMCINF_pred | hr*hr*ug/L | 321120.8820 |
| 32 | AUMC_%Extrap_pred | % | 49.5772 |
| 32 | MRTlast | hr | 12.4330 |
| 32 | MRTINF_obs | hr | 24.2509 |
| 32 | MRTINF_pred | hr | 22.9637 |
| 33 | Rsq |  | 0.9996 |
| 33 | Rsq_adjusted |  | 0.9991 |
| 33 | Corr_XY |  | -0.9998 |
| 33 | No_points_lambda_z |  | 3.0000 |
| 33 | Lambda_z | 1/hr | 0.0155 |
| 33 | Lambda_z_lower | hr | 48.0000 |
| 33 | Lambda_z_upper | hr | 96.0000 |
| 33 | HL_Lambda_z | hr | 44.7446 |
| 33 | Tlag | hr | 0.0000 |
| 33 | Tmax | hr | 2.0000 |
| 33 | Cmax | ug/L | 2380.0000 |
| 33 | Cmax_D | kg*ug/L/mg | 1120.0000 |
| 33 | Tlast | hr | 96.0000 |
| 33 | Clast | ug/L | 11.6000 |
| 33 | AUClast | hr*ug/L | 14259.3381 |
| 33 | AUCall | hr*ug/L | 14259.3381 |
| 33 | AUCINF_obs | hr*ug/L | 15008.1498 |
| 33 | AUCINF_D_obs | hr*kg*ug/L/mg | 7062.6587 |
| 33 | AUC_%Extrap_obs | % | 4.9894 |
| 33 | Vz_F_obs | L/kg | 9.1400 |
| 33 | Cl_F_obs | L/hr/kg | 0.1416 |
| 33 | AUCINF_pred | hr*ug/L | 15004.8147 |
| 33 | AUCINF_D_pred | hr*kg*ug/L/mg | 7061.0893 |
| 33 | AUC_%Extrap_pred | % | 4.9682 |
| 33 | Vz_F_pred | L/kg | 9.1420 |
| 33 | Cl_F_pred | L/hr/kg | 0.1416 |
| 33 | AUMClast | hr*hr*ug/L | 160199.6041 |
| 33 | AUMCINF_obs | hr*hr*ug/L | 280423.3895 |
| 33 | AUMC_%Extrap_obs | % | 42.8722 |
| 33 | AUMCINF_pred | hr*hr*ug/L | 279887.9298 |
| 33 | AUMC_%Extrap_pred | % | 42.7629 |
| 33 | MRTlast | hr | 11.2347 |
| 33 | MRTINF_obs | hr | 18.6847 |
| 33 | MRTINF_pred | hr | 18.6532 |
| 34 | Rsq |  | 0.9134 |
| 34 | Rsq_adjusted |  | 0.8918 |
| 34 | Corr_XY |  | -0.9557 |
| 34 | No_points_lambda_z |  | 6.0000 |
| 34 | Lambda_z | 1/hr | 0.0346 |
| 34 | Lambda_z_lower | hr | 12.0000 |
| 34 | Lambda_z_upper | hr | 96.0000 |
| 34 | HL_Lambda_z | hr | 20.0425 |
| 34 | Tlag | hr | 0.0000 |
| 34 | Tmax | hr | 2.0000 |
| 34 | Cmax | ug/L | 2350.0000 |
| 34 | Cmax_D | kg*ug/L/mg | 1105.8824 |
| 34 | Tlast | hr | 96.0000 |
| 34 | Clast | ug/L | 7.8000 |
| 34 | AUClast | hr*ug/L | 15621.1366 |
| 34 | AUCall | hr*ug/L | 15621.1366 |
| 34 | AUCINF_obs | hr*ug/L | 15846.6753 |
| 34 | AUCINF_D_obs | hr*kg*ug/L/mg | 7457.2590 |
| 34 | AUC_%Extrap_obs | % | 1.4233 |
| 34 | Vz_F_obs | L/kg | 3.8775 |
| 34 | Cl_F_obs | L/hr/kg | 0.1341 |
| 34 | AUCINF_pred | hr*ug/L | 15844.2791 |
| 34 | AUCINF_D_pred | hr*kg*ug/L/mg | 7456.1313 |
| 34 | AUC_%Extrap_pred | % | 1.4083 |
| 34 | Vz_F_pred | L/kg | 3.8780 |
| 34 | Cl_F_pred | L/hr/kg | 0.1341 |
| 34 | AUMClast | hr*hr*ug/L | 187393.0369 |
| 34 | AUMCINF_obs | hr*hr*ug/L | 215566.2661 |
| 34 | AUMC_%Extrap_obs | % | 13.0694 |
| 34 | AUMCINF_pred | hr*hr*ug/L | 215266.9336 |
| 34 | AUMC_%Extrap_pred | % | 12.9485 |
| 34 | MRTlast | hr | 11.9961 |
| 34 | MRTINF_obs | hr | 13.6032 |
| 34 | MRTINF_pred | hr | 13.5864 |
| 35 | Rsq |  | 0.9869 |
| 35 | Rsq_adjusted |  | 0.9804 |
| 35 | Corr_XY |  | -0.9934 |
| 35 | No_points_lambda_z |  | 4.0000 |
| 35 | Lambda_z | 1/hr | 0.0177 |
| 35 | Lambda_z_lower | hr | 36.0000 |
| 35 | Lambda_z_upper | hr | 96.0000 |
| 35 | HL_Lambda_z | hr | 39.1148 |
| 35 | Tlag | hr | 0.0000 |
| 35 | Tmax | hr | 2.0000 |
| 35 | Cmax | ug/L | 2180.0000 |
| 35 | Cmax_D | kg*ug/L/mg | 1025.8824 |
| 35 | Tlast | hr | 96.0000 |
| 35 | Clast | ug/L | 10.7000 |
| 35 | AUClast | hr*ug/L | 12371.1894 |
| 35 | AUCall | hr*ug/L | 12371.1894 |
| 35 | AUCINF_obs | hr*ug/L | 12974.9975 |
| 35 | AUCINF_D_obs | hr*kg*ug/L/mg | 6105.8812 |
| 35 | AUC_%Extrap_obs | % | 4.6536 |
| 35 | Vz_F_obs | L/kg | 9.2420 |
| 35 | Cl_F_obs | L/hr/kg | 0.1638 |
| 35 | AUCINF_pred | hr*ug/L | 12956.4363 |
| 35 | AUCINF_D_pred | hr*kg*ug/L/mg | 6097.1465 |
| 35 | AUC_%Extrap_pred | % | 4.5170 |
| 35 | Vz_F_pred | L/kg | 9.2553 |
| 35 | Cl_F_pred | L/hr/kg | 0.1640 |
| 35 | AUMClast | hr*hr*ug/L | 144162.1294 |
| 35 | AUMCINF_obs | hr*hr*ug/L | 236201.0185 |
| 35 | AUMC_%Extrap_obs | % | 38.9663 |
| 35 | AUMCINF_pred | hr*hr*ug/L | 233371.7162 |
| 35 | AUMC_%Extrap_pred | % | 38.2264 |
| 35 | MRTlast | hr | 11.6531 |
| 35 | MRTINF_obs | hr | 18.2043 |
| 35 | MRTINF_pred | hr | 18.0120 |
| 36 | Rsq |  | 0.9781 |
| 36 | Rsq_adjusted |  | 0.9672 |
| 36 | Corr_XY |  | -0.9890 |
| 36 | No_points_lambda_z |  | 4.0000 |
| 36 | Lambda_z | 1/hr | 0.0183 |
| 36 | Lambda_z_lower | hr | 36.0000 |
| 36 | Lambda_z_upper | hr | 96.0000 |
| 36 | HL_Lambda_z | hr | 37.8859 |
| 36 | Tlag | hr | 0.0000 |
| 36 | Tmax | hr | 2.0000 |
| 36 | Cmax | ug/L | 2360.0000 |
| 36 | Cmax_D | kg*ug/L/mg | 1110.5882 |
| 36 | Tlast | hr | 96.0000 |
| 36 | Clast | ug/L | 9.3000 |
| 36 | AUClast | hr*ug/L | 15418.9393 |
| 36 | AUCall | hr*ug/L | 15418.9393 |
| 36 | AUCINF_obs | hr*ug/L | 15927.2563 |
| 36 | AUCINF_D_obs | hr*kg*ug/L/mg | 7495.1794 |
| 36 | AUC_%Extrap_obs | % | 3.1915 |
| 36 | Vz_F_obs | L/kg | 7.2924 |
| 36 | Cl_F_obs | L/hr/kg | 0.1334 |
| 36 | AUCINF_pred | hr*ug/L | 15957.4666 |
| 36 | AUCINF_D_pred | hr*kg*ug/L/mg | 7509.3960 |
| 36 | AUC_%Extrap_pred | % | 3.3748 |
| 36 | Vz_F_pred | L/kg | 7.2786 |
| 36 | Cl_F_pred | L/hr/kg | 0.1332 |
| 36 | AUMClast | hr*hr*ug/L | 165848.3713 |
| 36 | AUMCINF_obs | hr*hr*ug/L | 242430.2556 |
| 36 | AUMC_%Extrap_obs | % | 31.5892 |
| 36 | AUMCINF_pred | hr*hr*ug/L | 246981.6691 |
| 36 | AUMC_%Extrap_pred | % | 32.8499 |
| 36 | MRTlast | hr | 10.7561 |
| 36 | MRTINF_obs | hr | 15.2211 |
| 36 | MRTINF_pred | hr | 15.4775 |
| 37 | Rsq |  | 0.7358 |
| 37 | Rsq_adjusted |  | 0.7065 |
| 37 | Corr_XY |  | -0.8578 |
| 37 | No_points_lambda_z |  | 11.0000 |
| 37 | Lambda_z | 1/hr | 0.0499 |
| 37 | Lambda_z_lower | hr | 2.0000 |
| 37 | Lambda_z_upper | hr | 96.0000 |
| 37 | HL_Lambda_z | hr | 13.9005 |
| 37 | Tlag | hr | 0.0000 |
| 37 | Tmax | hr | 2.0000 |
| 37 | Cmax | ug/L | 2010.0000 |
| 37 | Cmax_D | kg*ug/L/mg | 945.8824 |
| 37 | Tlast | hr | 96.0000 |
| 37 | Clast | ug/L | 14.2000 |
| 37 | AUClast | hr*ug/L | 16370.3381 |
| 37 | AUCall | hr*ug/L | 16370.3381 |
| 37 | AUCINF_obs | hr*ug/L | 16655.1079 |
| 37 | AUCINF_D_obs | hr*kg*ug/L/mg | 7837.6978 |
| 37 | AUC_%Extrap_obs | % | 1.7098 |
| 37 | Vz_F_obs | L/kg | 2.5587 |
| 37 | Cl_F_obs | L/hr/kg | 0.1276 |
| 37 | AUCINF_pred | hr*ug/L | 16490.2866 |
| 37 | AUCINF_D_pred | hr*kg*ug/L/mg | 7760.1349 |
| 37 | AUC_%Extrap_pred | % | 0.7274 |
| 37 | Vz_F_pred | L/kg | 2.5843 |
| 37 | Cl_F_pred | L/hr/kg | 0.1289 |
| 37 | AUMClast | hr*hr*ug/L | 205434.3871 |
| 37 | AUMCINF_obs | hr*hr*ug/L | 238483.1239 |
| 37 | AUMC_%Extrap_obs | % | 13.8579 |
| 37 | AUMCINF_pred | hr*hr*ug/L | 219354.9178 |
| 37 | AUMC_%Extrap_pred | % | 6.3461 |
| 37 | MRTlast | hr | 12.5492 |
| 37 | MRTINF_obs | hr | 14.3189 |
| 37 | MRTINF_pred | hr | 13.3021 |
| 38 | Rsq |  | 0.9135 |
| 38 | Rsq_adjusted |  | 0.8270 |
| 38 | Corr_XY |  | -0.9558 |
| 38 | No_points_lambda_z |  | 3.0000 |
| 38 | Lambda_z | 1/hr | 0.0099 |
| 38 | Lambda_z_lower | hr | 48.0000 |
| 38 | Lambda_z_upper | hr | 96.0000 |
| 38 | HL_Lambda_z | hr | 69.8474 |
| 38 | Tlag | hr | 0.0000 |
| 38 | Tmax | hr | 2.0000 |
| 38 | Cmax | ug/L | 2570.0000 |
| 38 | Cmax_D | kg*ug/L/mg | 1209.4118 |
| 38 | Tlast | hr | 96.0000 |
| 38 | Clast | ug/L | 11.8000 |
| 38 | AUClast | hr*ug/L | 16263.3356 |
| 38 | AUCall | hr*ug/L | 16263.3356 |
| 38 | AUCINF_obs | hr*ug/L | 17452.4036 |
| 38 | AUCINF_D_obs | hr*kg*ug/L/mg | 8212.8958 |
| 38 | AUC_%Extrap_obs | % | 6.8132 |
| 38 | Vz_F_obs | L/kg | 12.2695 |
| 38 | Cl_F_obs | L/hr/kg | 0.1218 |
| 38 | AUCINF_pred | hr*ug/L | 17503.7984 |
| 38 | AUCINF_D_pred | hr*kg*ug/L/mg | 8237.0816 |
| 38 | AUC_%Extrap_pred | % | 7.0868 |
| 38 | Vz_F_pred | L/kg | 12.2335 |
| 38 | Cl_F_pred | L/hr/kg | 0.1214 |
| 38 | AUMClast | hr*hr*ug/L | 163824.6191 |
| 38 | AUMCINF_obs | hr*hr*ug/L | 397795.7192 |
| 38 | AUMC_%Extrap_obs | % | 58.8169 |
| 38 | AUMCINF_pred | hr*hr*ug/L | 407908.5878 |
| 38 | AUMC_%Extrap_pred | % | 59.8379 |
| 38 | MRTlast | hr | 10.0732 |
| 38 | MRTINF_obs | hr | 22.7932 |
| 38 | MRTINF_pred | hr | 23.3040 |
| 39 | Rsq |  | 0.7307 |
| 39 | Rsq_adjusted |  | 0.7008 |
| 39 | Corr_XY |  | -0.8548 |
| 39 | No_points_lambda_z |  | 11.0000 |
| 39 | Lambda_z | 1/hr | 0.0530 |
| 39 | Lambda_z_lower | hr | 2.0000 |
| 39 | Lambda_z_upper | hr | 96.0000 |
| 39 | HL_Lambda_z | hr | 13.0726 |
| 39 | Tlag | hr | 0.0000 |
| 39 | Tmax | hr | 2.0000 |
| 39 | Cmax | ug/L | 2200.0000 |
| 39 | Cmax_D | kg*ug/L/mg | 1035.2941 |
| 39 | Tlast | hr | 96.0000 |
| 39 | Clast | ug/L | 11.7000 |
| 39 | AUClast | hr*ug/L | 13023.4394 |
| 39 | AUCall | hr*ug/L | 13023.4394 |
| 39 | AUCINF_obs | hr*ug/L | 13244.0993 |
| 39 | AUCINF_D_obs | hr*kg*ug/L/mg | 6232.5173 |
| 39 | AUC_%Extrap_obs | % | 1.6661 |
| 39 | Vz_F_obs | L/kg | 3.0260 |
| 39 | Cl_F_obs | L/hr/kg | 0.1604 |
| 39 | AUCINF_pred | hr*ug/L | 13091.4528 |
| 39 | AUCINF_D_pred | hr*kg*ug/L/mg | 6160.6837 |
| 39 | AUC_%Extrap_pred | % | 0.5195 |
| 39 | Vz_F_pred | L/kg | 3.0613 |
| 39 | Cl_F_pred | L/hr/kg | 0.1623 |
| 39 | AUMClast | hr*hr*ug/L | 132452.1382 |
| 39 | AUMCINF_obs | hr*hr*ug/L | 157797.0948 |
| 39 | AUMC_%Extrap_obs | % | 16.0617 |
| 39 | AUMCINF_pred | hr*hr*ug/L | 140264.1419 |
| 39 | AUMC_%Extrap_pred | % | 5.5695 |
| 39 | MRTlast | hr | 10.1703 |
| 39 | MRTINF_obs | hr | 11.9145 |
| 39 | MRTINF_pred | hr | 10.7142 |
| 40 | Rsq |  | 0.9178 |
| 40 | Rsq_adjusted |  | 0.8767 |
| 40 | Corr_XY |  | -0.9580 |
| 40 | No_points_lambda_z |  | 4.0000 |
| 40 | Lambda_z | 1/hr | 0.0117 |
| 40 | Lambda_z_lower | hr | 36.0000 |
| 40 | Lambda_z_upper | hr | 96.0000 |
| 40 | HL_Lambda_z | hr | 59.4724 |
| 40 | Tlag | hr | 0.0000 |
| 40 | Tmax | hr | 2.0000 |
| 40 | Cmax | ug/L | 2470.0000 |
| 40 | Cmax_D | kg*ug/L/mg | 1162.3529 |
| 40 | Tlast | hr | 96.0000 |
| 40 | Clast | ug/L | 12.5000 |
| 40 | AUClast | hr*ug/L | 15651.1884 |
| 40 | AUCall | hr*ug/L | 15651.1884 |
| 40 | AUCINF_obs | hr*ug/L | 16723.6952 |
| 40 | AUCINF_D_obs | hr*kg*ug/L/mg | 7869.9742 |
| 40 | AUC_%Extrap_obs | % | 6.4131 |
| 40 | Vz_F_obs | L/kg | 10.9023 |
| 40 | Cl_F_obs | L/hr/kg | 0.1271 |
| 40 | AUCINF_pred | hr*ug/L | 16722.2818 |
| 40 | AUCINF_D_pred | hr*kg*ug/L/mg | 7869.3091 |
| 40 | AUC_%Extrap_pred | % | 6.4052 |
| 40 | Vz_F_pred | L/kg | 10.9032 |
| 40 | Cl_F_pred | L/hr/kg | 0.1271 |
| 40 | AUMClast | hr*hr*ug/L | 166755.8626 |
| 40 | AUMCINF_obs | hr*hr*ug/L | 361738.2051 |
| 40 | AUMC_%Extrap_obs | % | 53.9015 |
| 40 | AUMCINF_pred | hr*hr*ug/L | 361481.2455 |
| 40 | AUMC_%Extrap_pred | % | 53.8687 |
| 40 | MRTlast | hr | 10.6545 |
| 40 | MRTINF_obs | hr | 21.6303 |
| 40 | MRTINF_pred | hr | 21.6167 |
| 41 | Rsq |  | 0.9505 |
| 41 | Rsq_adjusted |  | 0.9258 |
| 41 | Corr_XY |  | -0.9749 |
| 41 | No_points_lambda_z |  | 4.0000 |
| 41 | Lambda_z | 1/hr | 0.0167 |
| 41 | Lambda_z_lower | hr | 36.0000 |
| 41 | Lambda_z_upper | hr | 96.0000 |
| 41 | HL_Lambda_z | hr | 41.4828 |
| 41 | Tlag | hr | 0.0000 |
| 41 | Tmax | hr | 2.0000 |
| 41 | Cmax | ug/L | 2730.0000 |
| 41 | Cmax_D | kg*ug/L/mg | 1284.7059 |
| 41 | Tlast | hr | 96.0000 |
| 41 | Clast | ug/L | 9.6000 |
| 41 | AUClast | hr*ug/L | 16027.9393 |
| 41 | AUCall | hr*ug/L | 16027.9393 |
| 41 | AUCINF_obs | hr*ug/L | 16602.4705 |
| 41 | AUCINF_D_obs | hr*kg*ug/L/mg | 7812.9273 |
| 41 | AUC_%Extrap_obs | % | 3.4605 |
| 41 | Vz_F_obs | L/kg | 7.6600 |
| 41 | Cl_F_obs | L/hr/kg | 0.1280 |
| 41 | AUCINF_pred | hr*ug/L | 16607.9091 |
| 41 | AUCINF_D_pred | hr*kg*ug/L/mg | 7815.4866 |
| 41 | AUC_%Extrap_pred | % | 3.4921 |
| 41 | Vz_F_pred | L/kg | 7.6575 |
| 41 | Cl_F_pred | L/hr/kg | 0.1280 |
| 41 | AUMClast | hr*hr*ug/L | 159592.5877 |
| 41 | AUMCINF_obs | hr*hr*ug/L | 249131.5590 |
| 41 | AUMC_%Extrap_obs | % | 35.9404 |
| 41 | AUMCINF_pred | hr*hr*ug/L | 249979.1428 |
| 41 | AUMC_%Extrap_pred | % | 36.1576 |
| 41 | MRTlast | hr | 9.9571 |
| 41 | MRTINF_obs | hr | 15.0057 |
| 41 | MRTINF_pred | hr | 15.0518 |
| 42 | Rsq |  | 0.9931 |
| 42 | Rsq_adjusted |  | 0.9897 |
| 42 | Corr_XY |  | -0.9966 |
| 42 | No_points_lambda_z |  | 4.0000 |
| 42 | Lambda_z | 1/hr | 0.0156 |
| 42 | Lambda_z_lower | hr | 36.0000 |
| 42 | Lambda_z_upper | hr | 96.0000 |
| 42 | HL_Lambda_z | hr | 44.4235 |
| 42 | Tlag | hr | 0.0000 |
| 42 | Tmax | hr | 2.0000 |
| 42 | Cmax | ug/L | 2750.0000 |
| 42 | Cmax_D | kg*ug/L/mg | 1294.1176 |
| 42 | Tlast | hr | 96.0000 |
| 42 | Clast | ug/L | 14.1000 |
| 42 | AUClast | hr*ug/L | 18196.0410 |
| 42 | AUCall | hr*ug/L | 18196.0410 |
| 42 | AUCINF_obs | hr*ug/L | 19099.7033 |
| 42 | AUCINF_D_obs | hr*kg*ug/L/mg | 8988.0957 |
| 42 | AUC_%Extrap_obs | % | 4.7313 |
| 42 | Vz_F_obs | L/kg | 7.1305 |
| 42 | Cl_F_obs | L/hr/kg | 0.1113 |
| 42 | AUCINF_pred | hr*ug/L | 19076.3164 |
| 42 | AUCINF_D_pred | hr*kg*ug/L/mg | 8977.0901 |
| 42 | AUC_%Extrap_pred | % | 4.6145 |
| 42 | Vz_F_pred | L/kg | 7.1392 |
| 42 | Cl_F_pred | L/hr/kg | 0.1114 |
| 42 | AUMClast | hr*hr*ug/L | 198854.3051 |
| 42 | AUMCINF_obs | hr*hr*ug/L | 343521.1782 |
| 42 | AUMC_%Extrap_obs | % | 42.1129 |
| 42 | AUMCINF_pred | hr*hr*ug/L | 339777.1730 |
| 42 | AUMC_%Extrap_pred | % | 41.4751 |
| 42 | MRTlast | hr | 10.9284 |
| 42 | MRTINF_obs | hr | 17.9857 |
| 42 | MRTINF_pred | hr | 17.8115 |
| 43 | Rsq |  | 0.7982 |
| 43 | Rsq_adjusted |  | 0.7758 |
| 43 | Corr_XY |  | -0.8934 |
| 43 | No_points_lambda_z |  | 11.0000 |
| 43 | Lambda_z | 1/hr | 0.0493 |
| 43 | Lambda_z_lower | hr | 2.0000 |
| 43 | Lambda_z_upper | hr | 96.0000 |
| 43 | HL_Lambda_z | hr | 14.0647 |
| 43 | Tlag | hr | 0.0000 |
| 43 | Tmax | hr | 2.0000 |
| 43 | Cmax | ug/L | 2130.0000 |
| 43 | Cmax_D | kg*ug/L/mg | 1002.3529 |
| 43 | Tlast | hr | 96.0000 |
| 43 | Clast | ug/L | 20.2000 |
| 43 | AUClast | hr*ug/L | 18303.3397 |
| 43 | AUCall | hr*ug/L | 18303.3397 |
| 43 | AUCINF_obs | hr*ug/L | 18713.2207 |
| 43 | AUCINF_D_obs | hr*kg*ug/L/mg | 8806.2215 |
| 43 | AUC_%Extrap_obs | % | 2.1903 |
| 43 | Vz_F_obs | L/kg | 2.3042 |
| 43 | Cl_F_obs | L/hr/kg | 0.1136 |
| 43 | AUCINF_pred | hr*ug/L | 18458.7157 |
| 43 | AUCINF_D_pred | hr*kg*ug/L/mg | 8686.4544 |
| 43 | AUC_%Extrap_pred | % | 0.8417 |
| 43 | Vz_F_pred | L/kg | 2.3360 |
| 43 | Cl_F_pred | L/hr/kg | 0.1151 |
| 43 | AUMClast | hr*hr*ug/L | 240394.2379 |
| 43 | AUMCINF_obs | hr*hr*ug/L | 288059.7690 |
| 43 | AUMC_%Extrap_obs | % | 16.5471 |
| 43 | AUMCINF_pred | hr*hr*ug/L | 258463.0866 |
| 43 | AUMC_%Extrap_pred | % | 6.9909 |
| 43 | MRTlast | hr | 13.1339 |
| 43 | MRTINF_obs | hr | 15.3934 |
| 43 | MRTINF_pred | hr | 14.0022 |
| 44 | Rsq |  | 0.9515 |
| 44 | Rsq_adjusted |  | 0.9273 |
| 44 | Corr_XY |  | -0.9755 |
| 44 | No_points_lambda_z |  | 4.0000 |
| 44 | Lambda_z | 1/hr | 0.0124 |
| 44 | Lambda_z_lower | hr | 36.0000 |
| 44 | Lambda_z_upper | hr | 96.0000 |
| 44 | HL_Lambda_z | hr | 56.0072 |
| 44 | Tlag | hr | 0.0000 |
| 44 | Tmax | hr | 2.0000 |
| 44 | Cmax | ug/L | 2000.0000 |
| 44 | Cmax_D | kg*ug/L/mg | 941.1765 |
| 44 | Tlast | hr | 96.0000 |
| 44 | Clast | ug/L | 15.9000 |
| 44 | AUClast | hr*ug/L | 17429.4353 |
| 44 | AUCall | hr*ug/L | 17429.4353 |
| 44 | AUCINF_obs | hr*ug/L | 18714.1772 |
| 44 | AUCINF_D_obs | hr*kg*ug/L/mg | 8806.6716 |
| 44 | AUC_%Extrap_obs | % | 6.8651 |
| 44 | Vz_F_obs | L/kg | 9.1750 |
| 44 | Cl_F_obs | L/hr/kg | 0.1136 |
| 44 | AUCINF_pred | hr*ug/L | 18715.3159 |
| 44 | AUCINF_D_pred | hr*kg*ug/L/mg | 8807.2075 |
| 44 | AUC_%Extrap_pred | % | 6.8707 |
| 44 | Vz_F_pred | L/kg | 9.1745 |
| 44 | Cl_F_pred | L/hr/kg | 0.1135 |
| 44 | AUMClast | hr*hr*ug/L | 206600.0357 |
| 44 | AUMCINF_obs | hr*hr*ug/L | 433744.1811 |
| 44 | AUMC_%Extrap_obs | % | 52.3682 |
| 44 | AUMCINF_pred | hr*hr*ug/L | 433945.5151 |
| 44 | AUMC_%Extrap_pred | % | 52.3903 |
| 44 | MRTlast | hr | 11.8535 |
| 44 | MRTINF_obs | hr | 23.1773 |
| 44 | MRTINF_pred | hr | 23.1867 |
| 45 | Rsq |  | 0.9465 |
| 45 | Rsq_adjusted |  | 0.9197 |
| 45 | Corr_XY |  | -0.9729 |
| 45 | No_points_lambda_z |  | 4.0000 |
| 45 | Lambda_z | 1/hr | 0.0113 |
| 45 | Lambda_z_lower | hr | 36.0000 |
| 45 | Lambda_z_upper | hr | 96.0000 |
| 45 | HL_Lambda_z | hr | 61.5663 |
| 45 | Tlag | hr | 0.0000 |
| 45 | Tmax | hr | 2.0000 |
| 45 | Cmax | ug/L | 2110.0000 |
| 45 | Cmax_D | kg*ug/L/mg | 992.9412 |
| 45 | Tlast | hr | 96.0000 |
| 45 | Clast | ug/L | 11.8000 |
| 45 | AUClast | hr*ug/L | 16655.1881 |
| 45 | AUCall | hr*ug/L | 16655.1881 |
| 45 | AUCINF_obs | hr*ug/L | 17703.2805 |
| 45 | AUCINF_D_obs | hr*kg*ug/L/mg | 8330.9555 |
| 45 | AUC_%Extrap_obs | % | 5.9203 |
| 45 | Vz_F_obs | L/kg | 10.6616 |
| 45 | Cl_F_obs | L/hr/kg | 0.1200 |
| 45 | AUCINF_pred | hr*ug/L | 17729.2944 |
| 45 | AUCINF_D_pred | hr*kg*ug/L/mg | 8343.1973 |
| 45 | AUC_%Extrap_pred | % | 6.0584 |
| 45 | Vz_F_pred | L/kg | 10.6460 |
| 45 | Cl_F_pred | L/hr/kg | 0.1199 |
| 45 | AUMClast | hr*hr*ug/L | 164085.1957 |
| 45 | AUMCINF_obs | hr*hr*ug/L | 357795.0911 |
| 45 | AUMC_%Extrap_obs | % | 54.1399 |
| 45 | AUMCINF_pred | hr*hr*ug/L | 362603.0166 |
| 45 | AUMC_%Extrap_pred | % | 54.7480 |
| 45 | MRTlast | hr | 9.8519 |
| 45 | MRTINF_obs | hr | 20.2107 |
| 45 | MRTINF_pred | hr | 20.4522 |
| 46 | Rsq |  | 0.9655 |
| 46 | Rsq_adjusted |  | 0.9483 |
| 46 | Corr_XY |  | -0.9826 |
| 46 | No_points_lambda_z |  | 4.0000 |
| 46 | Lambda_z | 1/hr | 0.0188 |
| 46 | Lambda_z_lower | hr | 36.0000 |
| 46 | Lambda_z_upper | hr | 96.0000 |
| 46 | HL_Lambda_z | hr | 36.9116 |
| 46 | Tlag | hr | 0.0000 |
| 46 | Tmax | hr | 2.0000 |
| 46 | Cmax | ug/L | 1810.0000 |
| 46 | Cmax_D | kg*ug/L/mg | 851.7647 |
| 46 | Tlast | hr | 96.0000 |
| 46 | Clast | ug/L | 7.6000 |
| 46 | AUClast | hr*ug/L | 12792.5823 |
| 46 | AUCall | hr*ug/L | 12792.5823 |
| 46 | AUCINF_obs | hr*ug/L | 13197.2984 |
| 46 | AUCINF_D_obs | hr*kg*ug/L/mg | 6210.4934 |
| 46 | AUC_%Extrap_obs | % | 3.0667 |
| 46 | Vz_F_obs | L/kg | 8.5745 |
| 46 | Cl_F_obs | L/hr/kg | 0.1610 |
| 46 | AUCINF_pred | hr*ug/L | 13219.6625 |
| 46 | AUCINF_D_pred | hr*kg*ug/L/mg | 6221.0177 |
| 46 | AUC_%Extrap_pred | % | 3.2306 |
| 46 | Vz_F_pred | L/kg | 8.5600 |
| 46 | Cl_F_pred | L/hr/kg | 0.1607 |
| 46 | AUMClast | hr*hr*ug/L | 129240.7927 |
| 46 | AUMCINF_obs | hr*hr*ug/L | 189645.5341 |
| 46 | AUMC_%Extrap_obs | % | 31.8514 |
| 46 | AUMCINF_pred | hr*hr*ug/L | 192983.4201 |
| 46 | AUMC_%Extrap_pred | % | 33.0301 |
| 46 | MRTlast | hr | 10.1028 |
| 46 | MRTINF_obs | hr | 14.3700 |
| 46 | MRTINF_pred | hr | 14.5982 |
| 47 | Rsq |  | 0.9999 |
| 47 | Rsq_adjusted |  | 0.9998 |
| 47 | Corr_XY |  | -0.9999 |
| 47 | No_points_lambda_z |  | 3.0000 |
| 47 | Lambda_z | 1/hr | 0.0119 |
| 47 | Lambda_z_lower | hr | 48.0000 |
| 47 | Lambda_z_upper | hr | 96.0000 |
| 47 | HL_Lambda_z | hr | 58.0457 |
| 47 | Tlag | hr | 0.0000 |
| 47 | Tmax | hr | 2.0000 |
| 47 | Cmax | ug/L | 2520.0000 |
| 47 | Cmax_D | kg*ug/L/mg | 1185.8824 |
| 47 | Tlast | hr | 96.0000 |
| 47 | Clast | ug/L | 11.5000 |
| 47 | AUClast | hr*ug/L | 15454.3869 |
| 47 | AUCall | hr*ug/L | 15454.3869 |
| 47 | AUCINF_obs | hr*ug/L | 16417.4219 |
| 47 | AUCINF_D_obs | hr*kg*ug/L/mg | 7725.8456 |
| 47 | AUC_%Extrap_obs | % | 5.8659 |
| 47 | Vz_F_obs | L/kg | 10.8392 |
| 47 | Cl_F_obs | L/hr/kg | 0.1294 |
| 47 | AUCINF_pred | hr*ug/L | 16419.1655 |
| 47 | AUCINF_D_pred | hr*kg*ug/L/mg | 7726.6661 |
| 47 | AUC_%Extrap_pred | % | 5.8759 |
| 47 | Vz_F_pred | L/kg | 10.8381 |
| 47 | Cl_F_pred | L/hr/kg | 0.1294 |
| 47 | AUMClast | hr*hr*ug/L | 159437.0952 |
| 47 | AUMCINF_obs | hr*hr*ug/L | 332535.1107 |
| 47 | AUMC_%Extrap_obs | % | 52.0541 |
| 47 | AUMCINF_pred | hr*hr*ug/L | 332848.5019 |
| 47 | AUMC_%Extrap_pred | % | 52.0992 |
| 47 | MRTlast | hr | 10.3166 |
| 47 | MRTINF_obs | hr | 20.2550 |
| 47 | MRTINF_pred | hr | 20.2719 |
| 48 | Rsq |  | 0.9178 |
| 48 | Rsq_adjusted |  | 0.8767 |
| 48 | Corr_XY |  | -0.9580 |
| 48 | No_points_lambda_z |  | 4.0000 |
| 48 | Lambda_z | 1/hr | 0.0198 |
| 48 | Lambda_z_lower | hr | 36.0000 |
| 48 | Lambda_z_upper | hr | 96.0000 |
| 48 | HL_Lambda_z | hr | 35.0311 |
| 48 | Tlag | hr | 0.0000 |
| 48 | Tmax | hr | 2.0000 |
| 48 | Cmax | ug/L | 1920.0000 |
| 48 | Cmax_D | kg*ug/L/mg | 903.5294 |
| 48 | Tlast | hr | 96.0000 |
| 48 | Clast | ug/L | 8.4000 |
| 48 | AUClast | hr*ug/L | 14371.7897 |
| 48 | AUCall | hr*ug/L | 14371.7897 |
| 48 | AUCINF_obs | hr*ug/L | 14796.3189 |
| 48 | AUCINF_D_obs | hr*kg*ug/L/mg | 6962.9736 |
| 48 | AUC_%Extrap_obs | % | 2.8692 |
| 48 | Vz_F_obs | L/kg | 7.2583 |
| 48 | Cl_F_obs | L/hr/kg | 0.1436 |
| 48 | AUCINF_pred | hr*ug/L | 14833.0074 |
| 48 | AUCINF_D_pred | hr*kg*ug/L/mg | 6980.2388 |
| 48 | AUC_%Extrap_pred | % | 3.1094 |
| 48 | Vz_F_pred | L/kg | 7.2403 |
| 48 | Cl_F_pred | L/hr/kg | 0.1433 |
| 48 | AUMClast | hr*hr*ug/L | 162631.2134 |
| 48 | AUMCINF_obs | hr*hr*ug/L | 224841.3730 |
| 48 | AUMC_%Extrap_obs | % | 27.6685 |
| 48 | AUMCINF_pred | hr*hr*ug/L | 230217.6853 |
| 48 | AUMC_%Extrap_pred | % | 29.3576 |
| 48 | MRTlast | hr | 11.3160 |
| 48 | MRTINF_obs | hr | 15.1958 |
| 48 | MRTINF_pred | hr | 15.5206 |

| **Animal** | **Constant** | **Value** |
| --- | --- | --- |
| 25 | Dose | 2.125 |
| 25 | Time of Last Dose | 0 |
| 26 | Dose | 2.125 |
| 26 | Time of Last Dose | 0 |
| 27 | Dose | 2.125 |
| 27 | Time of Last Dose | 0 |
| 28 | Dose | 2.125 |
| 28 | Time of Last Dose | 0 |
| 29 | Dose | 2.125 |
| 29 | Time of Last Dose | 0 |
| 30 | Dose | 2.125 |
| 30 | Time of Last Dose | 0 |
| 31 | Dose | 2.125 |
| 31 | Time of Last Dose | 0 |
| 32 | Dose | 2.125 |
| 32 | Time of Last Dose | 0 |
| 33 | Dose | 2.125 |
| 33 | Time of Last Dose | 0 |
| 34 | Dose | 2.125 |
| 34 | Time of Last Dose | 0 |
| 35 | Dose | 2.125 |
| 35 | Time of Last Dose | 0 |
| 36 | Dose | 2.125 |
| 36 | Time of Last Dose | 0 |
| 37 | Dose | 2.125 |
| 37 | Time of Last Dose | 0 |
| 38 | Dose | 2.125 |
| 38 | Time of Last Dose | 0 |
| 39 | Dose | 2.125 |
| 39 | Time of Last Dose | 0 |
| 40 | Dose | 2.125 |
| 40 | Time of Last Dose | 0 |
| 41 | Dose | 2.125 |
| 41 | Time of Last Dose | 0 |
| 42 | Dose | 2.125 |
| 42 | Time of Last Dose | 0 |
| 43 | Dose | 2.125 |
| 43 | Time of Last Dose | 0 |
| 44 | Dose | 2.125 |
| 44 | Time of Last Dose | 0 |
| 45 | Dose | 2.125 |
| 45 | Time of Last Dose | 0 |
| 46 | Dose | 2.125 |
| 46 | Time of Last Dose | 0 |
| 47 | Dose | 2.125 |
| 47 | Time of Last Dose | 0 |
| 48 | Dose | 2.125 |
| 48 | Time of Last Dose | 0 |

| **Animal** | **Time (hr)** | **lambda_z_Incl** | **Concentration (ug/L)** | **Predicted (ug/L)** | **Residual (ug/L)** | **AUC (hr*ug/L)** | **AUMC (hr*hr*ug/L)** | **Weight** |
| --- | --- | --- | --- | --- | --- | --- | --- | --- |
| 25 | 0.0000 |  | 0.0000 |  |  | 0.0000 | 0.0000 | 0.0000 |
| 25 | 0.1667 |  | 210.0000 |  |  | 17.5035 | 2.9178 | 0.0000 |
| 25 | 0.5000 |  | 465.0000 |  |  | 129.9923 | 47.4979 | 0.0000 |
| 25 | 1.0000 |  | 1550.0000 |  |  | 633.7423 | 493.1229 | 0.0000 |
| 25 | 2.0000 |  | 2220.0000 |  |  | 2518.7423 | 3488.1229 | 0.0000 |
| 25 | 4.0000 |  | 1390.0000 |  |  | 6128.7423 | 13488.1229 | 0.0000 |
| 25 | 6.0000 |  | 1020.0000 |  |  | 8538.7423 | 25168.1229 | 0.0000 |
| 25 | 8.0000 |  | 468.0000 |  |  | 10026.7423 | 35032.1229 | 0.0000 |
| 25 | 10.0000 |  | 398.0000 |  |  | 10892.7423 | 42756.1229 | 0.0000 |
| 25 | 12.0000 |  | 199.0000 |  |  | 11489.7423 | 49124.1229 | 0.0000 |
| 25 | 24.0000 |  | 75.2000 |  |  | 13134.9423 | 74280.9229 | 0.0000 |
| 25 | 36.0000 | * | 32.2000 | 31.1347 | 1.0653 | 13779.3423 | 92064.9229 | 1.0000 |
| 25 | 48.0000 | * | 26.6000 | 26.2795 | 0.3205 | 14132.1423 | 106680.9229 | 1.0000 |
| 25 | 72.0000 | * | 16.8000 | 18.7225 | -1.9225 | 14652.9423 | 136517.7229 | 1.0000 |
| 25 | 96.0000 | * | 14.2000 | 13.3386 | 0.8614 | 15024.9423 | 167391.3229 | 1.0000 |
| 26 | 0.0000 |  | 0.0000 |  |  | 0.0000 | 0.0000 | 0.0000 |
| 26 | 0.1667 |  | 237.0000 |  |  | 19.7540 | 3.2930 | 0.0000 |
| 26 | 0.5000 |  | 726.0000 |  |  | 180.2379 | 70.3709 | 0.0000 |
| 26 | 1.0000 |  | 1500.0000 |  |  | 736.7379 | 536.1209 | 0.0000 |
| 26 | 2.0000 |  | 2490.0000 |  |  | 2731.7379 | 3776.1209 | 0.0000 |
| 26 | 4.0000 |  | 1240.0000 |  |  | 6461.7379 | 13716.1209 | 0.0000 |
| 26 | 6.0000 |  | 654.0000 |  |  | 8355.7379 | 22600.1209 | 0.0000 |
| 26 | 8.0000 |  | 704.0000 |  |  | 9713.7379 | 32156.1209 | 0.0000 |
| 26 | 10.0000 |  | 296.0000 |  |  | 10713.7379 | 40748.1209 | 0.0000 |
| 26 | 12.0000 |  | 288.0000 |  |  | 11297.7379 | 47164.1209 | 0.0000 |
| 26 | 24.0000 |  | 73.6000 |  |  | 13467.3379 | 78498.5209 | 0.0000 |
| 26 | 36.0000 | * | 30.2000 | 31.6914 | -1.4914 | 14090.1379 | 95620.1209 | 1.0000 |
| 26 | 48.0000 | * | 28.4000 | 26.0171 | 2.3829 | 14441.7379 | 110322.5209 | 1.0000 |
| 26 | 72.0000 | * | 16.6000 | 17.5344 | -0.9344 | 14981.7379 | 141023.3209 | 1.0000 |
| 26 | 96.0000 | * | 12.0000 | 11.8174 | 0.1826 | 15324.9379 | 169189.7209 | 1.0000 |
| 27 | 0.0000 |  | 0.0000 |  |  | 0.0000 | 0.0000 | 0.0000 |
| 27 | 0.1667 |  | 126.0000 |  |  | 10.5021 | 1.7507 | 0.0000 |
| 27 | 0.5000 |  | 699.0000 |  |  | 147.9884 | 63.4952 | 0.0000 |
| 27 | 1.0000 |  | 1350.0000 |  |  | 660.2384 | 488.3702 | 0.0000 |
| 27 | 2.0000 |  | 2750.0000 |  |  | 2710.2384 | 3913.3702 | 0.0000 |
| 27 | 4.0000 |  | 1370.0000 |  |  | 6830.2384 | 14893.3702 | 0.0000 |
| 27 | 6.0000 |  | 696.0000 |  |  | 8896.2384 | 24549.3702 | 0.0000 |
| 27 | 8.0000 |  | 452.0000 |  |  | 10044.2384 | 32341.3702 | 0.0000 |
| 27 | 10.0000 |  | 330.0000 |  |  | 10826.2384 | 39257.3702 | 0.0000 |
| 27 | 12.0000 |  | 232.0000 |  |  | 11388.2384 | 45341.3702 | 0.0000 |
| 27 | 24.0000 |  | 106.4000 |  |  | 13418.6384 | 77366.9702 | 0.0000 |
| 27 | 36.0000 | * | 34.8000 | 32.5943 | 2.2057 | 14265.8384 | 100205.3702 | 1.0000 |
| 27 | 48.0000 | * | 26.4000 | 26.4344 | -0.0344 | 14633.0384 | 115325.3702 | 1.0000 |
| 27 | 72.0000 | * | 14.8000 | 17.3870 | -2.5870 | 15127.4384 | 143318.9702 | 1.0000 |
| 27 | 96.0000 | * | 12.6000 | 11.4361 | 1.1639 | 15456.2384 | 170621.3702 | 1.0000 |
| 28 | 0.0000 |  | 0.0000 |  |  | 0.0000 | 0.0000 | 0.0000 |
| 28 | 0.1667 |  | 160.0000 |  |  | 13.3360 | 2.2231 | 0.0000 |
| 28 | 0.5000 |  | 819.0000 |  |  | 176.4864 | 74.9112 | 0.0000 |
| 28 | 1.0000 |  | 1650.0000 |  |  | 793.7364 | 589.7862 | 0.0000 |
| 28 | 2.0000 |  | 2570.0000 |  |  | 2903.7364 | 3984.7862 | 0.0000 |
| 28 | 4.0000 |  | 1430.0000 |  |  | 6903.7364 | 14844.7862 | 0.0000 |
| 28 | 6.0000 |  | 1128.0000 |  |  | 9461.7364 | 27332.7862 | 0.0000 |
| 28 | 8.0000 |  | 700.0000 |  |  | 11289.7364 | 39700.7862 | 0.0000 |
| 28 | 10.0000 |  | 426.0000 |  |  | 12415.7364 | 49560.7862 | 0.0000 |
| 28 | 12.0000 |  | 263.0000 |  |  | 13104.7364 | 56976.7862 | 0.0000 |
| 28 | 24.0000 |  | 157.6000 |  |  | 15628.3364 | 98607.1862 | 0.0000 |
| 28 | 36.0000 |  | 47.6000 |  |  | 16859.5364 | 131583.1862 | 0.0000 |
| 28 | 48.0000 | * | 25.8000 | 25.8808 | -0.0808 | 17299.9364 | 149295.1862 | 1.0000 |
| 28 | 72.0000 | * | 18.2000 | 18.0865 | 0.1135 | 17827.9364 | 179880.7862 | 1.0000 |
| 28 | 96.0000 | * | 12.6000 | 12.6395 | -0.0395 | 18197.5364 | 210120.7862 | 1.0000 |
| 29 | 0.0000 |  | 0.0000 |  |  | 0.0000 | 0.0000 | 0.0000 |
| 29 | 0.1667 |  | 222.0000 |  |  | 18.5037 | 3.0846 | 0.0000 |
| 29 | 0.5000 |  | 837.0000 |  |  | 194.9861 | 78.9949 | 0.0000 |
| 29 | 1.0000 |  | 1430.0000 |  |  | 761.7361 | 541.1199 | 0.0000 |
| 29 | 2.0000 |  | 2210.0000 |  |  | 2581.7361 | 3466.1199 | 0.0000 |
| 29 | 4.0000 |  | 1830.0000 |  |  | 6621.7361 | 15206.1199 | 0.0000 |
| 29 | 6.0000 |  | 708.0000 |  |  | 9159.7361 | 26774.1199 | 0.0000 |
| 29 | 8.0000 |  | 828.0000 |  |  | 10695.7361 | 37646.1199 | 0.0000 |
| 29 | 10.0000 |  | 438.0000 |  |  | 11961.7361 | 48650.1199 | 0.0000 |
| 29 | 12.0000 |  | 312.0000 |  |  | 12711.7361 | 56774.1199 | 0.0000 |
| 29 | 24.0000 |  | 125.6000 |  |  | 15337.3361 | 97324.5199 | 0.0000 |
| 29 | 36.0000 | * | 43.8000 | 43.5360 | 0.2640 | 16353.7361 | 124871.7199 | 1.0000 |
| 29 | 48.0000 | * | 32.0000 | 33.5585 | -1.5585 | 16808.5361 | 143548.5199 | 1.0000 |
| 29 | 72.0000 | * | 21.6000 | 19.9394 | 1.6606 | 17451.7361 | 180642.9199 | 1.0000 |
| 29 | 96.0000 | * | 11.4000 | 11.8473 | -0.4473 | 17847.7361 | 212438.1199 | 1.0000 |
| 30 | 0.0000 |  | 0.0000 |  |  | 0.0000 | 0.0000 | 0.0000 |
| 30 | 0.1667 |  | 139.0000 |  |  | 11.5857 | 1.9313 | 0.0000 |
| 30 | 0.5000 |  | 645.0000 |  |  | 142.2393 | 59.5375 | 0.0000 |
| 30 | 1.0000 |  | 1850.0000 |  |  | 765.9893 | 602.6625 | 0.0000 |
| 30 | 2.0000 |  | 2100.0000 |  |  | 2740.9893 | 3627.6625 | 0.0000 |
| 30 | 4.0000 |  | 1760.0000 |  |  | 6600.9893 | 14867.6625 | 0.0000 |
| 30 | 6.0000 |  | 1038.0000 |  |  | 9398.9893 | 28135.6625 | 0.0000 |
| 30 | 8.0000 |  | 780.0000 |  |  | 11216.9893 | 40603.6625 | 0.0000 |
| 30 | 10.0000 | * | 366.0000 | 187.3420 | 178.6580 | 12362.9893 | 50503.6625 | 1.0000 |
| 30 | 12.0000 | * | 236.0000 | 174.0433 | 61.9567 | 12964.9893 | 56995.6625 | 1.0000 |
| 30 | 24.0000 | * | 94.0000 | 111.8896 | -17.8896 | 14944.9893 | 87523.6625 | 1.0000 |
| 30 | 36.0000 | * | 31.6000 | 71.9320 | -40.3320 | 15698.5893 | 107885.2625 | 1.0000 |
| 30 | 48.0000 | * | 34.2000 | 46.2439 | -12.0439 | 16093.3893 | 124560.4625 | 1.0000 |
| 30 | 72.0000 | * | 14.4000 | 19.1126 | -4.7126 | 16676.5893 | 156701.2625 | 1.0000 |
| 30 | 96.0000 | * | 14.5000 | 7.8992 | 6.6008 | 17023.3893 | 185846.8625 | 1.0000 |
| 31 | 0.0000 |  | 0.0000 |  |  | 0.0000 | 0.0000 | 0.0000 |
| 31 | 0.1667 |  | 199.0000 |  |  | 16.5867 | 2.7650 | 0.0000 |
| 31 | 0.5000 |  | 804.0000 |  |  | 183.7366 | 75.2866 | 0.0000 |
| 31 | 1.0000 |  | 1540.0000 |  |  | 769.7366 | 560.7866 | 0.0000 |
| 31 | 2.0000 |  | 2330.0000 |  |  | 2704.7366 | 3660.7866 | 0.0000 |
| 31 | 4.0000 | * | 1080.0000 | 467.0017 | 612.9983 | 6114.7366 | 12640.7866 | 1.0000 |
| 31 | 6.0000 | * | 666.0000 | 425.6467 | 240.3533 | 7860.7366 | 20956.7866 | 1.0000 |
| 31 | 8.0000 | * | 772.0000 | 387.9539 | 384.0461 | 9298.7366 | 31128.7866 | 1.0000 |
| 31 | 10.0000 | * | 384.0000 | 353.5989 | 30.4011 | 10454.7366 | 41144.7866 | 1.0000 |
| 31 | 12.0000 | * | 210.0000 | 322.2862 | -112.2862 | 11048.7366 | 47504.7866 | 1.0000 |
| 31 | 24.0000 | * | 98.4000 | 184.7680 | -86.3680 | 12899.1366 | 76794.3866 | 1.0000 |
| 31 | 36.0000 | * | 39.4000 | 105.9282 | -66.5282 | 13725.9366 | 99474.3866 | 1.0000 |
| 31 | 48.0000 | * | 29.4000 | 60.7291 | -31.3291 | 14138.7366 | 116451.9866 | 1.0000 |
| 31 | 72.0000 | * | 13.4000 | 19.9603 | -6.5603 | 14652.3366 | 144963.9866 | 1.0000 |
| 31 | 96.0000 | * | 20.0000 | 6.5605 | 13.4395 | 15053.1366 | 179581.5866 | 1.0000 |
| 32 | 0.0000 |  | 0.0000 |  |  | 0.0000 | 0.0000 | 0.0000 |
| 32 | 0.1667 |  | 208.0000 |  |  | 17.3368 | 2.8900 | 0.0000 |
| 32 | 0.5000 |  | 927.0000 |  |  | 206.4846 | 85.9107 | 0.0000 |
| 32 | 1.0000 |  | 1760.0000 |  |  | 878.2346 | 641.7857 | 0.0000 |
| 32 | 2.0000 |  | 1610.0000 |  |  | 2563.2346 | 3131.7857 | 0.0000 |
| 32 | 4.0000 |  | 980.0000 |  |  | 5153.2346 | 10271.7857 | 0.0000 |
| 32 | 6.0000 |  | 528.0000 |  |  | 6661.2346 | 17359.7857 | 0.0000 |
| 32 | 8.0000 |  | 596.0000 |  |  | 7785.2346 | 25295.7857 | 0.0000 |
| 32 | 10.0000 |  | 334.0000 |  |  | 8715.2346 | 33403.7857 | 0.0000 |
| 32 | 12.0000 |  | 224.0000 |  |  | 9273.2346 | 39431.7857 | 0.0000 |
| 32 | 24.0000 |  | 76.8000 |  |  | 11078.0346 | 66618.9857 | 0.0000 |
| 32 | 36.0000 | * | 35.4000 | 32.5680 | 2.8320 | 11751.2346 | 85324.5857 | 1.0000 |
| 32 | 48.0000 | * | 27.4000 | 27.4192 | -0.0192 | 12128.0346 | 100862.1857 | 1.0000 |
| 32 | 72.0000 | * | 15.8000 | 19.4349 | -3.6349 | 12646.4346 | 130295.7857 | 1.0000 |
| 32 | 96.0000 | * | 15.6000 | 13.7756 | 1.8244 | 13023.2346 | 161918.1857 | 1.0000 |
| 33 | 0.0000 |  | 0.0000 |  |  | 0.0000 | 0.0000 | 0.0000 |
| 33 | 0.1667 |  | 212.0000 |  |  | 17.6702 | 2.9456 | 0.0000 |
| 33 | 0.5000 |  | 717.0000 |  |  | 172.4881 | 68.5791 | 0.0000 |
| 33 | 1.0000 |  | 1220.0000 |  |  | 656.7381 | 463.2041 | 0.0000 |
| 33 | 2.0000 |  | 2380.0000 |  |  | 2456.7381 | 3453.2041 | 0.0000 |
| 33 | 4.0000 |  | 1340.0000 |  |  | 6176.7381 | 13573.2041 | 0.0000 |
| 33 | 6.0000 |  | 558.0000 |  |  | 8074.7381 | 22281.2041 | 0.0000 |
| 33 | 8.0000 |  | 536.0000 |  |  | 9168.7381 | 29917.2041 | 0.0000 |
| 33 | 10.0000 |  | 374.0000 |  |  | 10078.7381 | 37945.2041 | 0.0000 |
| 33 | 12.0000 |  | 203.0000 |  |  | 10655.7381 | 44121.2041 | 0.0000 |
| 33 | 24.0000 |  | 86.0000 |  |  | 12389.7381 | 71121.2041 | 0.0000 |
| 33 | 36.0000 |  | 31.4000 |  |  | 13094.1381 | 90287.6041 | 0.0000 |
| 33 | 48.0000 | * | 24.4000 | 24.2913 | 0.1087 | 13428.9381 | 104097.2041 | 1.0000 |
| 33 | 72.0000 | * | 16.6000 | 16.7489 | -0.1489 | 13920.9381 | 132494.0041 | 1.0000 |
| 33 | 96.0000 | * | 11.6000 | 11.5483 | 0.0517 | 14259.3381 | 160199.6041 | 1.0000 |
| 34 | 0.0000 |  | 0.0000 |  |  | 0.0000 | 0.0000 | 0.0000 |
| 34 | 0.1667 |  | 238.0000 |  |  | 19.8373 | 3.3069 | 0.0000 |
| 34 | 0.5000 |  | 807.0000 |  |  | 193.9866 | 77.1619 | 0.0000 |
| 34 | 1.0000 |  | 1740.0000 |  |  | 830.7366 | 613.0369 | 0.0000 |
| 34 | 2.0000 |  | 2350.0000 |  |  | 2875.7366 | 3833.0369 | 0.0000 |
| 34 | 4.0000 |  | 1250.0000 |  |  | 6475.7366 | 13533.0369 | 0.0000 |
| 34 | 6.0000 |  | 618.0000 |  |  | 8343.7366 | 22241.0369 | 0.0000 |
| 34 | 8.0000 |  | 848.0000 |  |  | 9809.7366 | 32733.0369 | 0.0000 |
| 34 | 10.0000 |  | 324.0000 |  |  | 10981.7366 | 42757.0369 | 0.0000 |
| 34 | 12.0000 | * | 185.0000 | 140.9613 | 44.0387 | 11490.7366 | 48217.0369 | 1.0000 |
| 34 | 24.0000 | * | 112.4000 | 93.0819 | 19.3181 | 13275.1366 | 77722.6369 | 1.0000 |
| 34 | 36.0000 | * | 37.6000 | 61.4653 | -23.8653 | 14175.1366 | 102029.8369 | 1.0000 |
| 34 | 48.0000 | * | 29.8000 | 40.5878 | -10.7878 | 14579.5366 | 118733.8369 | 1.0000 |
| 34 | 72.0000 | * | 24.6000 | 17.6981 | 6.9019 | 15232.3366 | 157153.0369 | 1.0000 |
| 34 | 96.0000 | * | 7.8000 | 7.7171 | 0.0829 | 15621.1366 | 187393.0369 | 1.0000 |
| 35 | 0.0000 |  | 0.0000 |  |  | 0.0000 | 0.0000 | 0.0000 |
| 35 | 0.1667 |  | 161.0000 |  |  | 13.4194 | 2.2370 | 0.0000 |
| 35 | 0.5000 |  | 639.0000 |  |  | 146.7394 | 59.9544 | 0.0000 |
| 35 | 1.0000 |  | 1170.0000 |  |  | 598.9894 | 432.3294 | 0.0000 |
| 35 | 2.0000 |  | 2180.0000 |  |  | 2273.9894 | 3197.3294 | 0.0000 |
| 35 | 4.0000 |  | 1100.0000 |  |  | 5553.9894 | 11957.3294 | 0.0000 |
| 35 | 6.0000 |  | 498.0000 |  |  | 7151.9894 | 19345.3294 | 0.0000 |
| 35 | 8.0000 |  | 448.0000 |  |  | 8097.9894 | 25917.3294 | 0.0000 |
| 35 | 10.0000 |  | 310.0000 |  |  | 8855.9894 | 32601.3294 | 0.0000 |
| 35 | 12.0000 |  | 120.0000 |  |  | 9285.9894 | 37141.3294 | 0.0000 |
| 35 | 24.0000 |  | 88.8000 |  |  | 10538.7894 | 58568.5294 | 0.0000 |
| 35 | 36.0000 | * | 29.6000 | 30.0323 | -0.4323 | 11249.1894 | 77749.3294 | 1.0000 |
| 35 | 48.0000 | * | 25.6000 | 24.2793 | 1.3207 | 11580.3894 | 91515.7294 | 1.0000 |
| 35 | 72.0000 | * | 14.8000 | 15.8683 | -1.0683 | 12065.1894 | 119048.5294 | 1.0000 |
| 35 | 96.0000 | * | 10.7000 | 10.3711 | 0.3289 | 12371.1894 | 144162.1294 | 1.0000 |
| 36 | 0.0000 |  | 0.0000 |  |  | 0.0000 | 0.0000 | 0.0000 |
| 36 | 0.1667 |  | 195.0000 |  |  | 16.2533 | 2.7094 | 0.0000 |
| 36 | 0.5000 |  | 642.0000 |  |  | 155.7393 | 61.6213 | 0.0000 |
| 36 | 1.0000 |  | 1470.0000 |  |  | 683.7393 | 509.3713 | 0.0000 |
| 36 | 2.0000 |  | 2360.0000 |  |  | 2598.7393 | 3604.3713 | 0.0000 |
| 36 | 4.0000 |  | 1430.0000 |  |  | 6388.7393 | 14044.3713 | 0.0000 |
| 36 | 6.0000 |  | 684.0000 |  |  | 8502.7393 | 23868.3713 | 0.0000 |
| 36 | 8.0000 |  | 848.0000 |  |  | 10034.7393 | 34756.3713 | 0.0000 |
| 36 | 10.0000 |  | 366.0000 |  |  | 11248.7393 | 45200.3713 | 0.0000 |
| 36 | 12.0000 |  | 191.0000 |  |  | 11805.7393 | 51152.3713 | 0.0000 |
| 36 | 24.0000 |  | 98.8000 |  |  | 13544.5393 | 79131.5713 | 0.0000 |
| 36 | 36.0000 | * | 29.0000 | 29.5324 | -0.5324 | 14311.3393 | 99622.7713 | 1.0000 |
| 36 | 48.0000 | * | 23.0000 | 23.7110 | -0.7110 | 14623.3393 | 112510.7713 | 1.0000 |
| 36 | 72.0000 | * | 17.0000 | 15.2846 | 1.7154 | 15103.3393 | 140446.7713 | 1.0000 |
| 36 | 96.0000 | * | 9.3000 | 9.8527 | -0.5527 | 15418.9393 | 165848.3713 | 1.0000 |
| 37 | 0.0000 |  | 0.0000 |  |  | 0.0000 | 0.0000 | 0.0000 |
| 37 | 0.1667 |  | 169.0000 |  |  | 14.0862 | 2.3482 | 0.0000 |
| 37 | 0.5000 |  | 714.0000 |  |  | 161.2381 | 66.5371 | 0.0000 |
| 37 | 1.0000 |  | 1540.0000 |  |  | 724.7381 | 540.7871 | 0.0000 |
| 37 | 2.0000 | * | 2010.0000 | 649.3150 | 1360.6850 | 2499.7381 | 3320.7871 | 1.0000 |
| 37 | 4.0000 | * | 1840.0000 | 587.6833 | 1252.3167 | 6349.7381 | 14700.7871 | 1.0000 |
| 37 | 6.0000 | * | 786.0000 | 531.9016 | 254.0984 | 8975.7381 | 26776.7871 | 1.0000 |
| 37 | 8.0000 | * | 624.0000 | 481.4146 | 142.5854 | 10385.7381 | 36484.7871 | 1.0000 |
| 37 | 10.0000 | * | 242.0000 | 435.7197 | -193.7197 | 11251.7381 | 43896.7871 | 1.0000 |
| 37 | 12.0000 | * | 313.0000 | 394.3621 | -81.3621 | 11806.7381 | 50072.7871 | 1.0000 |
| 37 | 24.0000 | * | 73.2000 | 216.7818 | -143.5818 | 14123.9381 | 83149.5871 | 1.0000 |
| 37 | 36.0000 | * | 32.8000 | 119.1655 | -86.3655 | 14759.9381 | 100775.1871 | 1.0000 |
| 37 | 48.0000 | * | 19.2000 | 65.5056 | -46.3056 | 15071.9381 | 113389.5871 | 1.0000 |
| 37 | 72.0000 | * | 37.4000 | 19.7940 | 17.6060 | 15751.1381 | 156762.3871 | 1.0000 |
| 37 | 96.0000 | * | 14.2000 | 5.9812 | 8.2188 | 16370.3381 | 205434.3871 | 1.0000 |
| 38 | 0.0000 |  | 0.0000 |  |  | 0.0000 | 0.0000 | 0.0000 |
| 38 | 0.1667 |  | 159.0000 |  |  | 13.2527 | 2.2092 | 0.0000 |
| 38 | 0.5000 |  | 864.0000 |  |  | 183.7356 | 78.6191 | 0.0000 |
| 38 | 1.0000 |  | 1840.0000 |  |  | 859.7356 | 646.6191 | 0.0000 |
| 38 | 2.0000 |  | 2570.0000 |  |  | 3064.7356 | 4136.6191 | 0.0000 |
| 38 | 4.0000 |  | 1750.0000 |  |  | 7384.7356 | 16276.6191 | 0.0000 |
| 38 | 6.0000 |  | 654.0000 |  |  | 9788.7356 | 27200.6191 | 0.0000 |
| 38 | 8.0000 |  | 752.0000 |  |  | 11194.7356 | 37140.6191 | 0.0000 |
| 38 | 10.0000 |  | 340.0000 |  |  | 12286.7356 | 46556.6191 | 0.0000 |
| 38 | 12.0000 |  | 195.0000 |  |  | 12821.7356 | 52296.6191 | 0.0000 |
| 38 | 24.0000 |  | 80.4000 |  |  | 14474.1356 | 77914.2191 | 0.0000 |
| 38 | 36.0000 |  | 34.6000 |  |  | 15164.1356 | 96965.4191 | 0.0000 |
| 38 | 48.0000 | * | 19.0000 | 19.8212 | -0.8212 | 15485.7356 | 109911.0191 | 1.0000 |
| 38 | 72.0000 | * | 17.0000 | 15.6205 | 1.3795 | 15917.7356 | 135543.0191 | 1.0000 |
| 38 | 96.0000 | * | 11.8000 | 12.3100 | -0.5100 | 16263.3356 | 163824.6191 | 1.0000 |
| 39 | 0.0000 |  | 0.0000 |  |  | 0.0000 | 0.0000 | 0.0000 |
| 39 | 0.1667 |  | 217.0000 |  |  | 18.0870 | 3.0151 | 0.0000 |
| 39 | 0.5000 |  | 636.0000 |  |  | 160.2394 | 62.0382 | 0.0000 |
| 39 | 1.0000 |  | 1340.0000 |  |  | 654.2394 | 476.5382 | 0.0000 |
| 39 | 2.0000 | * | 2200.0000 | 526.7949 | 1673.2051 | 2424.2394 | 3346.5382 | 1.0000 |
| 39 | 4.0000 | * | 1390.0000 | 473.7907 | 916.2093 | 6014.2394 | 13306.5382 | 1.0000 |
| 39 | 6.0000 | * | 504.0000 | 426.1197 | 77.8803 | 7908.2394 | 21890.5382 | 1.0000 |
| 39 | 8.0000 | * | 632.0000 | 383.2451 | 248.7549 | 9044.2394 | 29970.5382 | 1.0000 |
| 39 | 10.0000 | * | 276.0000 | 344.6844 | -68.6844 | 9952.2394 | 37786.5382 | 1.0000 |
| 39 | 12.0000 | * | 166.0000 | 310.0035 | -144.0035 | 10394.2394 | 42538.5382 | 1.0000 |
| 39 | 24.0000 | * | 47.2000 | 164.0728 | -116.8728 | 11673.4394 | 61287.3382 | 1.0000 |
| 39 | 36.0000 | * | 21.0000 | 86.8374 | -65.8374 | 12082.6394 | 72620.1382 | 1.0000 |
| 39 | 48.0000 | * | 15.6000 | 45.9596 | -30.3596 | 12302.2394 | 81648.9382 | 1.0000 |
| 39 | 72.0000 | * | 16.4000 | 12.8741 | 3.5259 | 12686.2394 | 104804.1382 | 1.0000 |
| 39 | 96.0000 | * | 11.7000 | 3.6063 | 8.0937 | 13023.4394 | 132452.1382 | 1.0000 |
| 40 | 0.0000 |  | 0.0000 |  |  | 0.0000 | 0.0000 | 0.0000 |
| 40 | 0.1667 |  | 205.0000 |  |  | 17.0868 | 2.8484 | 0.0000 |
| 40 | 0.5000 |  | 699.0000 |  |  | 167.7384 | 66.7876 | 0.0000 |
| 40 | 1.0000 |  | 1190.0000 |  |  | 639.9884 | 451.6626 | 0.0000 |
| 40 | 2.0000 |  | 2470.0000 |  |  | 2469.9884 | 3516.6626 | 0.0000 |
| 40 | 4.0000 |  | 1510.0000 |  |  | 6449.9884 | 14496.6626 | 0.0000 |
| 40 | 6.0000 |  | 540.0000 |  |  | 8499.9884 | 23776.6626 | 0.0000 |
| 40 | 8.0000 |  | 452.0000 |  |  | 9491.9884 | 30632.6626 | 0.0000 |
| 40 | 10.0000 |  | 652.0000 |  |  | 10595.9884 | 40768.6626 | 0.0000 |
| 40 | 12.0000 |  | 346.0000 |  |  | 11593.9884 | 51440.6626 | 0.0000 |
| 40 | 24.0000 |  | 62.0000 |  |  | 14041.9884 | 85280.6626 | 0.0000 |
| 40 | 36.0000 | * | 27.4000 | 25.1210 | 2.2790 | 14578.3884 | 100127.0626 | 1.0000 |
| 40 | 48.0000 | * | 19.2000 | 21.8423 | -2.6423 | 14857.9884 | 111575.0626 | 1.0000 |
| 40 | 72.0000 | * | 17.2000 | 16.5127 | 0.6873 | 15294.7884 | 137495.0626 | 1.0000 |
| 40 | 96.0000 | * | 12.5000 | 12.4835 | 0.0165 | 15651.1884 | 166755.8626 | 1.0000 |
| 41 | 0.0000 |  | 0.0000 |  |  | 0.0000 | 0.0000 | 0.0000 |
| 41 | 0.1667 |  | 169.0000 |  |  | 14.0862 | 2.3482 | 0.0000 |
| 41 | 0.5000 |  | 642.0000 |  |  | 149.2393 | 60.5377 | 0.0000 |
| 41 | 1.0000 |  | 1340.0000 |  |  | 644.7393 | 475.7877 | 0.0000 |
| 41 | 2.0000 |  | 2730.0000 |  |  | 2679.7393 | 3875.7877 | 0.0000 |
| 41 | 4.0000 |  | 1730.0000 |  |  | 7139.7393 | 16255.7877 | 0.0000 |
| 41 | 6.0000 |  | 684.0000 |  |  | 9553.7393 | 27279.7877 | 0.0000 |
| 41 | 8.0000 |  | 484.0000 |  |  | 10721.7393 | 35255.7877 | 0.0000 |
| 41 | 10.0000 |  | 212.0000 |  |  | 11417.7393 | 41247.7877 | 0.0000 |
| 41 | 12.0000 |  | 329.0000 |  |  | 11958.7393 | 47315.7877 | 0.0000 |
| 41 | 24.0000 |  | 77.2000 |  |  | 14395.9393 | 82120.5877 | 0.0000 |
| 41 | 36.0000 | * | 28.8000 | 26.4100 | 2.3900 | 15031.9393 | 99458.1877 | 1.0000 |
| 41 | 48.0000 | * | 18.8000 | 21.6116 | -2.8116 | 15317.5393 | 111093.3877 | 1.0000 |
| 41 | 72.0000 | * | 15.4000 | 14.4719 | 0.9281 | 15727.9393 | 135227.7877 | 1.0000 |
| 41 | 96.0000 | * | 9.6000 | 9.6909 | -0.0909 | 16027.9393 | 159592.5877 | 1.0000 |
| 42 | 0.0000 |  | 0.0000 |  |  | 0.0000 | 0.0000 | 0.0000 |
| 42 | 0.1667 |  | 149.0000 |  |  | 12.4192 | 2.0703 | 0.0000 |
| 42 | 0.5000 |  | 540.0000 |  |  | 127.2410 | 51.2051 | 0.0000 |
| 42 | 1.0000 |  | 1520.0000 |  |  | 642.2410 | 498.7051 | 0.0000 |
| 42 | 2.0000 |  | 2750.0000 |  |  | 2777.2410 | 4008.7051 | 0.0000 |
| 42 | 4.0000 |  | 1430.0000 |  |  | 6957.2410 | 15228.7051 | 0.0000 |
| 42 | 6.0000 |  | 1128.0000 |  |  | 9515.2410 | 27716.7051 | 0.0000 |
| 42 | 8.0000 |  | 812.0000 |  |  | 11455.2410 | 40980.7051 | 0.0000 |
| 42 | 10.0000 |  | 372.0000 |  |  | 12639.2410 | 51196.7051 | 0.0000 |
| 42 | 12.0000 |  | 386.0000 |  |  | 13397.2410 | 59548.7051 | 0.0000 |
| 42 | 24.0000 |  | 75.2000 |  |  | 16164.4410 | 98169.5051 | 0.0000 |
| 42 | 36.0000 | * | 35.2000 | 35.0279 | 0.1721 | 16826.8410 | 116601.5051 | 1.0000 |
| 42 | 48.0000 | * | 29.6000 | 29.0467 | 0.5533 | 17215.6410 | 132729.5051 | 1.0000 |
| 42 | 72.0000 | * | 19.0000 | 19.9740 | -0.9740 | 17798.8410 | 166195.1051 | 1.0000 |
| 42 | 96.0000 | * | 14.1000 | 13.7351 | 0.3649 | 18196.0410 | 198854.3051 | 1.0000 |
| 43 | 0.0000 |  | 0.0000 |  |  | 0.0000 | 0.0000 | 0.0000 |
| 43 | 0.1667 |  | 163.0000 |  |  | 13.5861 | 2.2648 | 0.0000 |
| 43 | 0.5000 |  | 618.0000 |  |  | 143.7397 | 58.2879 | 0.0000 |
| 43 | 1.0000 |  | 1650.0000 |  |  | 710.7397 | 548.0379 | 0.0000 |
| 43 | 2.0000 | * | 2130.0000 | 786.9993 | 1343.0007 | 2600.7397 | 3503.0379 | 1.0000 |
| 43 | 4.0000 | * | 1460.0000 | 713.1289 | 746.8711 | 6190.7397 | 13603.0379 | 1.0000 |
| 43 | 6.0000 | * | 1050.0000 | 646.1922 | 403.8078 | 8700.7397 | 25743.0379 | 1.0000 |
| 43 | 8.0000 | * | 432.0000 | 585.5384 | -153.5384 | 10182.7397 | 35499.0379 | 1.0000 |
| 43 | 10.0000 | * | 570.0000 | 530.5778 | 39.4222 | 11184.7397 | 44655.0379 | 1.0000 |
| 43 | 12.0000 | * | 461.0000 | 480.7760 | -19.7760 | 12215.7397 | 55887.0379 | 1.0000 |
| 43 | 24.0000 | * | 104.4000 | 266.1367 | -161.7367 | 15608.1397 | 104112.6379 | 1.0000 |
| 43 | 36.0000 | * | 48.4000 | 147.3218 | -98.9218 | 16524.9397 | 129600.6379 | 1.0000 |
| 43 | 48.0000 | * | 29.2000 | 81.5510 | -52.3510 | 16990.5397 | 148464.6379 | 1.0000 |
| 43 | 72.0000 | * | 30.0000 | 24.9892 | 5.0108 | 17700.9397 | 191203.8379 | 1.0000 |
| 43 | 96.0000 | * | 20.2000 | 7.6573 | 12.5427 | 18303.3397 | 240394.2379 | 1.0000 |
| 44 | 0.0000 |  | 0.0000 |  |  | 0.0000 | 0.0000 | 0.0000 |
| 44 | 0.1667 |  | 172.0000 |  |  | 14.3362 | 2.3898 | 0.0000 |
| 44 | 0.5000 |  | 885.0000 |  |  | 190.4853 | 80.9107 | 0.0000 |
| 44 | 1.0000 |  | 1590.0000 |  |  | 809.2353 | 589.0357 | 0.0000 |
| 44 | 2.0000 |  | 2000.0000 |  |  | 2604.2353 | 3384.0357 | 0.0000 |
| 44 | 4.0000 |  | 1460.0000 |  |  | 6064.2353 | 13224.0357 | 0.0000 |
| 44 | 6.0000 |  | 1056.0000 |  |  | 8580.2353 | 25400.0357 | 0.0000 |
| 44 | 8.0000 |  | 672.0000 |  |  | 10308.2353 | 37112.0357 | 0.0000 |
| 44 | 10.0000 |  | 492.0000 |  |  | 11472.2353 | 47408.0357 | 0.0000 |
| 44 | 12.0000 |  | 388.0000 |  |  | 12352.2353 | 56984.0357 | 0.0000 |
| 44 | 24.0000 |  | 94.0000 |  |  | 15244.2353 | 98456.0357 | 0.0000 |
| 44 | 36.0000 | * | 35.8000 | 33.4405 | 2.3595 | 16023.0353 | 119724.8357 | 1.0000 |
| 44 | 48.0000 | * | 26.0000 | 28.8253 | -2.8253 | 16393.8353 | 134945.6357 | 1.0000 |
| 44 | 72.0000 | * | 22.2000 | 21.4180 | 0.7820 | 16972.2353 | 169102.4357 | 1.0000 |
| 44 | 96.0000 | * | 15.9000 | 15.9141 | -0.0141 | 17429.4353 | 206600.0357 | 1.0000 |
| 45 | 0.0000 |  | 0.0000 |  |  | 0.0000 | 0.0000 | 0.0000 |
| 45 | 0.1667 |  | 195.0000 |  |  | 16.2533 | 2.7094 | 0.0000 |
| 45 | 0.5000 |  | 717.0000 |  |  | 168.2381 | 67.8707 | 0.0000 |
| 45 | 1.0000 |  | 1790.0000 |  |  | 794.9881 | 604.9957 | 0.0000 |
| 45 | 2.0000 |  | 2110.0000 |  |  | 2744.9881 | 3609.9957 | 0.0000 |
| 45 | 4.0000 |  | 1830.0000 |  |  | 6684.9881 | 15149.9957 | 0.0000 |
| 45 | 6.0000 |  | 1170.0000 |  |  | 9684.9881 | 29489.9957 | 0.0000 |
| 45 | 8.0000 |  | 592.0000 |  |  | 11446.9881 | 41245.9957 | 0.0000 |
| 45 | 10.0000 |  | 368.0000 |  |  | 12406.9881 | 49661.9957 | 0.0000 |
| 45 | 12.0000 |  | 285.0000 |  |  | 13059.9881 | 56761.9957 | 0.0000 |
| 45 | 24.0000 |  | 58.0000 |  |  | 15117.9881 | 85633.9957 | 0.0000 |
| 45 | 36.0000 | * | 24.8000 | 23.7630 | 1.0370 | 15614.7881 | 99342.7957 | 1.0000 |
| 45 | 48.0000 | * | 19.0000 | 20.7600 | -1.7600 | 15877.5881 | 110171.5957 | 1.0000 |
| 45 | 72.0000 | * | 17.0000 | 15.8445 | 1.1555 | 16309.5881 | 135803.5957 | 1.0000 |
| 45 | 96.0000 | * | 11.8000 | 12.0929 | -0.2929 | 16655.1881 | 164085.1957 | 1.0000 |
| 46 | 0.0000 |  | 0.0000 |  |  | 0.0000 | 0.0000 | 0.0000 |
| 46 | 0.1667 |  | 186.0000 |  |  | 15.5031 | 2.5844 | 0.0000 |
| 46 | 0.5000 |  | 1062.0000 |  |  | 223.4823 | 96.2427 | 0.0000 |
| 46 | 1.0000 |  | 1260.0000 |  |  | 803.9823 | 543.9927 | 0.0000 |
| 46 | 2.0000 |  | 1810.0000 |  |  | 2338.9823 | 2983.9927 | 0.0000 |
| 46 | 4.0000 |  | 1560.0000 |  |  | 5708.9823 | 12843.9927 | 0.0000 |
| 46 | 6.0000 |  | 582.0000 |  |  | 7850.9823 | 22575.9927 | 0.0000 |
| 46 | 8.0000 |  | 400.0000 |  |  | 8832.9823 | 29267.9927 | 0.0000 |
| 46 | 10.0000 |  | 288.0000 |  |  | 9520.9823 | 35347.9927 | 0.0000 |
| 46 | 12.0000 |  | 178.0000 |  |  | 9986.9823 | 40363.9927 | 0.0000 |
| 46 | 24.0000 |  | 56.4000 |  |  | 11393.3823 | 61301.5927 | 0.0000 |
| 46 | 36.0000 | * | 25.4000 | 24.7457 | 0.6543 | 11884.1823 | 74909.5927 | 1.0000 |
| 46 | 48.0000 | * | 18.0000 | 19.7530 | -1.7530 | 12144.5823 | 85579.9927 | 1.0000 |
| 46 | 72.0000 | * | 14.2000 | 12.5864 | 1.6136 | 12530.9823 | 108216.7927 | 1.0000 |
| 46 | 96.0000 | * | 7.6000 | 8.0200 | -0.4200 | 12792.5823 | 129240.7927 | 1.0000 |
| 47 | 0.0000 |  | 0.0000 |  |  | 0.0000 | 0.0000 | 0.0000 |
| 47 | 0.1667 |  | 210.0000 |  |  | 17.5035 | 2.9178 | 0.0000 |
| 47 | 0.5000 |  | 786.0000 |  |  | 183.4869 | 74.2452 | 0.0000 |
| 47 | 1.0000 |  | 1700.0000 |  |  | 804.9869 | 597.4952 | 0.0000 |
| 47 | 2.0000 |  | 2520.0000 |  |  | 2914.9869 | 3967.4952 | 0.0000 |
| 47 | 4.0000 |  | 1320.0000 |  |  | 6754.9869 | 14287.4952 | 0.0000 |
| 47 | 6.0000 |  | 630.0000 |  |  | 8704.9869 | 23347.4952 | 0.0000 |
| 47 | 8.0000 |  | 752.0000 |  |  | 10086.9869 | 33143.4952 | 0.0000 |
| 47 | 10.0000 |  | 362.0000 |  |  | 11200.9869 | 42779.4952 | 0.0000 |
| 47 | 12.0000 |  | 253.0000 |  |  | 11815.9869 | 49435.4952 | 0.0000 |
| 47 | 24.0000 |  | 73.2000 |  |  | 13773.1869 | 78192.2952 | 0.0000 |
| 47 | 36.0000 |  | 30.6000 |  |  | 14395.9869 | 95342.6952 | 0.0000 |
| 47 | 48.0000 | * | 20.4000 | 20.4369 | -0.0369 | 14701.9869 | 107827.4952 | 1.0000 |
| 47 | 72.0000 | * | 15.4000 | 15.3444 | 0.0556 | 15131.5869 | 132883.4952 | 1.0000 |
| 47 | 96.0000 | * | 11.5000 | 11.5208 | -0.0208 | 15454.3869 | 159437.0952 | 1.0000 |
| 48 | 0.0000 |  | 0.0000 |  |  | 0.0000 | 0.0000 | 0.0000 |
| 48 | 0.1667 |  | 220.0000 |  |  | 18.3370 | 3.0568 | 0.0000 |
| 48 | 0.5000 |  | 618.0000 |  |  | 157.9897 | 60.6634 | 0.0000 |
| 48 | 1.0000 |  | 1430.0000 |  |  | 669.9897 | 495.4134 | 0.0000 |
| 48 | 2.0000 |  | 1920.0000 |  |  | 2344.9897 | 3130.4134 | 0.0000 |
| 48 | 4.0000 |  | 1450.0000 |  |  | 5714.9897 | 12770.4134 | 0.0000 |
| 48 | 6.0000 |  | 666.0000 |  |  | 7830.9897 | 22566.4134 | 0.0000 |
| 48 | 8.0000 |  | 340.0000 |  |  | 8836.9897 | 29282.4134 | 0.0000 |
| 48 | 10.0000 |  | 380.0000 |  |  | 9556.9897 | 35802.4134 | 0.0000 |
| 48 | 12.0000 |  | 290.0000 |  |  | 10226.9897 | 43082.4134 | 0.0000 |
| 48 | 24.0000 |  | 94.8000 |  |  | 12535.7897 | 77613.6134 | 0.0000 |
| 48 | 36.0000 | * | 31.6000 | 29.9138 | 1.6862 | 13294.1897 | 98090.4134 | 1.0000 |
| 48 | 48.0000 | * | 20.0000 | 23.5913 | -3.5913 | 13603.7897 | 110676.0134 | 1.0000 |
| 48 | 72.0000 | * | 17.8000 | 14.6729 | 3.1271 | 14057.3897 | 137575.2134 | 1.0000 |
| 48 | 96.0000 | * | 8.4000 | 9.1259 | -0.7259 | 14371.7897 | 162631.2134 | 1.0000 |

X vs. Observed Y and Predicted Y

X vs. Observed Y and Predicted Y

X vs. Observed Y and Predicted Y

X vs. Observed Y and Predicted Y

X vs. Observed Y and Predicted Y

X vs. Observed Y and Predicted Y

X vs. Observed Y and Predicted Y

X vs. Observed Y and Predicted Y

X vs. Observed Y and Predicted Y

X vs. Observed Y and Predicted Y

X vs. Observed Y and Predicted Y

X vs. Observed Y and Predicted Y

X vs. Observed Y and Predicted Y

X vs. Observed Y and Predicted Y

X vs. Observed Y and Predicted Y

X vs. Observed Y and Predicted Y

X vs. Observed Y and Predicted Y

X vs. Observed Y and Predicted Y

X vs. Observed Y and Predicted Y

X vs. Observed Y and Predicted Y

X vs. Observed Y and Predicted Y

X vs. Observed Y and Predicted Y

X vs. Observed Y and Predicted Y

X vs. Observed Y and Predicted Y
